# Neurofilament light-associated connectivity in young-adult Huntington’s disease is related to neuronal genes

**DOI:** 10.1093/brain/awac227

**Published:** 2022-06-27

**Authors:** Peter McColgan, Sarah Gregory, Paul Zeun, Angeliki Zarkali, Eileanoir B Johnson, Christopher Parker, Kate Fayer, Jessica Lowe, Akshay Nair, Carlos Estevez-Fraga, Marina Papoutsi, Hui Zhang, Rachael I Scahill, Sarah J Tabrizi, Geraint Rees

**Affiliations:** Huntington’s Disease Centre, Department of Neurodegenerative disease, UCL Queen Square Institute of Neurology, University College London, London WC1N 3BG, UK; Huntington’s Disease Centre, Department of Neurodegenerative disease, UCL Queen Square Institute of Neurology, University College London, London WC1N 3BG, UK; Huntington’s Disease Centre, Department of Neurodegenerative disease, UCL Queen Square Institute of Neurology, University College London, London WC1N 3BG, UK; Dementia Research Centre, University College London, London WC1N 3AR, UK; Huntington’s Disease Centre, Department of Neurodegenerative disease, UCL Queen Square Institute of Neurology, University College London, London WC1N 3BG, UK; Department of Computer Science and Centre for Medical Image Computing, University College London, London WC1V 6LJ, UK; Huntington’s Disease Centre, Department of Neurodegenerative disease, UCL Queen Square Institute of Neurology, University College London, London WC1N 3BG, UK; Huntington’s Disease Centre, Department of Neurodegenerative disease, UCL Queen Square Institute of Neurology, University College London, London WC1N 3BG, UK; Huntington’s Disease Centre, Department of Neurodegenerative disease, UCL Queen Square Institute of Neurology, University College London, London WC1N 3BG, UK; Max Planck University College London Centre for Computational Psychiatry and Ageing Research, UCL Queen Square Institute of Neurology, London WC1N 3BG, UK; Huntington’s Disease Centre, Department of Neurodegenerative disease, UCL Queen Square Institute of Neurology, University College London, London WC1N 3BG, UK; Huntington’s Disease Centre, Department of Neurodegenerative disease, UCL Queen Square Institute of Neurology, University College London, London WC1N 3BG, UK; Dementia Research Centre, University College London, London WC1N 3AR, UK; Huntington’s Disease Centre, Department of Neurodegenerative disease, UCL Queen Square Institute of Neurology, University College London, London WC1N 3BG, UK; Huntington’s Disease Centre, Department of Neurodegenerative disease, UCL Queen Square Institute of Neurology, University College London, London WC1N 3BG, UK; Dementia Research Centre, University College London, London WC1N 3AR, UK; University College London Institute of Cognitive Neuroscience, University College London, London WC1N 3AZ, UK

**Keywords:** Huntington’s disease, connectivity, gene expression, neurofilament light

## Abstract

Upregulation of functional network connectivity in the presence of structural degeneration is seen in the premanifest stages of Huntington’s disease (preHD) 10–15 years from clinical diagnosis. However, whether widespread network connectivity changes are seen in gene carriers much further from onset has yet to be explored.

We characterized functional network connectivity throughout the brain and related it to a measure of disease pathology burden (CSF neurofilament light, NfL) and measures of structural connectivity in asymptomatic gene carriers, on average 24 years from onset. We related these measurements to estimates of cortical and subcortical gene expression.

We found no overall differences in functional (or structural) connectivity anywhere in the brain comparing control and preHD participants. However, increased functional connectivity, particularly between posterior cortical areas, correlated with increasing CSF NfL level in preHD participants. Using the Allen Human Brain Atlas and expression-weighted cell-type enrichment analysis, we demonstrated that this functional connectivity upregulation occurred in cortical regions associated with regional expression of genes specific to neuronal cells. This relationship was validated using single-nucleus RNAseq data from post-mortem Huntington’s disease and control brains showing enrichment of neuronal-specific genes that are differentially expressed in Huntington’s disease.

Functional brain networks in asymptomatic preHD gene carriers very far from disease onset show evidence of upregulated connectivity correlating with increased disease burden. These changes occur among brain areas that show regional expression of genes specific to neuronal GABAergic and glutamatergic cells.

## Teaser

McColgan *et al.* report that premanifest Huntington’s disease gene carriers around 20 years from disease onset display upregulated functional connectivity that correlates with increasing CSF neurofilament light. This occurs in brain regions that show gene expression specific to neuronal GABAergic and glutamatergic cells.

## Introduction

The earliest, asymptomatic stages of neurodegenerative disease involve a complex interplay between the effects of pathology on brain structure and function. Huntington’s disease is a monogenic, autosomal-dominant neurodegenerative disorder where both grey and white matter structural brain changes occur many years prior to disease onset.^1–4^ Alongside this, functional brain changes also occur,^5–11^ which may reflect the presence of pathological changes in connectivity^12^ or compensation in the form of upregulated brain network activity.^13–15^ However, the biological basis of these changes is unclear. Here, we investigate Huntington’s disease-related functional network connectivity during very early premanifest Huntington’s disease (preHD) in the context of largely intact structural white matter networks using regional gene expression and post-mortem Huntington’s disease single-nucleus RNA sequencing (snRNAseq) data.

Network connectivity in preHD gene carriers is upregulated within functional networks where structural connectivity is weakest.^12^ As such, marked axonal loss may lead to upregulated functional network connections unaffected by structural change or the recruitment of extra-network functional connections in those around 10–15 years from disease onset.^1,16–18^ We have previously shown that there are no detectable changes in structural network connectivity in a young-adult preHD cohort (HD-YAS) on average 24 years from disease onset,^19,20^ but there was evidence of functional network change.^11^ This is consistent with considerable evidence of neuronal network hyperexcitability driven by glutamatergic excitotoxicity and/or reduced inhibitory GABAergic activity in the earliest stages of neurodegeneration.^21–25^ However, our earlier study focused specifically on fronto-striatal circuits associated with cognitive flexibility and it is not known whether such functional network changes are more widespread. Determining this is important, because the period more than 20 years from clinical diagnosis is a point at which therapeutic treatments could potentially stall or eliminate disease progression.

Gene-expression profiles underlying patterns of functional connectivity have been investigated in healthy controls,^26^ neuropsychiatric cohorts^27,28^ and recently in Parkinson’s disease, where differential patterns of gene expression are associated with decoupling of structural and functional networks.^29^ In Huntington’s disease, gene transcription levels for synaptic signalling (particularly in the caudate and motor cortex) and cellular metabolism are atypical in human and animal models.^30,31^ Regions that show degeneration of white matter connections in preHD, around 15 years from disease onset, are also those that exhibit regional expression of synaptic and metabolic genes, particularly those that show abnormal transcription in post-mortem human and animal Huntington’s disease models.^18^ It is unclear, however, if brain network changes very far from disease onset show the same biological relationships and thus share a common pathobiology with later stage preHD or whether they are driven by different biological mechanisms.

In the current study, we characterized functional network connectivity in a cohort of asymptomatic young adult preHD gene carriers on average 24 years from disease onset.^19^ First, we sought to characterize pathology-related structural and functional network connectivity. We employed network-based statistics (NBS) to explore differences in network connectivity between preHD and controls and then tested the extent to which any changes were associated with Huntington’s disease pathology in terms of elevated CSF neurofilament light (NfL) levels, a marker of axonal degeneration that correlates with Huntington’s disease progression.^32–34^ We then investigated the possible mechanisms of any changes in connectivity using Allen Human Brain Atlas (AHBA) regional gene expression data and partial least squares regression. This provided ranked gene lists associated with regions that showed increased functional connectivity. We used these to perform gene ontology (GO) enrichment analyses to identify biological relationships and expression-weighted cell-type enrichment (EWCE) analyses to identify cell-specific relationships. Finally, the regional relationships we observed were validated using both differential gene-expression data from Huntington’s disease animal models and post-mortem Huntington’s disease brains and cell-specific snRNAseq data from Huntington’s disease and healthy post-mortem brains.

## Materials and methods

### Participants

Sixty-four preHD and 67 control participants matched for age, sex and education were recruited for the Huntington’s disease–Young Adult Study (HD-YAS).^19^ PreHD participants were gene-positive with a CAG repeat >39, Disease Burden Score <240^35^ and a Unified Huntington’s Disease Rating Scale Total Motor Score of ≤5.^36^ Control participants were gene-negative family members or individuals with no familial history of Huntington’s disease. Participants were excluded for recent drug or alcohol abuse and/or dependence, neurological or significant psychiatric comorbidity, brain trauma or contraindication to MRI. All participants underwent an extensive battery of cognitive and neuropsychiatric testing, clinical and medical history, neuroimaging, blood sampling and optional CSF collection.^19^ The study was approved by the local Research Ethics Committee and all participants gave written informed consent prior to study entry.

### Biofluid collection

Biofluids were acquired using standardized, validated conditions, methods and equipment.^37^ The NfL protein^32,33^ was collected from both CSF and blood plasma.

### MRI data acquisition

MRI data were acquired on a 3 T Prisma Scanner (Siemens Healthcare) with a 64-channel head coil. T_1_-weighted images were acquired using a 3D magnetization prepared rapid gradient echo (MPRAGE) sequence: repetition time (TR) = 2530 ms; echo time (TE) = 3.34 ms; inversion time (TI) = 1100 ms; flip angle = 7°; field of view = 256 × 256 × 176 mm^3^ with a resolution of 1.0 × 1.0 × 1.0 mm^3^. Diffusion weighted images were acquired using a multiband spin-echo echo planar imaging (EPI) sequence with TR = 3260 ms, TE = 58 ms, flip angle = 88°, field of view = 220 × 220 mm^2^. Seventy-two slices were collected with a resolution of 2 × 2 × 2 mm^3^. The multi-shell data consisted of *b*-values of 0 (*n* = 10, one with reverse phase-encoding), 100 (*n* = 8), 300 (*n* = 8), 1000 (*n* = 64) and 2000 (*n* = 64) s/mm^2^. Blip reversal acquisition parameters (used in topup) were the same as above. Resting-state functional MRI (fMRI) data were collected using a standard 2D EPI sequence: TR = 3.36 s; TE = 30 ms; 48 slices were acquired with 2.5 mm slice thickness with in-plane field of view of 192 × 192 mm^2^ with 3 × 3 mm^2^ resolution with 165 volumes. Field maps were collected to correct for inhomogeneity in the B0 field of the EPI fMRI images: TR = 1020 ms; TE1 = 10 ms; TE2 = 12.46 ms, 64 slices were acquired with 2 mm slice thickness with in-plane field of view of 192 × 192 mm^2^ with 3 × 3 mm^2^ resolution. Pulsatile information was collected using the Nonin 8600FO pulse-oximeter and a Siemens breathing belt for respiratory data. Both were recorded along with scanner pulses using Cambridge Electronics Device CED Micro 1401 Mk II connected to a laptop running Spike v2.

### MRI atlases

Cortical and subcortical atlases were derived from fMRI datasets and represent regions parcellated on this basis of their functional connections. For cortical regions, we used the Shaeffer cortical atlas,^38^ and to investigate the effects of parcellation granularity, we performed NBS and genetic analyses using both the 100 and 500 region parcellations. For striatal regions, we used the Choi atlas^39^ generated by assigning each voxel in the striatum to the most strongly correlated cortical region on the basis of its functional connectivity. Both atlases were registered to standard Montreal Neurological Institute space and then combined into one atlas, resulting in 114 and 514 region atlases.

### Diffusion processing

A white matter connectome was created for each participant using anatomically constrained tractography^40^ implemented in MRtrix.^41^ Raw diffusion images were first visually quality controlled. Denoising^42^ and Gibbs ringing artefact removal was performed^43^ using MRtrix. FSL eddy and topup were used to correct image distortions due to eddy current- and susceptibility-induced off-resonance fields and subject movement.^44^ B1 field inhomogeneity correction for the diffusion weighted imaging volume series was then performed using the ANTS N4 algorithm.^45^ Voxel-wise fibre orientation distribution was calculated using multi-shell multi-tissue constrained spherical deconvolution,^46^ with group-averaged response functions estimated for white matter, grey matter and CSF. Intensity normalization was then performed on fibre orientation distributions and probabilistic whole-brain tractography implemented to generate 10 million streamlines. Streamlines terminated when exiting the white matter. Spherical deconvolution informed filtering of tractograms (SIFT2) was used to remove biases inherent in tractography where longer connections are overdetermined, streamlines follow the straightest path and lack an associated volume.^47^ Connectomes were constructed by combining streamline tractograms with each participant’s combined cortical (100 and 500 regions of interest)/subcortical (14 regions of interest) parcellation and streamlines assigned to the closest region within a 2 mm radius of each end point. Structural connections were then weighted by streamline count and a cross-sectional area multiplier as per SIFT2.^48^ Connections were then combined into 114 × 114 and 514 × 514 undirected and weighted matrices.

### Functional MRI processing

Functional MRI data preprocessing and subsequent statistical analyses were performed using SPM12 running under MATLAB (ver R.2012b). The T_1_-weighted scan was segmented into grey and white matter during this process and DARTEL deformation parameters were created. The first five EPI images were discarded to allow for steady-state equilibrium. Functional images were slice-timing corrected and realigned, incorporating field maps for inhomogeneity correction, and coregistered to the T_1_ image. EPI images were then normalized using DARTEL deformation parameters and smoothed using a 6 mm full-width at half-maximum Gaussian kernel. Functional connectivity analyses were then performed using the CONN toolbox.^49^ Smoothed, normalized EPI images were included with corresponding structural images (combined, segmented grey and white matter). All EPI images were denoised using a bandpass filter 0.008–0.09 and linear detrending, movement parameters and signals from both white matter and CSF as a proxy for physiological measures were additionally regressed. Regression was performed before bandpass filtering, as is the default in the CONN toolbox. This avoids the reintroduction of motion artefacts^50^ or unwanted frequency components,^51^ which can occur when regression is performed after bandpass filtering. Connections were then combined into 114 × 114 and 514 × 514 undirected and weighted matrices, matching the structural connectivity matrices. This approach has been used both by our group^11^ and others.^52^ Simultaneous^50,51^ regression and bandpass filtering were also performed using the ‘simult’ option in CONN.

Motion parameters in six directions were derived for each individual following the realignment step that was performed as part of the fMRI data preprocessing pipeline. These motion parameters were subsequently included as a covariate of no interest in the first-level analyses for each participant. The motion-corrected data were then used at the second level with potential differences due to motion essentially removed. It should also be noted that while this was a movement-disordered patient group, all participants were asymptomatic and as such there was minimal effect of disease-related motion on the scans, each of which were quality-controlled prior to any preprocessing or analyses. Additionally, the maximum movement displacement was calculated for each subject and group differences were explored using a two-tailed *t*-test.

### Connectivity analyses

NBS version 1.2 (https://sites.google.com/site/bctnet/comparison/nbs) was used to investigate independently group differences in structural and functional connectivity^53^ using both the 114 and 514 parcellation matrices. Using this method, a test statistic is calculated for each connection independently. A primary threshold (*P* < 0.05, uncorrected) is then applied to form a set of suprathreshold connections. Permutation testing is then used to calculate a family-wise error (FWE) corrected *P*-value for each set of suprathreshold connections or subnetwork.^53^ Results reaching FWE corrected *P* < 0.05 are reported as significant, with *P*-values relating to the significance of all the connections within a subnetwork as a whole as opposed to individual connections. For these analyses, permutation testing using unpaired *t*-tests and 5000 permutations, as per the default NBS options, was performed on a general linear model that included age and gender as covariates. A test statistic was then computed for each connection and a default threshold applied (*t* = 3.1) to produce a set of suprathreshold connections that displayed significant between-group connectivity differences. FWE-correction was applied at *P* = 0.05.

To focus only on functional connections that have an underlying structural connection, the functional connectivity analysis was repeated constraining the functional connectome by the structural. Here, the functional matrix was simply multiplied by the binarized structural matrix to remove any functional connections that do not have supporting structural connections, and the NBS analysis repeated. Statistically significant group differences in connectome density, as defined by the sum of all weighted connections, were also investigated. This was performed for structural, functional and constrained connectomes using permutation testing (5000 permutations) with two-tailed *t*-tests including age and gender as covariates.

The relationship between NfL and connectivity may occur in a continuous manner such that higher NfL levels correlate with absent or reduced connectivity. Alternatively, connectivity changes may occur (or be detectable) only when a certain pathological threshold of NfL is reached. To test these two hypotheses, we performed two sets of NfL analyses.

First, we investigated the role of CSF NfL on structural and functional connectivity, correlating CSF NfL to structural and functional connections by including CSF NfL as the contrast in the NBS design matrix for the whole cohort, preHD only and control only.

Next, to investigate whether preHD participants with CSF NfL above a pathological threshold showed differences in structural or functional connectivity compared to preHD participants with normal CSF NfL, a subgroup analysis was performed where the preHD group was split in two on the basis of the CSF NfL results in the study. The low group had CSF NfL values within the 95th percentile of controls (<951 pg/ml), whereas the high group had CSF NfL values above this. This resulted in 24 gene carriers in the low group and 22 in the high group. The 95th percentile of controls was defined as the pathological threshold, in keeping with previous analyses using this cohort.^19^

### Gene expression analysis

#### Mapping gene expression data to MRI space

Gene expression microarray data were sourced from the Allen Human Brain Atlas (AHBA)^54^ to examine gene expression underlying the relationship between NfL and functional connectivity, as we identified significant association in our primary connectivity analyses. This contains gene expression data of 20 737 genes sampled across the adult brain. This atlas is based on data from six post-mortem human brains with no known neuropsychiatric or neuropathological history. Five donors were male and one was female with a mean age of 42.5 years. Three were Caucasian, two were African-American and one was Hispanic. AHBA data are freely available to download from the Allen Institute of Brain Science (AIBS, http://human.brain-map.org/static/download). The Abagen toolbox (https://github.com/rmarkello/abagen) was used to map gene expression data on to the combined cortex and striatum 114 regions of interest atlas. This toolbox follows optimized preprocessing steps previously reported.^55^ In brief, each tissue sample was assigned to one of the 114 regions of interest using AHBA MRI data for each donor. Data were pooled between homologous cortical regions (to ensure adequate bi-hemispheric coverage), with a 2 mm distance threshold on the cortical surface between samples. Probes with expression measures above background in over 50% of samples were selected, and a representative probe per gene was chosen based on highest intensity. Gene expression data were then normalized, leading to 15 633 genes included in the final gene dataset. In the AHBA, data for the left hemisphere were available for all donors, while two donors included right hemisphere data. Previous studies have used mirroring, were the left hemisphere data are mirrored on the right hemisphere in order to account for this.^56^ We opted not to perform mirroring, as this approach has a differential impact on statistical estimates in regional gene expression analyses.^57^

#### Statistical analysis: partial least squares regression

All statistical analysis was performed in MATLAB R2018b. Partial least squares (PLS) regression was used to reveal the biological and cell-specific mechanisms underlying the relationship between CSF NfL and functional connectivity. PLS regression is a multivariate technique used to identify associations between response and predictor variables. In our case, the predictor variable was a 114 regions of interest × 15 633 gene matrix.

Two complimentary approaches were used to generate the response variable, a partial correlation analyses in the preHD group only and a mixed linear model with a focus on the NfL × Group interaction. For the partial correlation analysis, graph theory strength was calculated, which equates to the sum of functional connectivity for each region of interest. Spearman rank partial correlations were then performed with NfL, controlling for age and gender. The partial correlations for each region of interest were then used as the response variable for the PLS. In this context, the focus is not on which correlations are significant but rather the spectrum of correlations across cortical regions of interest; similar approaches have been used in the literature to relate age, cortical thickness and regional gene expression.^58^ Strength was selected as a graph theory metric, as it is calculated by the sum of weighted connections to each region of interest. This allows comparison with the NBS analysis, which uses weighted connections as an input in the form of a connectivity matrix. While there is no current consensus within the literature as to the optimal graph theory metric for use in resting-state fMRI analyses, graph theory strength has higher test–retest reliability, as measured by interclass correlation coefficient, than other commonly used metrics such as clustering coefficient, betweenness centrality, local efficiency and degree.^59^

To explore the interaction between NfL and group, the following mixed linear model was used: region of interest functional connectivity strength ∼1 + Age + Gender + Group × NfL. The Group × NfL estimate for each region of interest was used as the response variable for the PLS. A mixed linear model was used as this is the most appropriate approach when including dependent variables, such as Group and NfL. In this context the focus is not on which model estimates are significant but rather the spectrum of negative and positive estimates across cortical regions of interest; similar approaches have been used in the literature to relate age, cortical thickness, magnetization transfer ratio and regional gene expression.^60^

Partial correlations, model estimates and spatial patterns of the weights of the PLS components were visualized using the BrainNet viewer (https://www.nitrc.org/projects/bnv) for combined cortical and subcortical visualizations and ggseg (https://github.com/ggseg/ggsegSchaefer) for visualizations of cortical surface only.

We performed spatial permutation testing to assess whether PLS results explained a significantly higher proportion of variance for each of our chosen response variables (partial correlation in the preHD group and group × NFL interaction, assessed separately) than expected by chance. To do this, we reordered the predictor matrix in terms of regions of interest based on sphere rotations^61^ and repeated the PLS regression using this predictor variable; this process was repeated for 1000 random permutations to construct a spatially correlated null distribution of PLS weights in keeping with the literature.^29,61,62^*P*-values for PLS components have been calculated based on the explained variance in the observed data relative to the variance explained in the null model. To quantify the spatial topography of PLS weights and enable comparison between the 114 and 514 region of interest atlases, Spearman rank correlations were performed between the MRI coordinates (left to right, A: posterior to anterior, S: inferior to superior) and PLS weights, for the 114 and 514 region of interest NfL partial correlation analyses.

As the greatest amount of variance was explained by the first PLS component (PLS1), genes were ranked based on their contribution to this component. Permutation testing was used to assess whether genes were weighted higher or lower than expected by chance, correcting for FWE. Similar to the NBS implementation, the one-sided FWE-corrected *P*-value (*q*-value) for a gene is estimated as the proportion of permutations for which the weighting of this gene is higher than the 95th percentile or lower than the 5th percentile of the spatially correlated null distribution. Only genes with weights significantly higher or lower than expected by chance (*q* < 0.05) were included in the subsequent GO enrichment analysis. Genes with negative (downweighted) and positive (upweighted) PLS weightings were ranked separately. There are several previous studies that used PLS for the large gene-expression datasets from the AHBA.^18,29,63^

#### Gene ontology enrichment analysis

To investigate the genetic basis underlying the CSF NfL and functional connectivity associations, we performed enrichment analysis for GO, Kyoto Encyclopedia of Genes and Genomes pathway, Reactome and CORUM terms using g:Profiler to identify GO terms that were significantly enriched in the top (upweighted) and bottom (downweighted) genes from the PLS1 ranked gene list. Only genes that were significantly more up- or downweighted than expected by chance (against a spatially correlated null distribution) were included in this analysis. A Benjamini–Hochberg correction for multiple comparisons was used with a significance threshold of 0.05, as implemented in g:Profiler. To aid interpretation, we removed general GO terms by excluding those with greater than 1000 genes in their classification in keeping with other studies in the literature.^63,64^ This allowed us to focus on specific gene sets as opposed to GO terms encompassing thousands of genes covering a range of processes.

#### Expression-weighted cell-type enrichment analysis

To investigate whether specific cell types were associated with CSF NfL and functional connectivity, we performed EWCE.^65^ The top (upweighted) and bottom (downweighted) 10%, 20% and 30% of genes from the PLS1 ranked gene list were used as target lists. Incremental thresholds were chosen to identify the most significant cell-type association for each target list. Each was run with 100 000 bootstrap lists, controlling for transcript length and GC content, which can bias genetic enrichment analyses,^66^ using only major cell-type classes (e.g. ‘astrocyte’, ‘microglia’, etc.). The Benjamini–Hochberg method was used for correction of multiple comparisons as is the default in EWCE software. Single-cell transcription data were used from the AHBA (https://portal.brain-map.org/atlases-and-data/rnaseq) containing data from the middle temporal gyrus.^67^ To ensure that our results were not dependent on the dataset used, we replicated our EWCE analysis with the same parameters (100 000 bootstrap lists, Benjamini–Hochberg correction) using a different human-derived dataset from Habib *et al*.^68^; this is a comprehensive human derived post-mortem dataset, containing data from five donors and 19 550 cells from both the hippocampus and the prefrontal cortex. The EWCE package is freely available from https://github.com/NathanSkene/EWCE.

#### Enrichment analysis of striatal and cortical genes showing abnormal transcription in Huntington’s disease

We then investigated whether striatal and cortical genes showing abnormal transcription in human and animal models of Huntington’s disease were enriched greater than by chance in the ranked gene list of the PLS1. Huntington’s disease gene lists were obtained from Langfelder *et al*.,^69^ which consists of genes that show consistent differences in Huntington’s disease compared to controls both in the Huntington’s disease knockout mouse allelic series^69^ and human Huntington’s disease post-mortem data from the caudate nucleus^70^ and cortical regions Brodmann areas 4 and 9,^31^ prefrontal and visual cortices.^71^ To test whether these gene lists were enriched greater than by chance in the PLS1, we performed a permutation test of the normalized bootstrap weight of each gene in the PLS1 summed over all genes for each gene list. The approach has been used previously,^18,63^ and the code is freely available at https://github.com/KirstieJane/NSPN_WhitakerVertes_PNAS2016/blob/master/SCRIPTS/PLS_candidate_genes.m.

#### Enrichment analysis of cell-specific genes showing abnormal transcription in snRNAseq in Huntington’s disease

**Figure 1:**
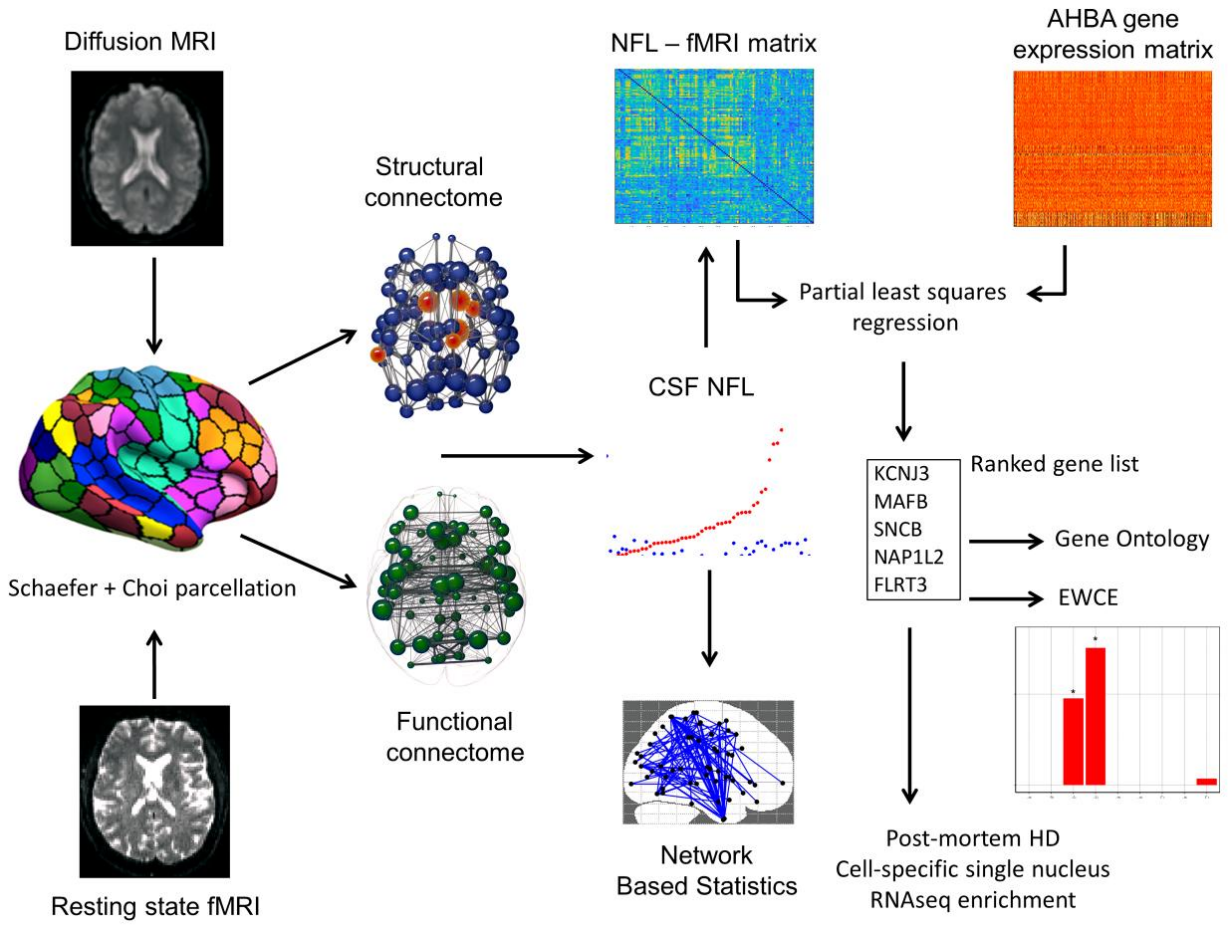
**Summary of analysis pipeline.** Diffusion MRI and resting-state fMRI underwent preprocessing and were parcellated using the Shaefer cortical atlas^38^ and the Choi subcortical atlas.^39^ Structural and functional connectomes were then created based on weighted streamlines between brain regions and temporal fMRI time-series correlations between regions, respectively. Correlations were performed between CSF NfL and brain networks using NBS. An NfL–fMRI correlation matrix was also used to investigate associations with regional gene expression using the AHBA. PLS regression produced a ranked gene list of those genes most strongly associated with NfL–fMRI hyperconnectivity. GO and EWCE were then used to investigate biological and cell-specific associations. Finally these results were validated using snRNAseq^72^ from post-mortem Huntington’s disease (HD) and control brains.

To relate cell-specific CSF NfL–fMRI relationships to Huntington’s disease pathology, we utilized data from a study analysing snRNAseq data in post-mortem Huntington’s disease brains relative to controls.^72^ Single nucleus RNAseq can be applied to frozen post-mortem brain tissue and thus overcomes limitations of single-cell (sc)RNAseq approaches, which cannot be applied to frozen tissue. This enables the identification of cell-specific genes that show abnormal transcription in Huntington’s disease. Al-Dalahmah *et al*.^72^ analysed snRNA seq data from samples of the anterior cingulate cortex frozen at post-mortem in four cases (two Huntington’s disease and two controls) from the New York Brain Bank. In doing so, they provided lists of neuron- and astrocyte-specific genes that signify different levels of transcription in Huntington’s disease relative to controls. We tested whether these gene lists were enriched greater than by chance in the PLS1 from the above gene CSF NfL-functional connectivity analysis using the permutation test described above. See Fig. 1 for a summary of the methodical approach.

### Data and code availability

Anonymized, derived data supporting the findings of this study are available from the corresponding author on request. Code used to implement analyses in this study is freely available at https://github.com/AngelikaZa/YAS_HD.

## Results

### Demographic and clinical data

There were no significant differences in age [*t*(85) = 0.7, *P* = 0.49] between controls (mean = 28.61, SD = 5.68) and gene carriers (mean = 29.46, SD = 5.62), sex (χ^2^, *P* = 0.67) between controls (females = 23, males = 18) and gene carriers (female = 23, male = 23) or International Standard Classification of Education (χ^2^, *P* = 0.45). However, CSF NfL levels were significantly different [*t*(85) = 4.2, *P* = 0.0001] between controls (mean = 354, SD = 261) and gene carriers (mean = 767, SD = 585).

### Structural and functional connectivity

There were no significant between-group differences in structural or functional connectivity (with and without constraining by structural connectome) for either the 114 or 514 parcellation analyses (Table 1). No connectome density group differences were observed for structural (114 regions of interest, *P* = 0.52; 514 regions of interest, *P* = 0.66), functional (114 regions of interest, *P* = 0.65; 514 regions of interest, *P* = 0.64) or constrained connectomes (114 regions of interest, *P* = 0.58; 514 regions of interest, *P* = 0.83). For resting-state fMRI, there was no significant difference (*P* = 0.096) in maximum movement displacement between preHD (mean = 0.75, SD = 0.49) and controls (mean = 0.62, SD = 0.24).

**Table 1:** Network-based statistics results

Group *t*-test	PreHD < Controls	Cont < PreHD
Functional (114 ROIs)	0.672	0.6046
Structural (114 ROIsr)	0.3447	0.2384
Functional (structural constrained) 114 ROIsr	0.6072	0.5792
Functional (514 ROIsr)	0.4296	0.5976
Structural (514 ROIsr)	0.1504	0.4613
Functional (structural constrained) 514 ROIsr	0.2603	0.7998
**Correlations**	**Positive**	**Negative**
NFL–fMRI correlation (whole group) 114 ROIsr	0.0304*	1
NFL–fMRI correlation (preHD) 114 ROIsr	0.019*	0.6633
NFL–structural correlation (whole group) 114 ROIsr	0.191	0.391
NFL–structural correlation (preHD) 114 ROIsr	0.2523	0.1818
NFL–fMRI correlation (whole group) 514 ROIsr	0.0398*	0.6615
NFL–fMRI correlation (preHD) 514 ROIsr	0.027*	0.6478
NFL–structural correlation (whole group) 514 ROIsr	0.3177	0.0284*
NFL–structural correlation (preHD) 514 ROIsr	0.2919	0.023*

For these analyses, permutation testing using unpaired *t*-tests and 5000 permutations was performed on a general linear model that included age and sex as covariates. A test statistic was then computed for each connection and a default threshold applied (*t* = 3.1) to produce a set of suprathreshold connections that displayed significant between-group connectivity differences. *FWE-correction was applied at *P* = 0.05. Due to the high false positive rates in fMRI connectivity analyses^73^ the functional connectivity analysis was repeated, constraining the functional connectome by the structural. Here, the functional matrix was simply multiplied by the structural matrix to remove any functional connections that do not have supporting structural connections, and the NBS analysis repeated. ROIs = regions of interest.

### Relationship between CSF NfL and structural and functional connectivity in preHD

A three-way ANOVA was performed to compare structural and functional connectivity using 114 parcellations between three groups: controls, preHD with normal CSF NfL and preHD with higher CSF NfL. No significant differences were found for either structural (*P^FWE^* = 0.25) or functional (*P^FWE^* = 0.97) connectivity.

For structural connectivity, there were no significant correlations between CSF NfL for controls and preHD combined or preHD only for the 114 parcellation. There were significant negative correlations for both controls and preHD combined (*P^FWE^* = 0.028) and preHD only (*P^FWE^* = 0.023) for the 514 parcellation (Supplementary Figs 1 and 2).

**Figure 2:**
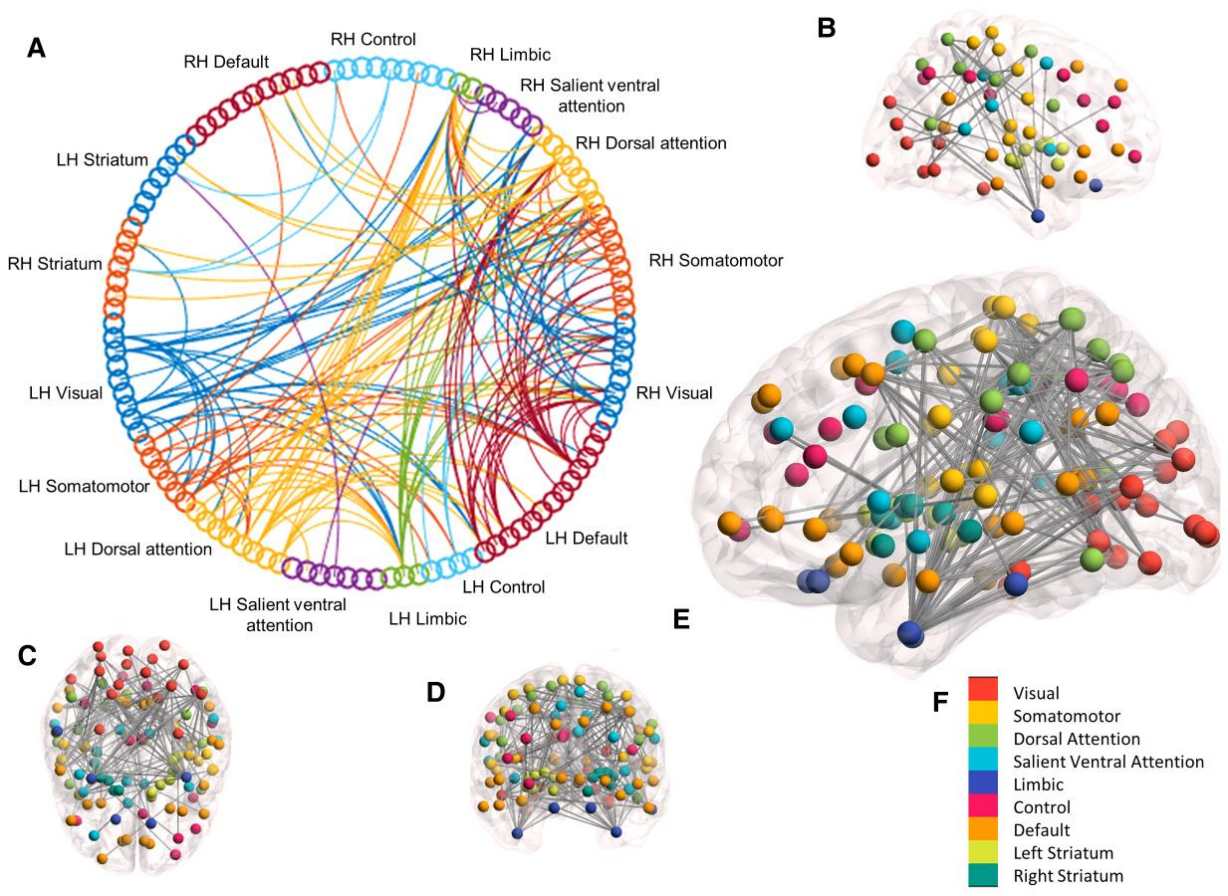
**Resting-state fMRI brain subnetwork showing significant (*P* < 0.05 FWE-corrected) positive correlation with CSF NfL across Huntington’s disease gene expansion carriers.** Analysis performed using NBS. (**A**) Circular graph depicting significant subnetwork. (**B**) Left sagittal view. (**C**) Axial view. (**D**) Coronal view. (**E**) Right sagittal. (**F**) Colour scheme for brain figures. Spheres indicate brain regions; lines indicate fMRI connections that correlate with NfL between brain regions. LH = left hemisphere; RH = right hemisphere.

For functional connectivity, there were significant positive correlations between CSF NfL and functional connectivity for both the combined (*P^FWE^* = 0.034) and preHD group only (*P^FWE^* = 0.019) for the 114 parcellation (Table 1 and Fig. 2) and for both the combined (*P^FWE^* = 0.04) and preHD group only (*P^FWE^* = 0.027) for the 514 parcellation (Supplementary Figs 1 and 2). The connections of the subnetwork that showed a positive correlation with NfL were located predominantly in the posterior cortex, with very few anterior regions affected. Of the 164 connections in the preHD group-only subnetwork, 6% were cortico-striatal, 49% interhemispheric and 45% intrahemispheric (Table 2 and Supplementary Table 2). There were no significant negative correlations between CSF NfL and functional connectivity (Table 1).

**Table 2:** Network-based statistics subnetwork showing significant correlation with CSF NfL for preHD gene carriers

	Connection 1	Connection 2	T-stat
Cortical–striatal	7Networks_RH_Cont_PFCl_3	R_Ventral_attention	4.06
	7Networks_LH_SalVentAttn_PFCl_1	L_Dorsa_lattention	3.95
	7Networks_RH_DorsAttn_Post_4	R_Somatomotor	3.88
	7Networks_RH_Cont_pCun_1	R_Ventral_attention	3.51
	7Networks_RH_DorsAttn_Post_4	L_Somatomotor	3.44
	7Networks_RH_DorsAttn_Post_1	L_Somatomotor	3.42
	7Networks_RH_Cont_PFCl_3	L_Dorsal_attention	3.27
	7Networks_RH_DorsAttn_Post_5	R_Frontoparietal	3.24
	7Networks_LH_Vis_7	R_Somatomotor	3.18
	7Networks_RH_DorsAttn_Post_4	R_Ventral_attention	3.15
Interhemispheric	7Networks_LH_Default_Temp_1	7Networks_RH_DorsAttn_Post_5	5.24
	7Networks_LH_Cont_pCun_1	7Networks_RH_SomMot_6	5.13
	7Networks_LH_DorsAttn_Post_5	7Networks_RH_Limbic_TempPole_1	4.9
	7Networks_LH_Default_Par_1	7Networks_RH_Vis_2	4.79
	7Networks_LH_Cont_pCun_1	7Networks_RH_SomMot_2	4.76
	7Networks_LH_DorsAttn_FEF_1	7Networks_RH_Limbic_TempPole_1	4.74
	7Networks_LH_Default_PFC_1	7Networks_RH_DorsAttn_Post_5	4.71
	7Networks_LH_Limbic_TempPole_1	7Networks_RH_DorsAttn_Post_5	4.65
	7Networks_LH_Cont_pCun_1	7Networks_RH_SomMot_5	4.64
	7Networks_LH_SomMot_6	7Networks_RH_Limbic_TempPole_1	4.58
Intrahemispheric	7Networks_RH_SomMot_6	7Networks_RH_DorsAttn_Post_1	6.78
	7Networks_LH_DorsAttn_Post_6	7Networks_LH_Default_Temp_1	4.86
	7Networks_RH_Vis_2	7Networks_RH_SomMot_8	4.84
	7Networks_LH_Vis_7	7Networks_LH_Limbic_TempPole_1	4.64
	7Networks_RH_DorsAttn_Post_5	7Networks_RH_Limbic_TempPole_1	4.53
	7Networks_RH_SomMot_8	7Networks_RH_DorsAttn_Post_1	4.51
	7Networks_LH_DorsAttn_Post_6	7Networks_LH_Limbic_TempPole_1	4.5
	7Networks_LH_DorsAttn_Post_3	7Networks_LH_Limbic_TempPole_1	4.49
	7Networks_LH_SomMot_6	7Networks_LH_Default_PFC_6	4.41
	7Networks_LH_Vis_3	7Networks_LH_Limbic_TempPole_1	4.39

Connections classified as cortico-striatal, interhemispheric, intrahemispheric and ranked based on test statistic. Top 10 connections based on test statistic (T-stat) displayed for each connection type.

No significant correlations were seen between NfL and either structural or functional connectivity for the control group only (Supplementary Table 1), suggesting the absence of a physiological relationship between NfL and brain networks. Replication of the significant fMRI analyses using the CONN ‘simult’ processing option did not reveal significance and may be related to less specificity with this processing option (Supplementary Table 1).

### Region of interest partial correlation, mixed linear model and PLS analyses

For the partial correlation of 114 regions of interest analysis, one brain region showed false discovery rate (FDR)-corrected significance (*q*), left DorsAttn_Post_6 (rho = 0.38, *q* = 0.031). A further seven nodes from the dorsal attention and visual networks showed uncorrected (*P* < 0.05) significance, all with positive correlations. See Supplementary Table 3 and Supplementary Figs 3–5 for visualizations of correlations. Using the region of interest correlations as an input into the PLS analysis, the first component explained the largest amount of variance at 22.5%, *P* < 1 × 10^−10^ [2nd, 8% (*P* = 0.49); 3rd, 15.4% (*P* = 0.005); 4th, 10.1% (*P* = 0.24); 5th, 9% (*P* = 0.36)]. The spatial patterns of the weights of the PLS1 are visualized in Supplementary Figs 6–8. The spatial topography analysis revealed correlations between PLS weights and R (left to right): rho = 0.02, *P* = 0.83, A (posterior to anterior): rho = −0.35, *P* = 0.0004, S (inferior to superior): rho = 0.42, *P* = 1.3 × 10^−5^.

For the mixed linear model analysis uncorrected significance for the group × NfL interaction was seen for right Default_pCunPCC_2 (β = −0.008, *P* = 0.013), right Limbic_TempPole_1 (β = 0.008, *P* = 0.027) and right Vis_7 (β = 0.01, *P* = 0.031). See Supplementary Table 4 and Supplementary Figs 9–11 for visualizations of model estimates. Using the region of interest Group × NfL interaction estimate as an input into the PLS analysis, the first component explained the largest amount of variance at 24.7%, *P* = 0.047 [2nd, 6.1% (*P* = 0.29); 3rd, 8.9% (*P* = 0.41); 4th, 8.6% (*P* = 0.4); 5th, 10.1% (0.47)]. The spatial patterns of the weights for the PLS1 are visualized in Supplementary Figs 12 and 14.

For the partial correlation of 514 regions of interest analysis, no brain regions showed FDR-corrected significance. Ninety-seven nodes from the dorsal attention, visual, somatomotor, limbic and default mode, salient ventral attention and control networks showed uncorrected (*P* < 0.05) significance (91 positive correlations and six negative correlations). Consistent with the 114 regions of interest analysis, dorsal attention and visual regions of interest were among the most significant. See Supplementary Table 5 and Supplementary Fig. 15 for visualizations of correlations.

Using the region of interest correlations as an input into the PLS analysis, the first component explained the largest amount of variance at 13.03%, *P* = 0.014. Downweighted but no upweighted genes were identified in the first component; therefore, the second component was also investigated in subsequent analyses. The spatial pattern of the weights for the first PLS component are visualized in Supplementary Fig. 16. The spatial topography analysis revealed correlations between PLS weights and R (left to right): rho = 0.18, *P* = 5.5 × 10^−5^, A (posterior to anterior): rho = −0.42, *P* = 2.2 × 10^−16^, S (inferior to superior): rho = 0.43, *P* = 2.2 × 10^−16^.

The second component explained 9.14% of the variance, *P* = 0.014 [3rd, 2.98% (*P* = 0.41); 4th, 7.92% (*P* = 0.036); 5th, 5.86% (*P* = 0.12)]. The spatial pattern of the weights for the second PLS component (PLS2) are visualized in Supplementary Fig. 17. The spatial topography analysis revealed correlations between PLS weights and R (left to right): rho = −0.21, *P* = 1.2 × 10^−6^, A (posterior to anterior): rho = −0.35, *P* = 5.8 × 10^−16^, S (inferior to superior): rho = 0.23, *P* = 2.2 × 10^−7^. Based on spatial topography, correlations for both 114 and 514 regions of interest analyses showed higher PLS weights in posterior and superior cortical regions of interest (Supplementary Figs 18–20). To facilitate comparisons between the 114 and 514 regions of interest across NBS, GO, EWCE and Huntington’s disease gene enrichment analyses, Supplementary Table 13 summarizes the results across the analyses.

### Gene ontology enrichment analysis

For the results using the ranked gene list from the partial correlation of 114 regions of interest PLS, the five most significant ontology terms for upweighted genes included presynapse (*P* = 4.84 × 10^−9^), somatodendritic compartment (*P* = 6.85 × 10^−9^), synaptic membrane (*P* = 1.75 × 10^−9^), potassium ion transmembrane transporter activity (*P* = 2.11 × 10^−8^) and presynaptic membrane (*P* = 3.93 × 10^−8^) (see Table 3). For downweighted genes, the five most significant ontology terms included cell morphogenesis involved in differentiation (*P* = 0.0003), I band (*P* = 0.003), phosphatidylinositol-4,5-bisphosphate binding (*P* = 0.004), camera-type eye development (*P* = 0.006) and cell morphogenesis involved in neuron differentiation (*P* = 0.007) (see Table 3).

**Table 3:** Top 5 significant GO terms for upweighted and downweighted genes from the partial correlation analysis (*P*cor) and the mixed linear model (Group × NfL) interaction analysis

Term name	Term ID	Source	*P*-value
Upweighted (*P*cor)			
Presynapse	GO:0098793	GO:CC	4.84 × 10^−9^
Somatodendritic compartment	GO:0036477	GO:CC	6.85 × 10^−9^
Synaptic membrane	GO:0097060	GO:CC	1.75 × 10^−8^
Potassium ion transmembrane transporter activity	GO:0015079	GO:MF	2.11 × 10^−8^
Presynaptic membrane	GO:0042734	GO:CC	3.93 × 10^−8^
Downweighted (*P*cor)			
Cell morphogenesis involved in differentiation	GO:0000904	GO:BP	0.0002733
I band	GO:0031674	GO:CC	0.0028901
Phosphatidylinositol-4,5-bisphosphate binding	GO:0005546	GO:MF	0.0036894
Camera-type eye development	GO:0043010	GO:BP	0.0055427
Cell morphogenesis involved in neuron differentiation	GO:0048667	GO:BP	0.0066415
Upweighted (Group × NfL)			
Microtubule organizing centre	GO:0005815	GO:CC	0.0008331
Plasma membrane-bounded cell projection assembly	GO:0120031	GO:BP	0.0012018
Cell projection assembly	GO:0030031	GO:BP	0.0018766
Centrosome	GO:0005813	GO:CC	0.0029856
Cilium organization	GO:0044782	GO:BP	0.0040246
Downweighted (Group × NfL)			
Presynapse	GO:0098793	GO:CC	2.13 × 10^−14^
Axon	GO:0030424	GO:CC	3.07 × 10^−9^
Anterograde trans-synaptic signalling	GO:0098916	GO:BP	3.31 × 10^−9^
Chemical synaptic transmission	GO:0007268	GO:BP	3.31 × 10^−9^
Trans-synaptic signalling	GO:0099537	GO:BP	5.36 × 10^−9^

GO:BP = gene ontology biological process; GO:CC = gene ontology cellular component; GO:MF = gene ontology molecular function; REAC = reactome.

Results using the ranked gene list from the Group × NfL interaction for downweighted genes were similar were similar to the upweighted ontology terms for the partial correlation analysis. The five most significant ontology terms for downweighted genes included presynapse (*P* = 2.13 × 10^−14^), axon (*P* = 3.07 × 10^−9^), anterograde trans-synaptic signalling (*P* = 3.31 × 10^−9^), chemical synaptic transmission (*P* = 3.31 × 10^−9^) and trans-synaptic signalling (*P* = 5.36 × 10^−9^). The five most significant ontology terms for upweighted genes included microtubule organizing centre (*P* = 0.0008), plasma membrane-bounded cell projection assembly (*P* = 0.001), cell projection assembly (*P* = 0.002), centrosome (*P* = 0.003) and cilium organization (*P* = 0.004) (see Table 3).

For the results using the ranked gene list from the partial correlation of 514 regions of interest PLS, for the PLS1, there were no upweighted genes; therefore, we included upweighted genes from the second component. Significant ontology terms for upweighted genes (component 2) included overlap with terms reported in for upweighted genes in the partial correlation of 114 regions of interest PLS analyses; these included potassium ion transmembrane transporter activity (*P* = 1.03 × 10^−8^), presynapse (*P* = 6.12 × 10^−6^) and somatodendritic compartment (*P* = 9.49 × 10^−5^). Significant ontology terms for downweighted genes (component 1) included similar synaptic and ion channel gene terms, such as presynapse (9.17 × 10^−6^), trans-synaptic signalling (*P* = 2.59 × 10^−5^) and ion transmembrane transporter activity (*P* = 1.54 × 10^−5^). GO lists for the 50 most significant terms, for all analyses, are included in Supplementary Table 6.

### Cell-specific enrichment analysis

For the cell enrichment analyses, we focused on the top and bottom 10% of genes. Results were consistent across databases [AIBS 2019 and DRONC (droplet-based single-nucleus RNA sequencing)] and 10–30% gene lists (Supplementary Tables 6 and 12).

**Figure 3:**
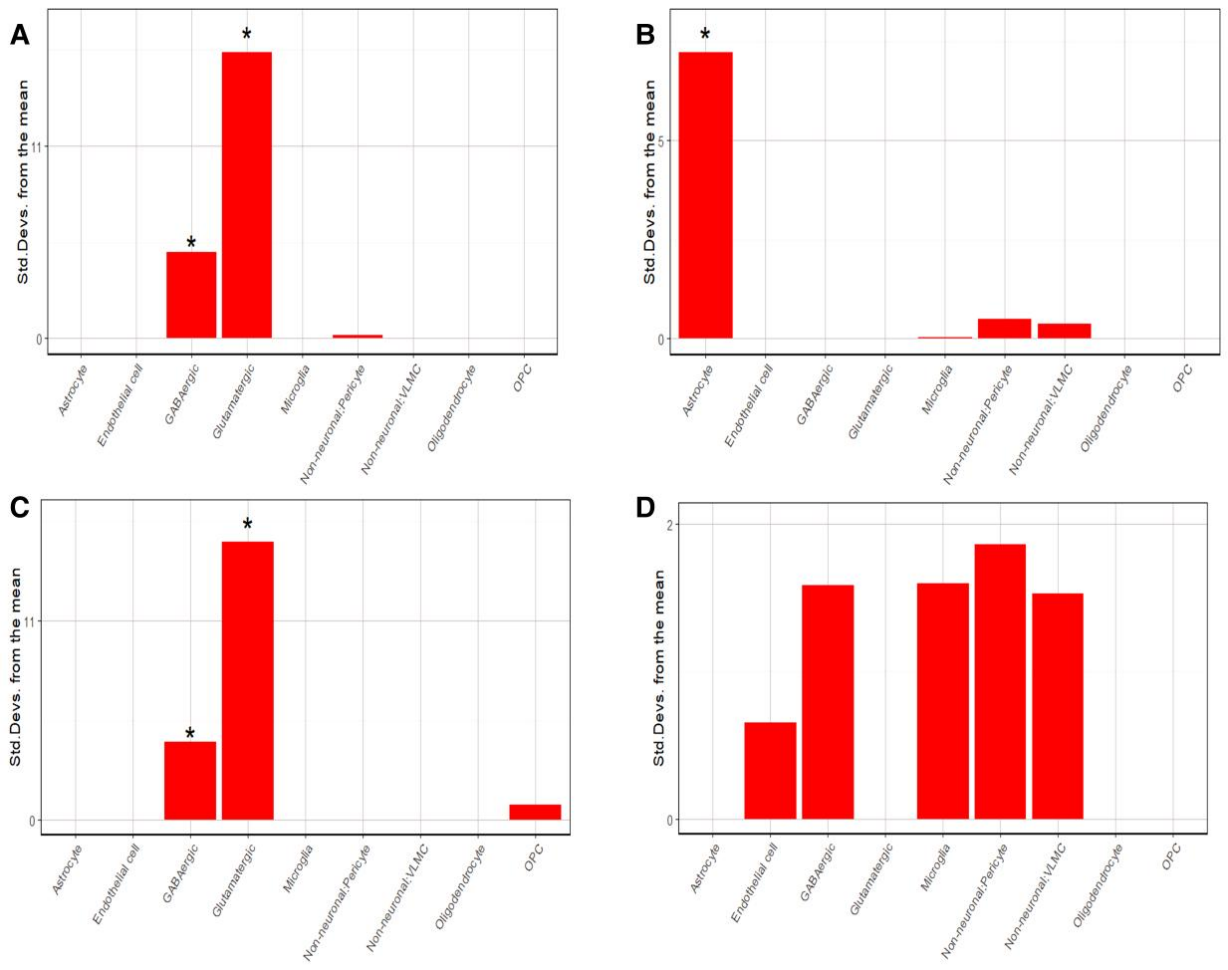
**EWCE analysis using AHBA 2019 cell-specific gene annotation.** (**A**) Top 10% upweighted genes for the partial correlation analysis. (**B**) Top 10% downweighted genes for the partial correlation analysis. (**C**) Top 10% downweighted genes for the Group × NfL analysis. (**D**) Top 10% upweighted genes for the Group × NfL analysis. *Corrected significance. OPC = oligodendrocyte precursor cells; VLMC = vascular leptomeningeal cells.

For results using the ranked gene list from the partial correlation of 114 regions of interest analysis, upweighted genes were significantly associated with neuronal cell types, while downweighted genes were significantly associated with glial cell types. For 10% upweighted, AIBS 2019 showed significance for glutamatergic (*P* < 1 × 10^−10^) and GABAergic cells (*P* = 2 × 10^−5^), while 10% downweighted showed significance for astrocytes (*P* < 1 × 10^−10^) (Table 4 and Fig. 3). The neuronal and glial cell split between up- and downweighted genes was replicated using the DRONC database. Full results are included in Supplementary Tables 7 and 8.

**Table 4:** EWCE analysis

Cell type	*P*-value	Fold change	SD from mean
Upweighted 10% (*P*cor)			
Glutamatergic	<1 × 10^−10^	2.89657	16.33697
GABAergic	0.00002	1.53571	4.95972
Non-neuronal: pericyte	0.39915	1.03437	0.20818
OPC	0.85648	0.87782	−1.04623
Microglia	0.98576	0.71634	−1.98875
Non-neuronal: VLMC	0.99380	0.57872	−2.21417
Endothelial cell	0.99947	0.57450	−2.78966
Astrocyte	0.99999	0.56620	−3.54350
Oligodendrocyte	1.00000	0.49659	−3.80922
Downweighted 10% (*P*cor)			
Astrocyte	<1 × 10^−10^	1.90801	7.21517
Non-neuronal: pericyte	0.29683	1.08003	0.49469
Non-neuronal: VLMC	0.33726	1.07361	0.37335
Microglia	0.46827	1.00395	0.02779
OPC	0.52067	0.98585	−0.12059
Endothelial cell	0.80142	0.86733	0.86733
GABAergic	0.84056	0.89153	−0.99208
Oligodendrocyte	0.99660	0.69286	−2.32499
Glutamatergic	0.99942	0.67336	−2.82282
Upweighted 10% (Group × NfL)			
Non-neuronal: pericyte	0.03905	1.28857	1.85843
Microglia	0.06393	1.21547	1.59846
GABAergic	0.06428	1.16696	1.58190
Non-neuronal: VLMC	0.06945	1.26748	1.52874
Endothelial cell	0.24742	1.09724	0.65320
Oligodendrocyte	0.77574	0.90052	−0.78095
Glutamatergic	0.87605	0.87073	−1.13485
OPC	0.97856	0.79741	−1.84178
Astrocyte	0.99049	0.74715	−2.08759
Downweighted 10% (Group × NfL)			
Glutamatergic	<1 × 10^−10^	2.832749	15.3434863
GABAergic	0.0001	1.4754347	4.27832
OPC	0.20687	1.0899718	0.7888829
Non-neuronal: VLMC	0.98562	0.6028899	−1.9850374
Endothelial cell	0.98776	0.6848202	−2.026815
Non-neuronal: pericyte	0.99747	0.5892623	−2.454821
Astrocyte	0.99978	0.6348877	−2.9696604
Oligodendrocyte	0.9999	0.6004525	−2.9841701
Microglia	0.99993	0.5594399	−3.0422093

Results for top 10% upweighted and downweighted genes using AHBA 2019 cell-specific gene classification.

OPC = oligodendrocyte precursor cells; *P*cor = corrected significance; SD = standard deviation; VLMC = vascular leptomeningeal cells.

Results using the ranked gene list from the Group × NfL interaction for downweighted genes were similar to the upweighted gene results for the partial correlation analysis. Downweighted genes were significantly associated with neuronal cell types, while upweighted genes were significantly associated with glial cell types. For 10% downweighted, AIBS 2019 showed significance for glutamatergic cells (*P* < 1 × 10^−10^) and GABAergic (*P* = 1 × 10^−4^), while 10% upweighted showed significance for pericyte (*P* = 0.04) (Table 4 and Fig. 3). The neuronal and glial cell split between up- and downweighted genes was replicated using the DRONC database (Supplementary Tables 9 and 10).

For results using the ranked gene list from the partial correlation of 514 regions of interest analysis 10% upweighted, AIBS 2019 showed significance for GABAergic (*P* < 1 × 10^−10^) and glutamatergic cells (*P* < 1 × 10^−10^), while 10% downweighted showed significance for astrocyte (*P* < 1 × 10^−10^) and GABAergic cells (*P* = 0.002). Full results are included in Supplementary Tables 11 and 12.

### Enrichment analysis of striatal and cortical genes showing abnormal transcription in Huntington’s disease

**Figure 4:**
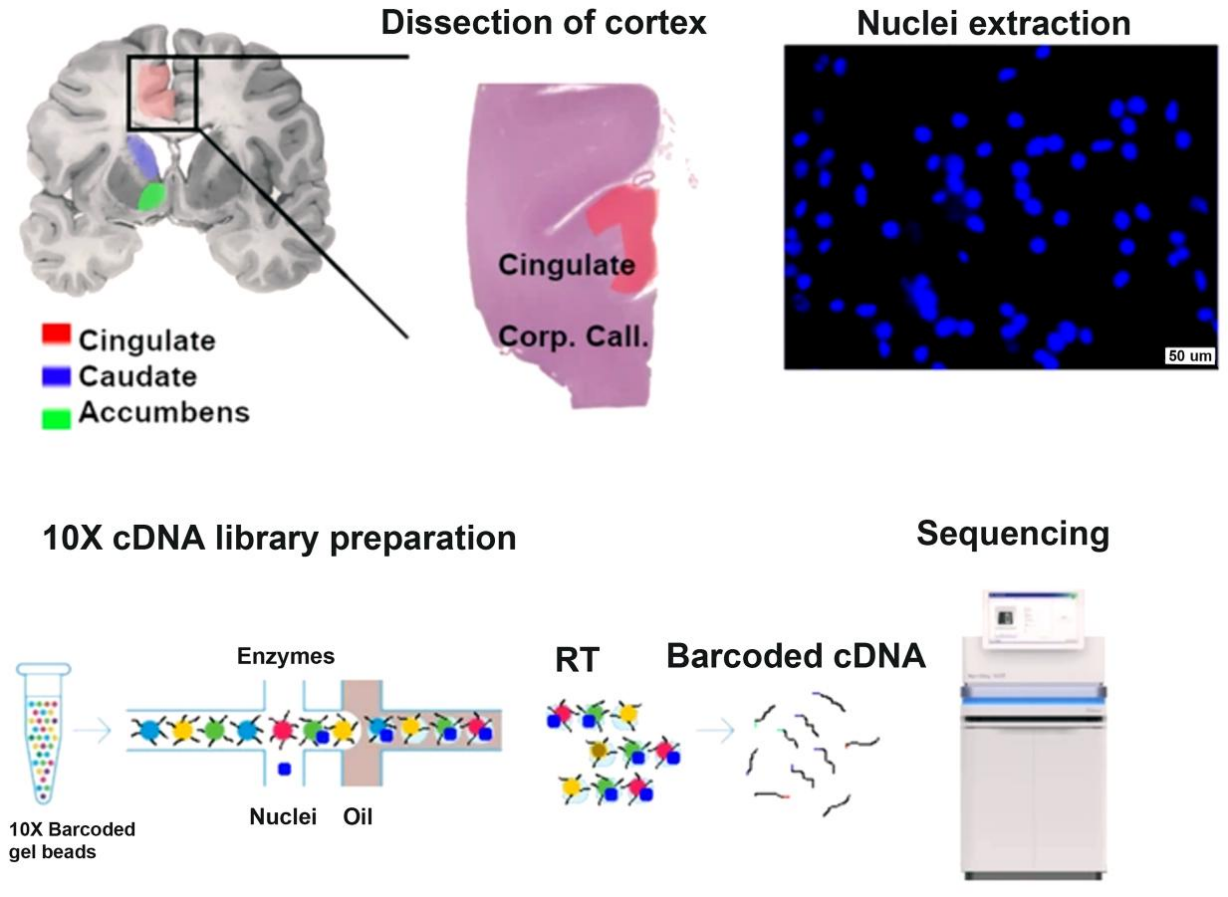
**Validation using snRNAseq of the cingulate cortex in control and Huntington’s disease.** (**A**) Experimental scheme from Al-dalahmah *et al.*^72^. First, cingulate cortex was dissected, nuclei were extracted and visualized using DAPI nuclear stain under a fluorescence microscope to ascertain membrane integrity. The nuclei were subjected to 10× chromium scRNAseq workflow involving encapsulation of nuclei in oil droplets along with enzymes and barcoded beads, followed by cDNA synthesis and library preparation, and finally, sequencing. (Reproduced from Al-Dalahmah *et al*.^72^ under the terms of the Creative Commons CC BY licence.) (**B**) *P*-values for analysis testing enrichment of Huntington’s disease striatum and cortex genes from Langfelder *et al*.^69^ and Huntington’s disease cell-specific neuronal, astrocyte and microglia genes from Al-Dalahmah *et al*.^72^

For the partial correlation analysis, genes which showed abnormal transcription in the cortex in human Huntington’s disease and animal models were significantly enriched in the ranked gene list from the PLS1 (*P* < 1 × 10^−10^). However, genes that showed abnormal transcription in the striatum were not significantly enriched (*P* = 0.99; Fig. 4). This suggests that CSF NfL-related increases in functional connectivity are predominantly related to cortical and not striatal Huntington’s disease pathology. Neither striatal (*P* = 0.16) nor cortical genes (*P* = 0.8) were enriched in the ranked gene list from the PLS1 of the Group × NfL analysis. Consistent with the 114 regions of interest partial correlation analysis, the 514 regions of interest partial correlation analysis showed enrichment of cortex genes (PLS1, *P* = 0.003; PLS2, *P* = 2 × 10^−4^) but not striatal genes (PLS1, *P* = 0.98; PLS2, *P* = 0.86).

### Enrichment analysis of cell-specific genes showing abnormal transcription in snRNAseq in Huntington’s disease

For the partial correlation analysis, neuronal (*P* < 1 × 10^−10^) and microglia (*P* = 0.03, uncorrected) genes that showed abnormal transcription in Huntington’s disease post-mortem brains were significantly enriched in the ranked gene list from the PLS1. Astrocyte genes abnormally transcribed in Huntington’s disease were not significantly enriched (*P* = 1), suggesting the cortical pathology associated with CSF NfL functional connectivity increases is associated with neuronal Huntington’s disease-related changes (Fig. 4). Neither neuronal (*P* = 1), astrocytic genes (*P* = 0.87) nor microglia (*P* = 0.97) were enriched in the ranked gene list from the PLS1 of the Group × NfL analysis. Consistent with the 114 regions of interest partial correlation analysis, the 514 regions of interest partial correlation analysis showed enrichment of neuronal genes for PLS2 (*P* < 1 × 10^−10^) but not PLS1 (*P* = 0.09). No significant enrichment was seen for astrocytic or microglia genes in PLS1 1 or PLS2.

## Discussion

We characterized functional brain networks in asymptomatic preHD gene carriers very far from disease onset and related these networks to measures of white matter organization, disease burden and gene expression. Despite there being no differences in functional or structural connectivity comparing controls and preHD participants, we identified a significant positive correlation, predominantly in posterior regions, between functional connectivity and disease burden as measured by CSF NfL, a fluid biomarker of axonal degeneration, detectable in those many years from Huntington’s disease clinical diagnosis. Using data from the AHBA and performing cell-enrichment analysis, we demonstrated that those regions that showed increased functional connectivity were also those with regional expression of genes specific to neuronal GABAergic and glutamatergic cells. This relationship was validated using snRNAseq data from post-mortem Huntington’s disease and healthy control brains, where increased functional connectivity was associated with neuronal genes abnormally transcribed in Huntington’s disease.

Studies have shown that functional connectivity differs between preHD and controls^5,9,10,12^ in cohorts where gene carriers were more advanced, i.e. they showed subtle symptoms and were on average 10–15 years from clinical diagnosis when cognitive changes tend to become evident.^74,75^ Here, in asymptomatic preHD gene carriers, on average 24 years from disease onset with normal cognitive behaviour,^19^ we found no functional connectivity differences when compared to controls, even when constrained by the structural connectome. However, we did identify a positive association between functional connectivity and CSF NfL, indicating that connectivity changes relate to disease pathology burden rather than being characteristic of asymptomatic preHD *per se*.

As there were no differences in white matter organization in our previous analyses^19,20^ and a limited number of white matter connections showing significant negative correlation with CSF NfL (only for the 514 atlas) in this study, this suggests that large-scale functional changes precede those of microstructure in Huntington’s disease gene carriers furthest from disease onset. It is important to note that as a marker of axonal degeneration, CSF NfL increases indicate some degree of underlying molecular change. The limited change in structural connectivity measures suggests that diffusion-weighted measures lack sensitivity at the very earliest stages of Huntington’s disease and changes in these measures can only be detected after a certain threshold of cumulative change at the molecular level. Nevertheless, with currently feasible *in vivo* measures, functional connectivity appears to change prior to structural connectivity.

Our findings are consistent with our earlier work focusing exclusively on fronto-striatal connectivity.^11^ In that earlier work, fronto-striatal connectivity related to cognitive flexibility (posterior regions were not interrogated as part of these analyses) differed in preHD participants, while connections from the striatum to both frontal and posterior cortical regions showed higher connectivity with evidence of compensatory activity to support maintained performance (in review). In the present work, we went beyond our earlier study to now identify positive associations between CSF NfL and functional connectivity in posterior cortical regions. This is of particular interest, given that in our previous work we demonstrated a clear anterior–posterior gradient of functional connectivity upregulation.^12^ However, this was in gene carriers 10–15 years from clinical diagnosis. Thus, one possibility is that there is a shift in compensatory functional connectivity changes, from posterior to anterior, as pathology becomes more significant in the earliest preHD stages. This should be investigated further in future longitudinal studies.

To understand the basis of the NfL-related increases in functional connectivity that we found, we investigated how brain areas where functional connectivity increased might relate to regional gene expression determined from the AHBA. GO showed an association with biological processes involving synaptic transmission, while EWCE analysis indicated specificity to GABAergic and glutamatergic neuronal cells, which was further supported using independent snRNAseq data from post-Huntington’s disease and healthy control brains. There is significant evidence to suggest that upregulated functional connectivity in neurodegeneration is associated with both glutamate excitotoxicity from pyramidal cells^76,77^ and loss of GABAergic inhibition from interneurons in both mouse models^78^ and human cells,^79,80^ which seems to be located within the cortex rather than the striatum.^22,23,78–82^ Furthermore, there is a dissociation in terms of the way in which degeneration of cortical interneurons relates to the main presenting symptom in Huntington’s disease. Reduced interneurons in the anterior cingulate cortex, for example, are associated with a predominant mood phenotype, while the primary motor cortex is associated with a motor phenotype.^79–81^ Interestingly, genes showing abnormal transcription in Huntington’s disease cortex were enriched in our analysis, while those showing abnormal transcription in the striatum were not. These findings, however, must be considered with the caveat that the AHBA gene expression data reflect gene expression determined in participants without any neurological disease; post-mortem brain data for Huntington’s disease gene expansion carriers very far from onset are not currently available.

Gene enrichment results for upweighted genes in the partial correlation analysis were similar to downweighted genes in the NfL × Group interaction analysis. While the direction of effect in the partial correlation analysis is intuitive, such that a positive correlation indicates higher functional connectivity is associated with higher NfL, the interpretation of the NfL × Group interaction is more difficult. Furthermore, the absence of a relationship between NfL and functional connectivity in the control group suggests the possibility that the inclusion of the control group in the model could introduce noise and reduce the pathobiological signal of the preHD NfL relationship. Indeed, this may explain the borderline significance for the PLS1 and absence of enrichment for any gene set in the NfL × Group interaction analysis. This is in contrast to the partial correlation analyses for both 114 and 514 regions of interest, which showed significance for PLS components and enrichment for cortical and neuronal gene sets.

There are some limitations to the current study. There are no gene expression post-mortem brain data in far from onset premanifest Huntington’s disease gene carriers currently available. Here we show that the spatial distribution of NfL–functional connectivity correlations are associated with neuronal genes implicated in Huntington’s disease pathogenesis. The manifest Huntington’s disease post-mortem data are used to demonstrate that neuronal genes that show differential expression in post-mortem Huntington’s disease brains relative to controls are enriched in the ranked gene list from our PLS analysis. However, we postulate that while the underlying pathobiology of Huntington’s disease remains consistent across the lifetime of the disease, how this emerges at the brain network levels differs across the disease spectrum; for example, while functional connectivity in far-from-onset gene carriers may increase in the context of increasing disease burden, this may then reduce once a critical level of pathology is reached, such that hyperexcitability or compensatory mechanisms become overwhelmed. This is consistent with our previous work.^12,14^

The cohort of 64 preHD gene expansion carriers and 67 controls described here is limited when compared to the larger Track-HD and Predict-HD studies. However, recruiting preHD gene-expansion carriers very far from onset is challenging for a number of reasons. The uptake of genetic testing in this age group is much lower than in those closer to onset^83^ and this group is much less likely to attend Huntington’s disease clinics regularly, if at all, when compared to preHD gene carriers within 10 years from onset. Tattoos were more common in this age group and both tattoo location and size could result in exclusion from MRI scanning. Finally, this study required participants to agree to undergo lumbar puncture, an invasive procedure.

While there is no clear optimal atlas for connectomics^84^ we selected the Schaefer cortical resting-state fMRI atlas, which is based on 1489 healthy participants and provides parcellation schemes ranging from 100 to 1000 nodes. We performed NBS and genetic analyses both on the 100 and 500 parcellations to replicate our findings on coarse- and fine-grained atlases. We opted not to use schemes above 500 nodes as connectome reliability decreases considerably, particularly for diffusion MRI-derived structural connectomes, at denser parcellation schemes.^73^ Both the rsfMRI brain parcellation atlas and the AHBA are derived from the brains of healthy controls. When considering the application of these in our very-far-from-onset preHD gene expansion carrier cohort we must emphasize that detailed multimodal neuroimaging analysis in this cohort has demonstrated that the brain structure is largely normal.^19^ Furthermore, with respect to regional levels of gene expression and the application of the AHBA atlas, to date, transcriptomic changes in human Huntington’s disease have been demonstrated in post-mortem brains, which are typically at the end stage of the disease, or Huntington’s disease rodent models^85^ and have upwards of 100 CAG repeats—more representative of the juvenile Huntington’s disease variant.^86^

This study has characterized functional brain networks in asymptomatic preHD gene carriers very far from disease onset, showing evidence of upregulated functional network connectivity related to disease burden in the presence of normal white matter brain networks. This relationship was found between brain areas that show regional expression of genes specific to neuronal GABAergic and glutamatergic cells following cell-enrichment analysis; a finding that was supported by snRNAseq data from post-mortem Huntington’s disease and healthy control brains that showed an association with neuronal genes abnormally transcribed in Huntington’s disease. In sum, those furthest from Huntington’s disease disease onset display pathology-related functional connectivity changes that are likely characterized by GABAergic inhibition and glutamatergic excitotoxicity.

## Supplementary Material

awac227_Supplementary_Data
