# Supplementary material for: Neurofilament light-associated connectivity in young-adult Huntington’s disease is related to neuronal genes
Source: Brain. 2022 Jun 27;145(11):3953–67. doi: 10.1093/brain/awac227 (PMC9679168; doi:10.1093/brain/awac227)

# S1

DWI preHD NfL negative correlations
(514 ROIs)

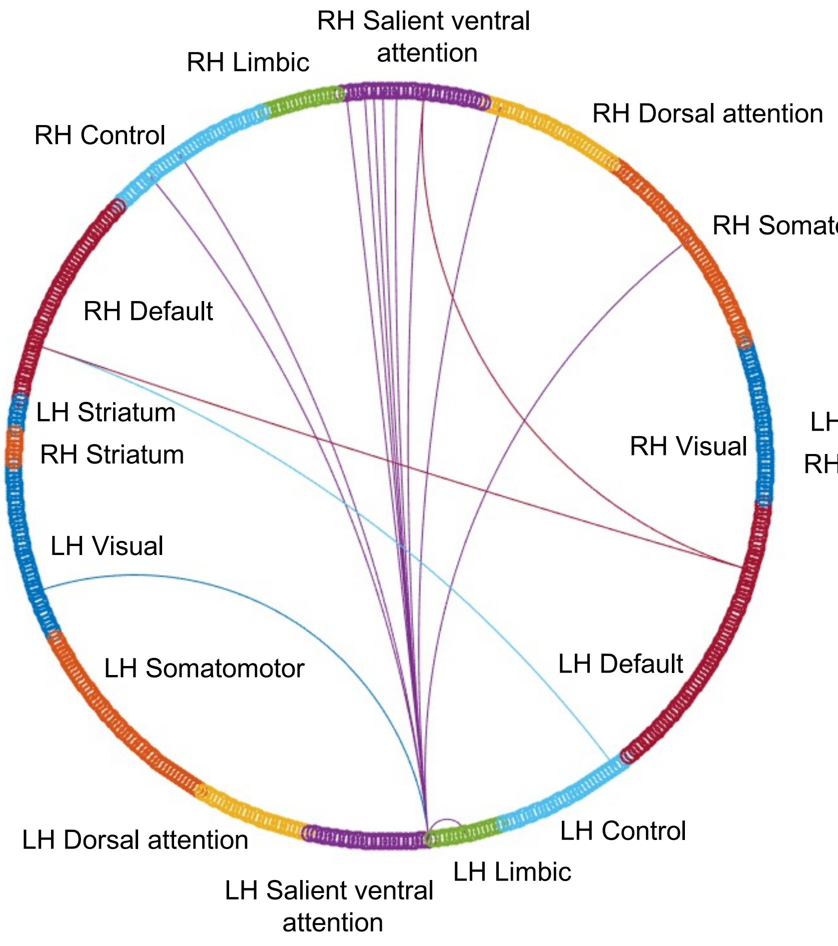


fMRI preHD NfL positive correlations
(514 ROIs)

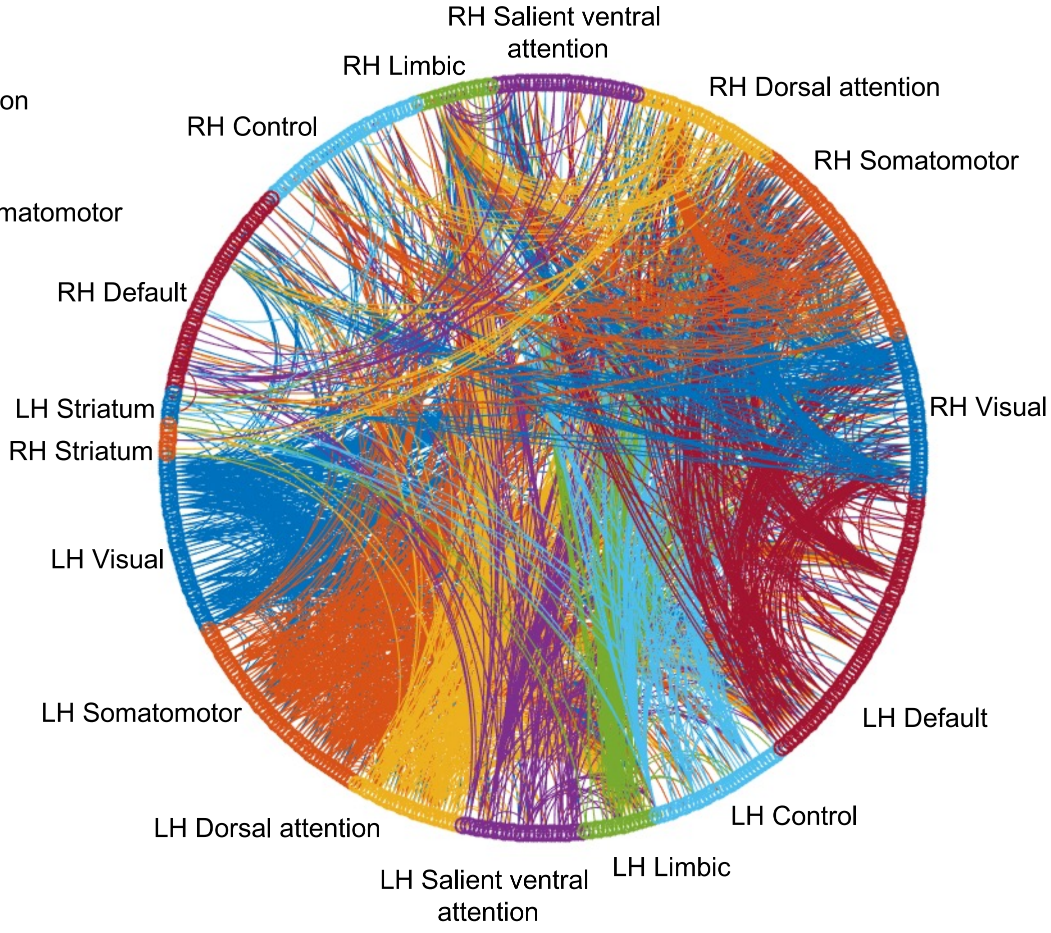

# S2

DWI All NfL negative correlations (514 ROIs)

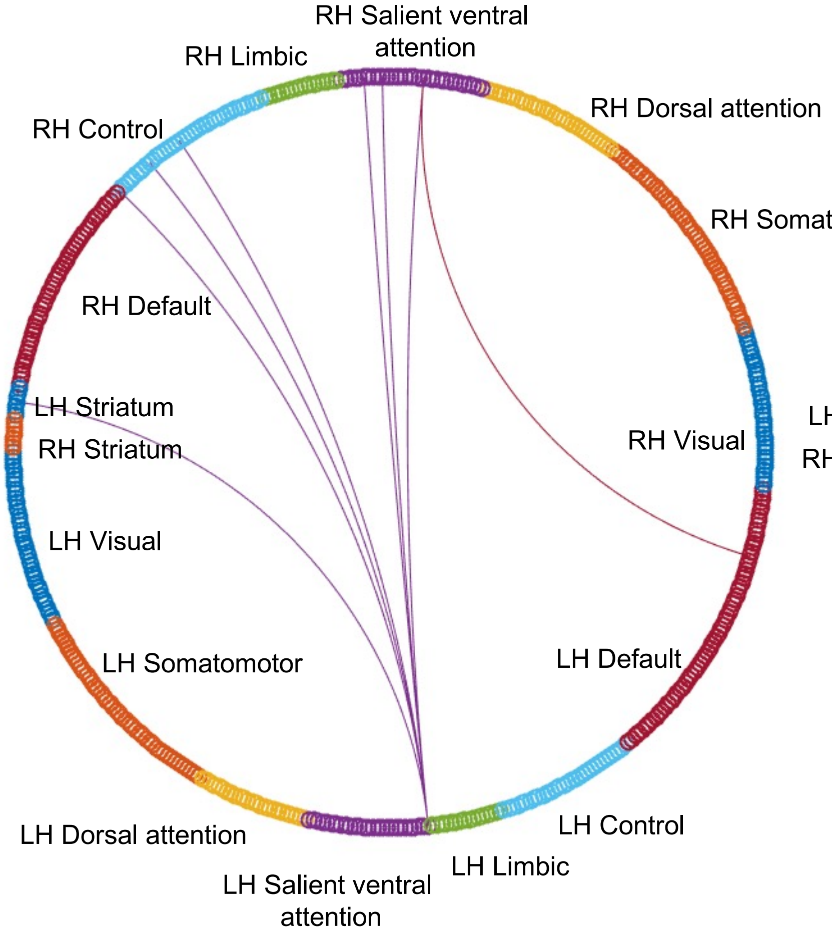


fMRI All NfL positive correlations (514 ROIs)

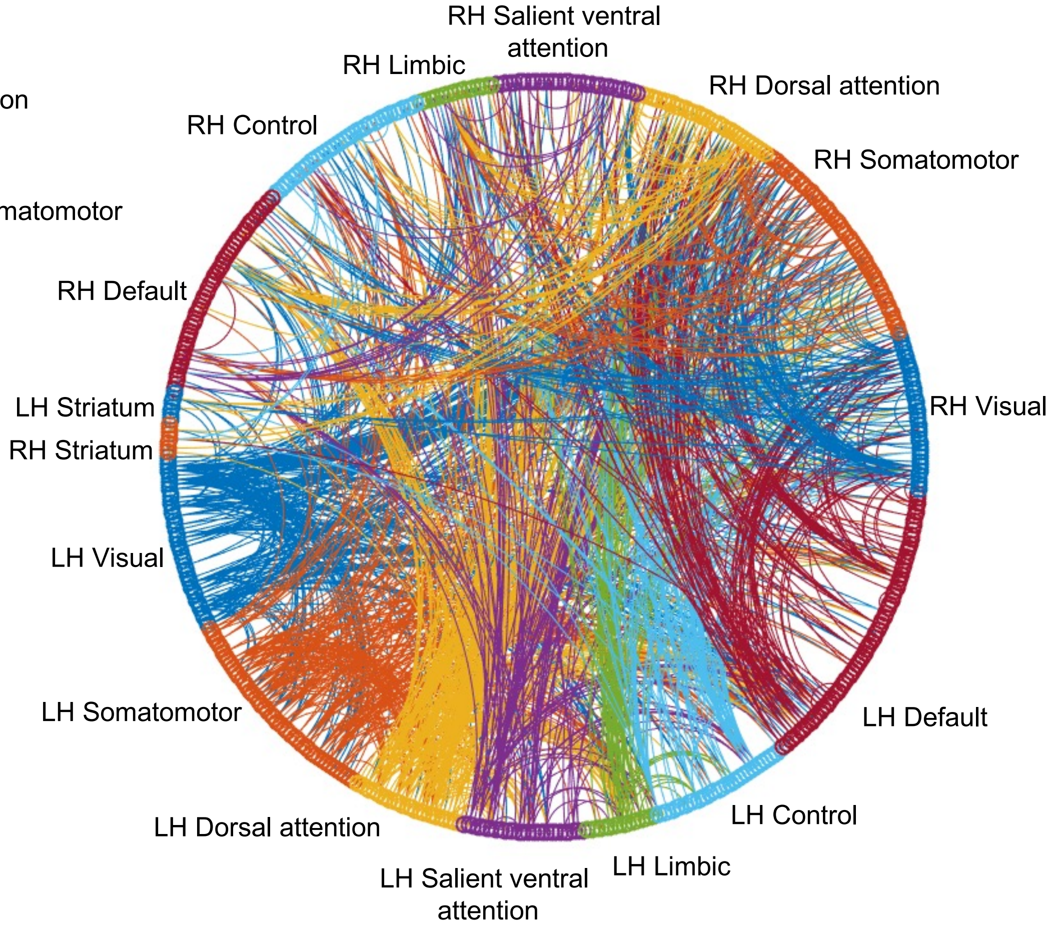

# S3

Partial correlation analysis – Positive Rho values

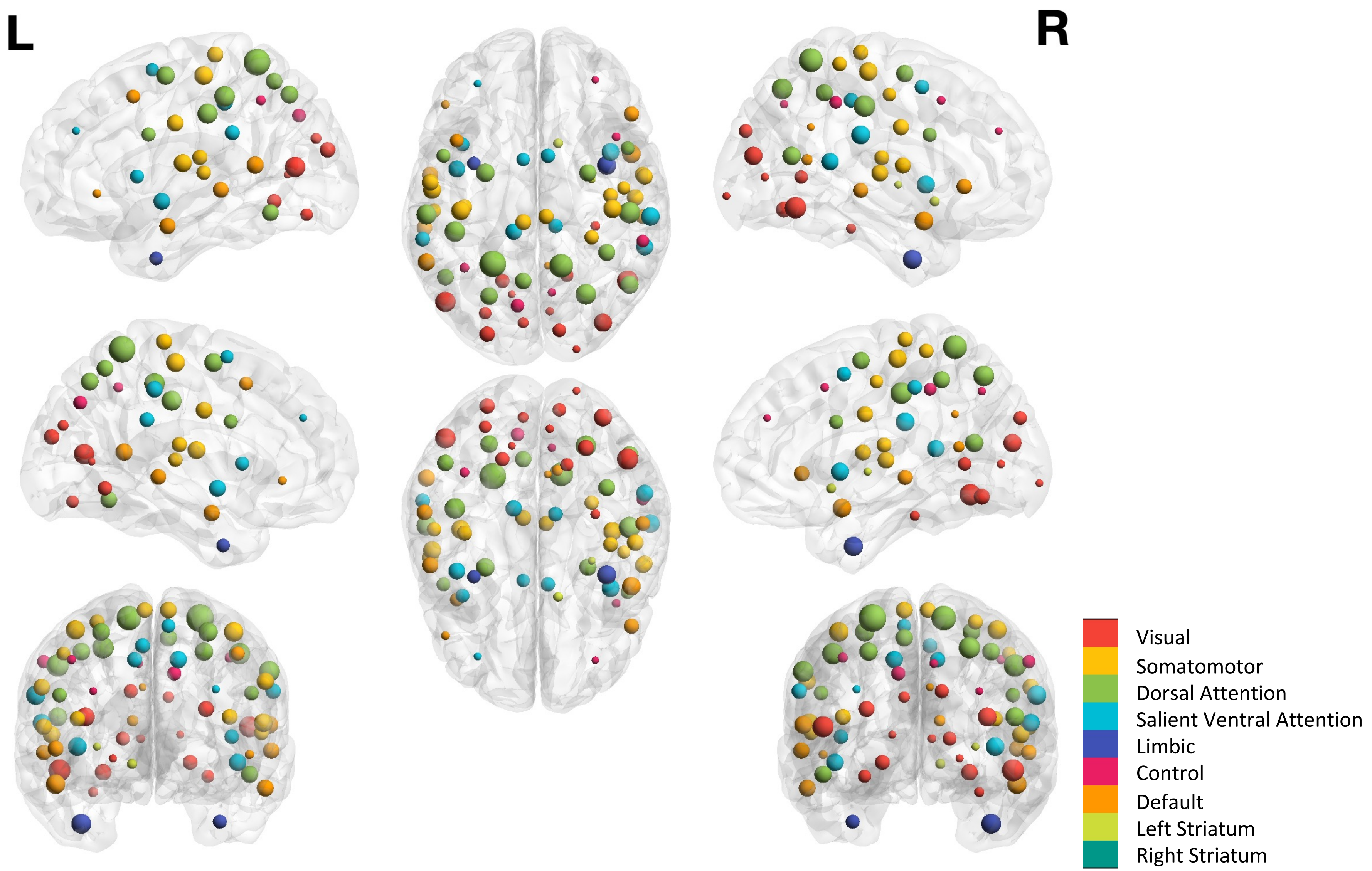

# S4

## Partial correlation analysis – Negative Rho values

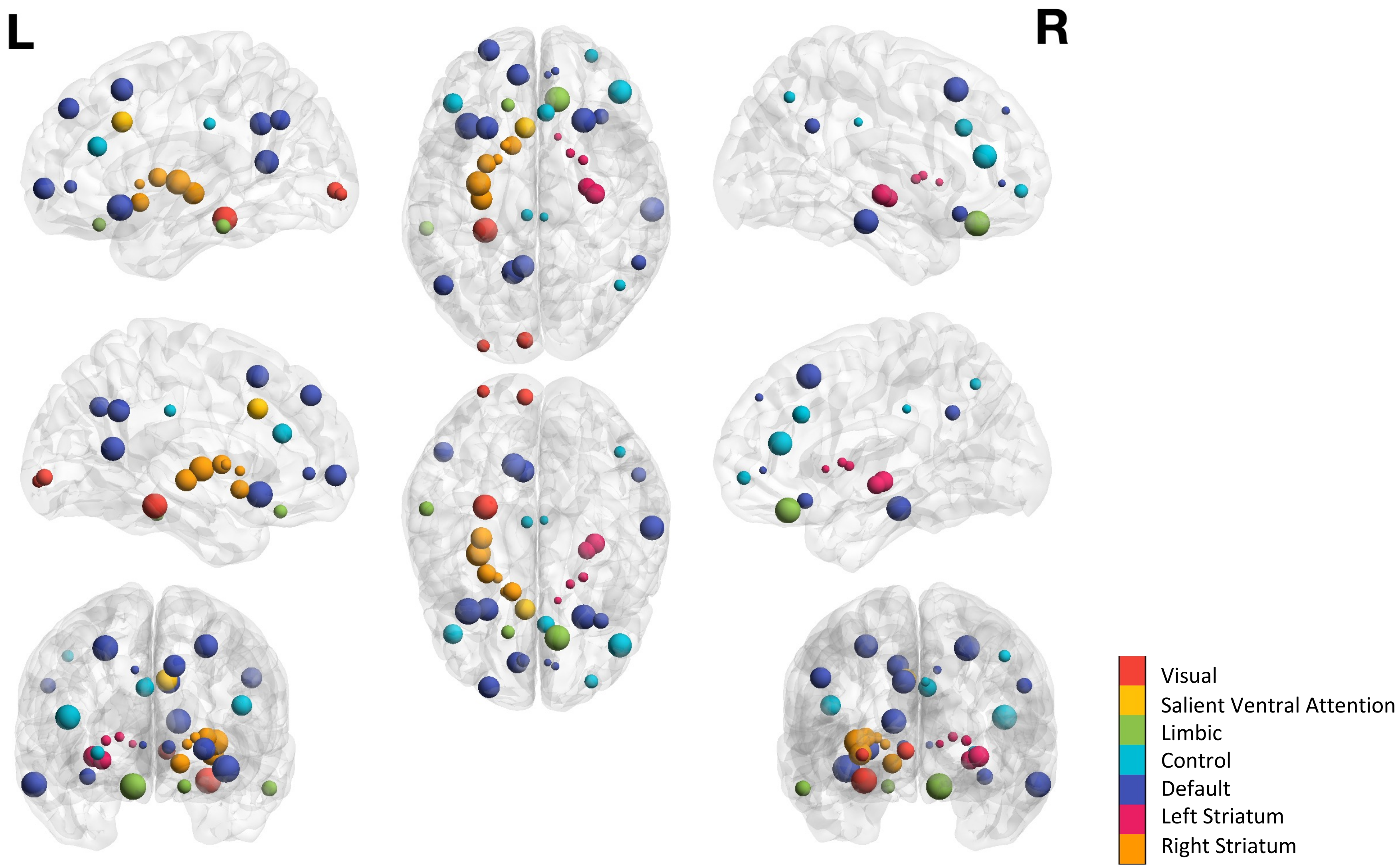

**S5**

Partial correlation analysis – All Rho values (surface visualisation)

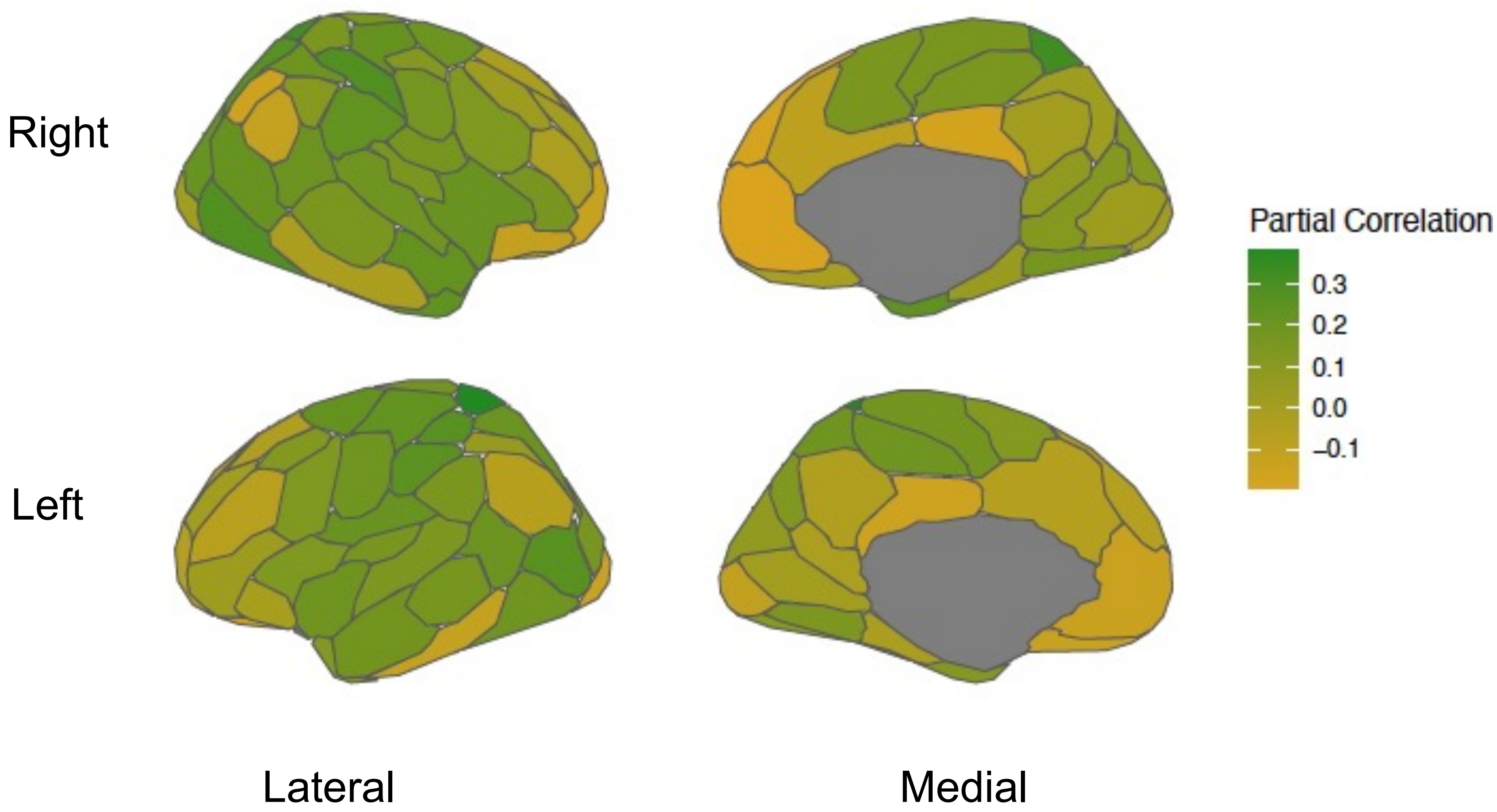

**S6**

Partial correlation analysis – PLS Component 1 Positive ROI weights

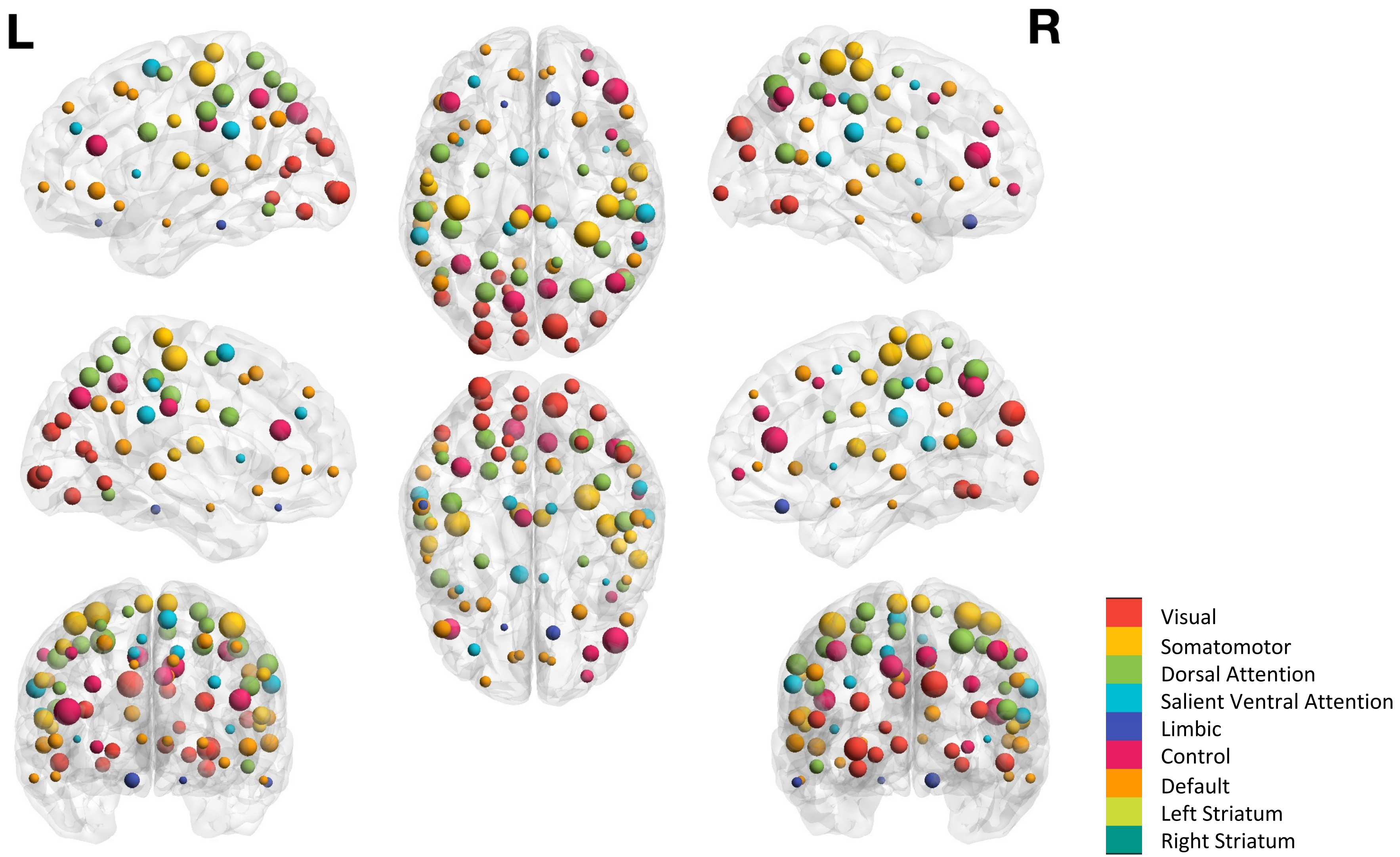

# S7

Partial correlation analysis – PLS component 1 Negative ROI weights

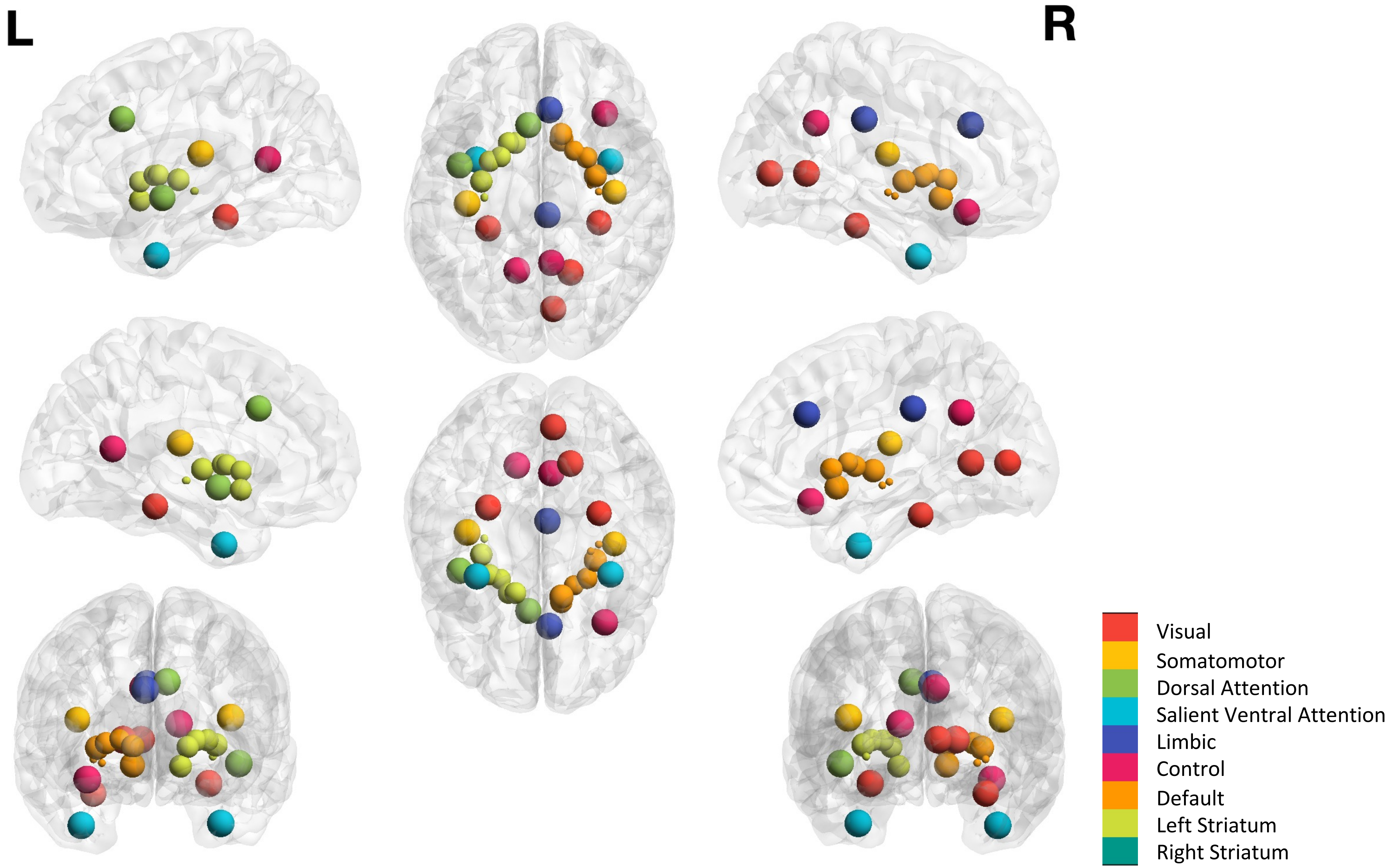

**S8**

Partial correlation analysis – PLS component 1 all ROI weights (scaled) (surface visualisation)

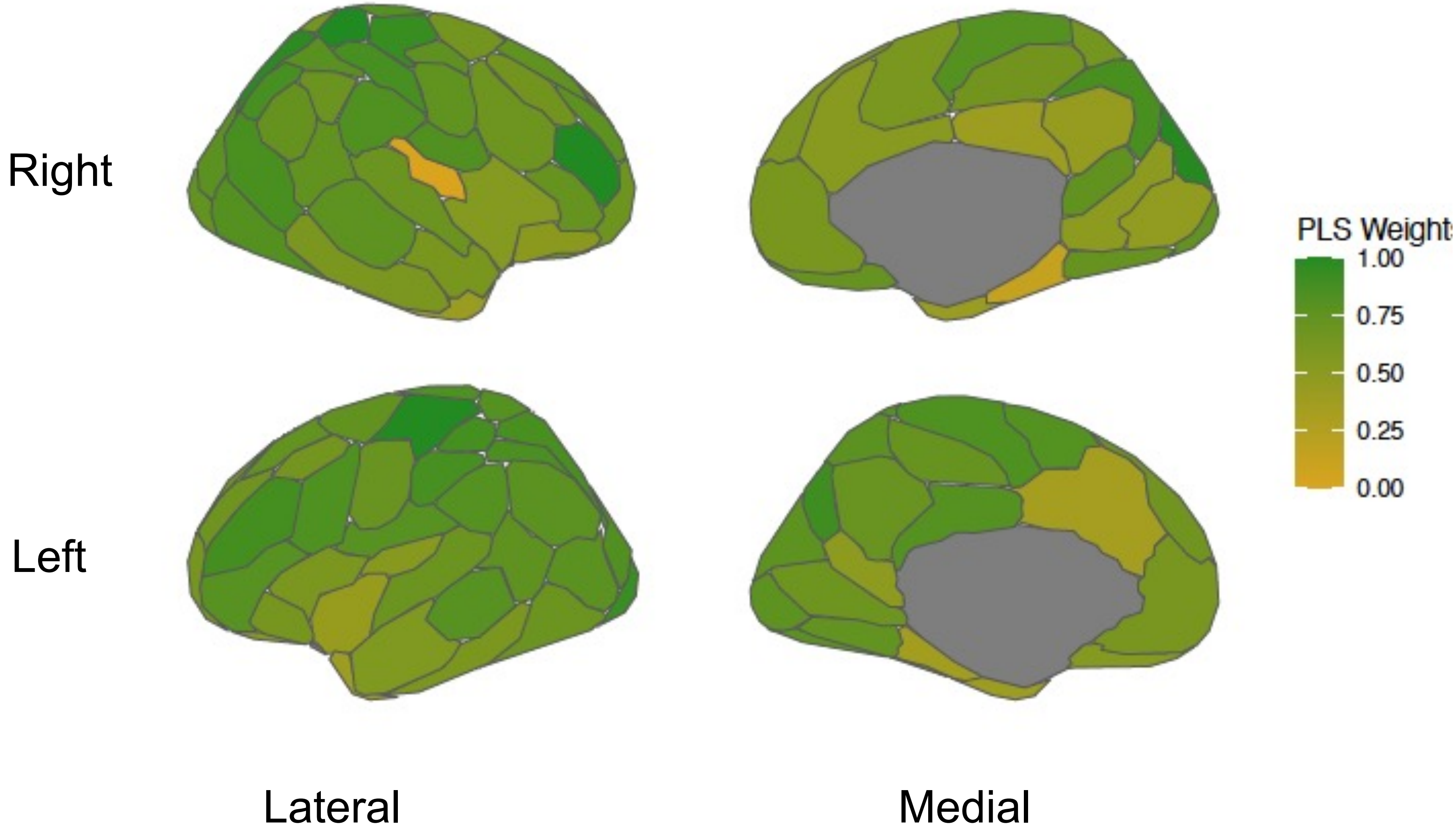

# S9

## Group*NfL analysis – Positive model estimates

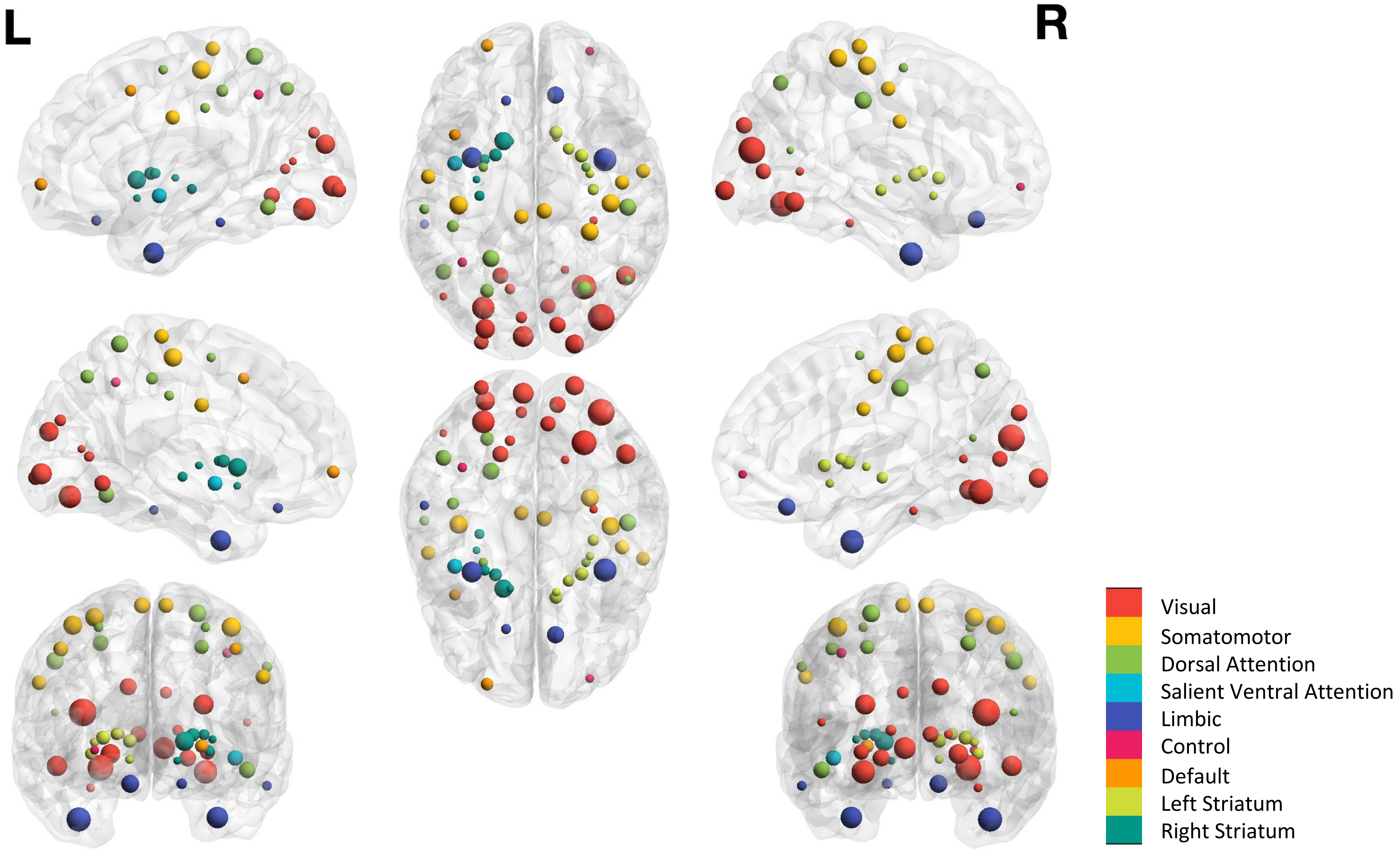

# S10

Group*NfL analysis – Negative model estimates

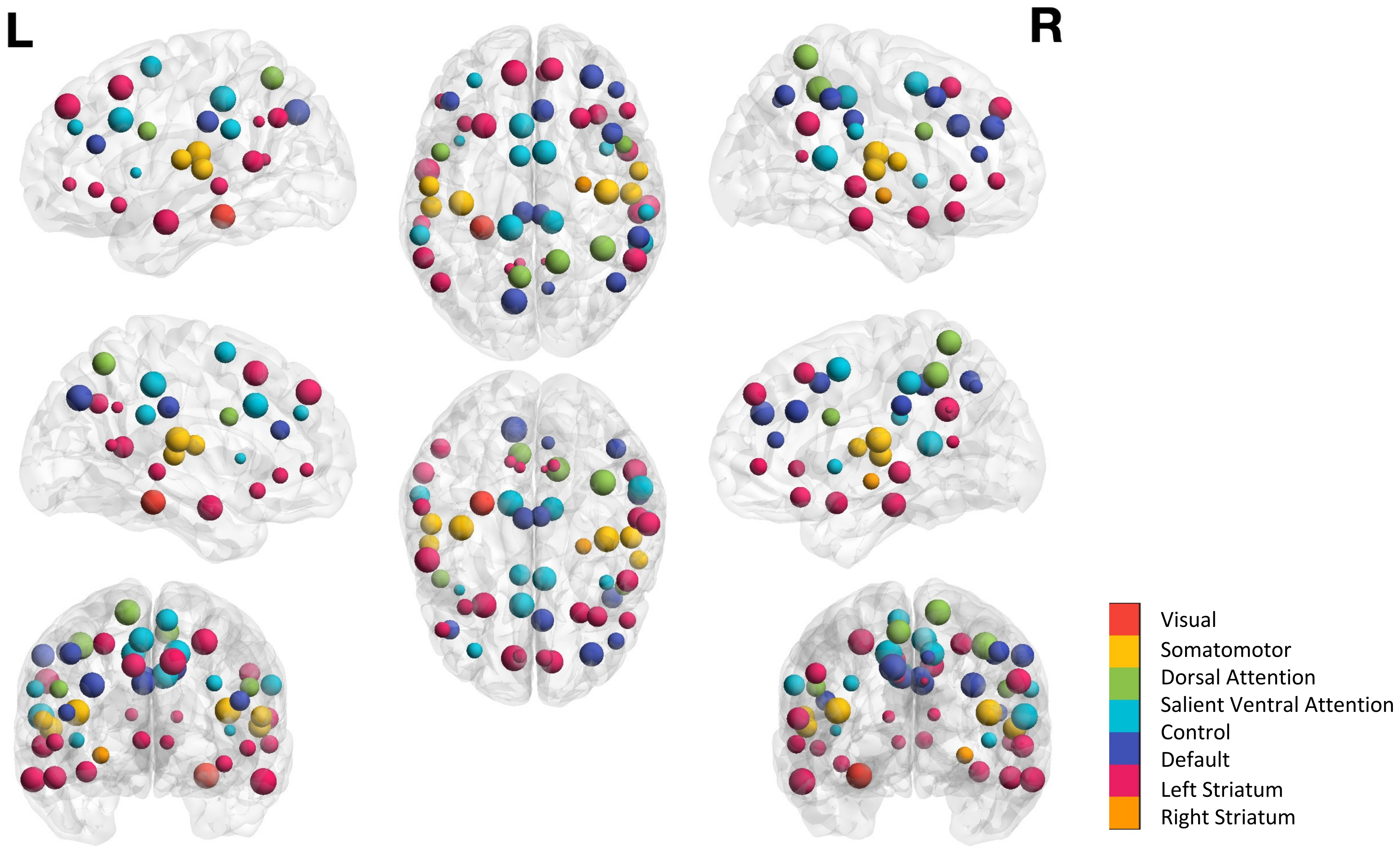

# S11

Group*NfL analysis – All model estimates (surface visualisation)

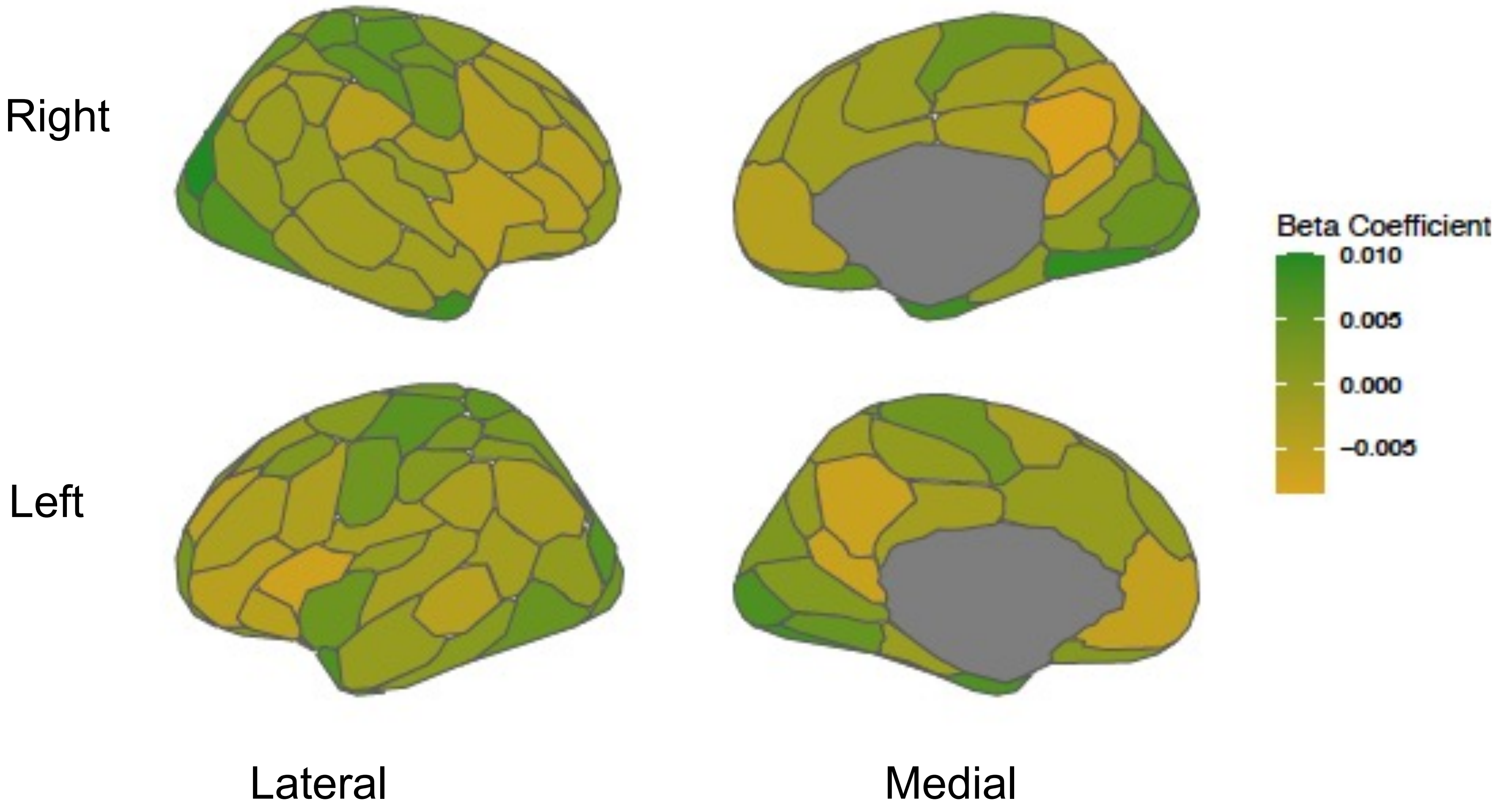

**S12**

Group*NfL analysis – PLS component 1 Positive ROI weights

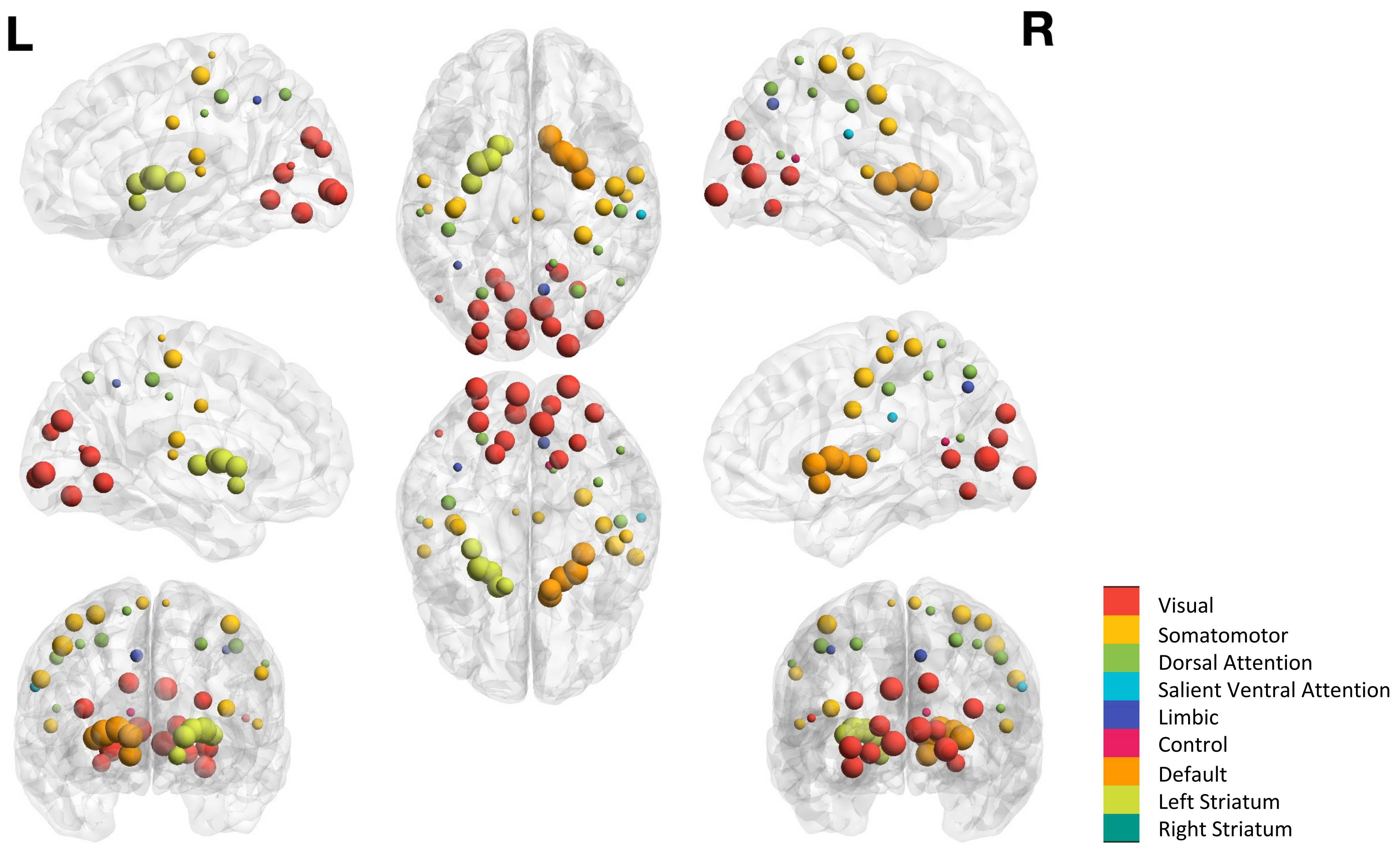

# S13

Group*NfL analysis – PLS component 1 Negative ROI weights

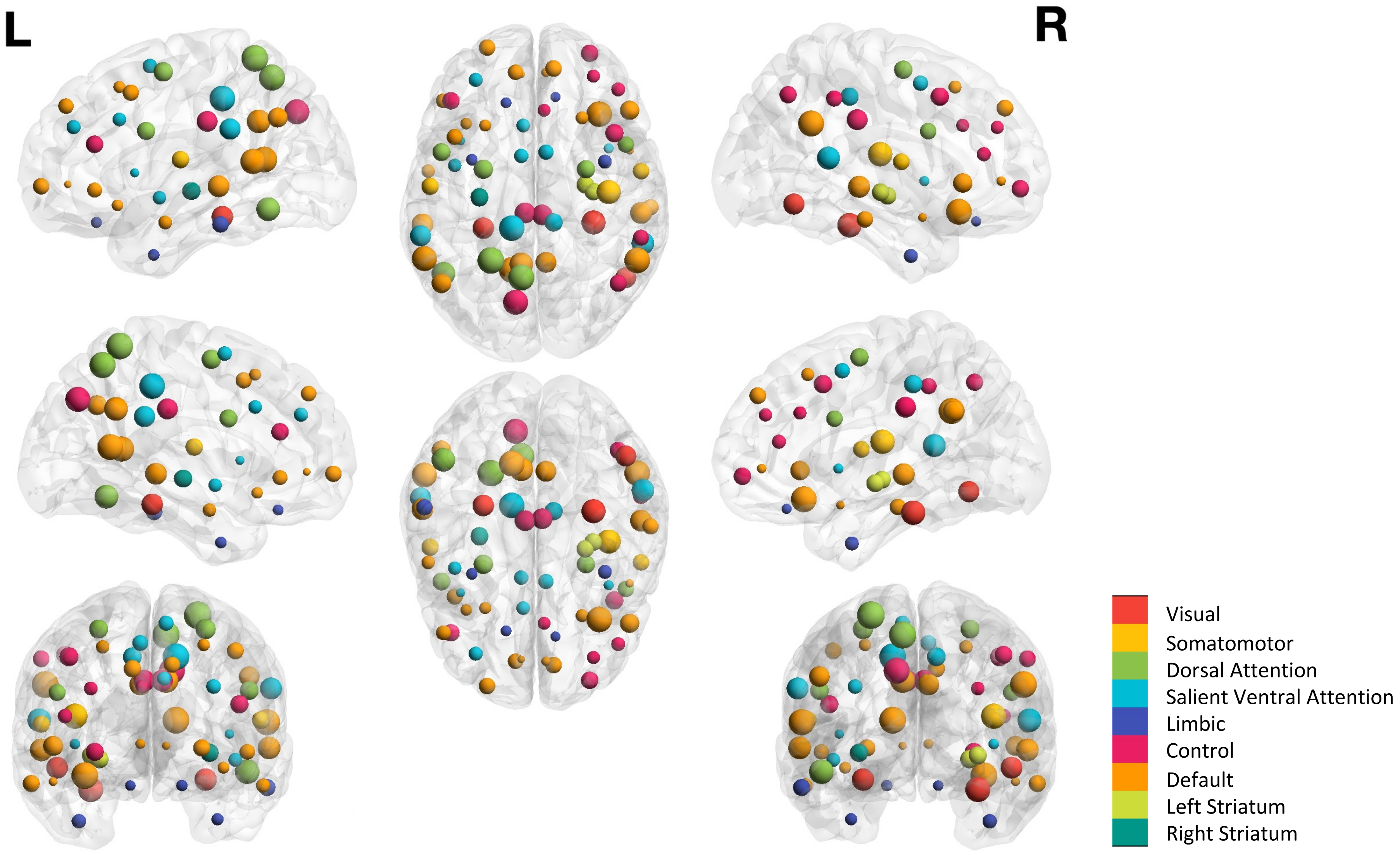

**S14**

Group*NfL analysis – PLS component 1 All ROI weights (scaled) (surface visualisation)

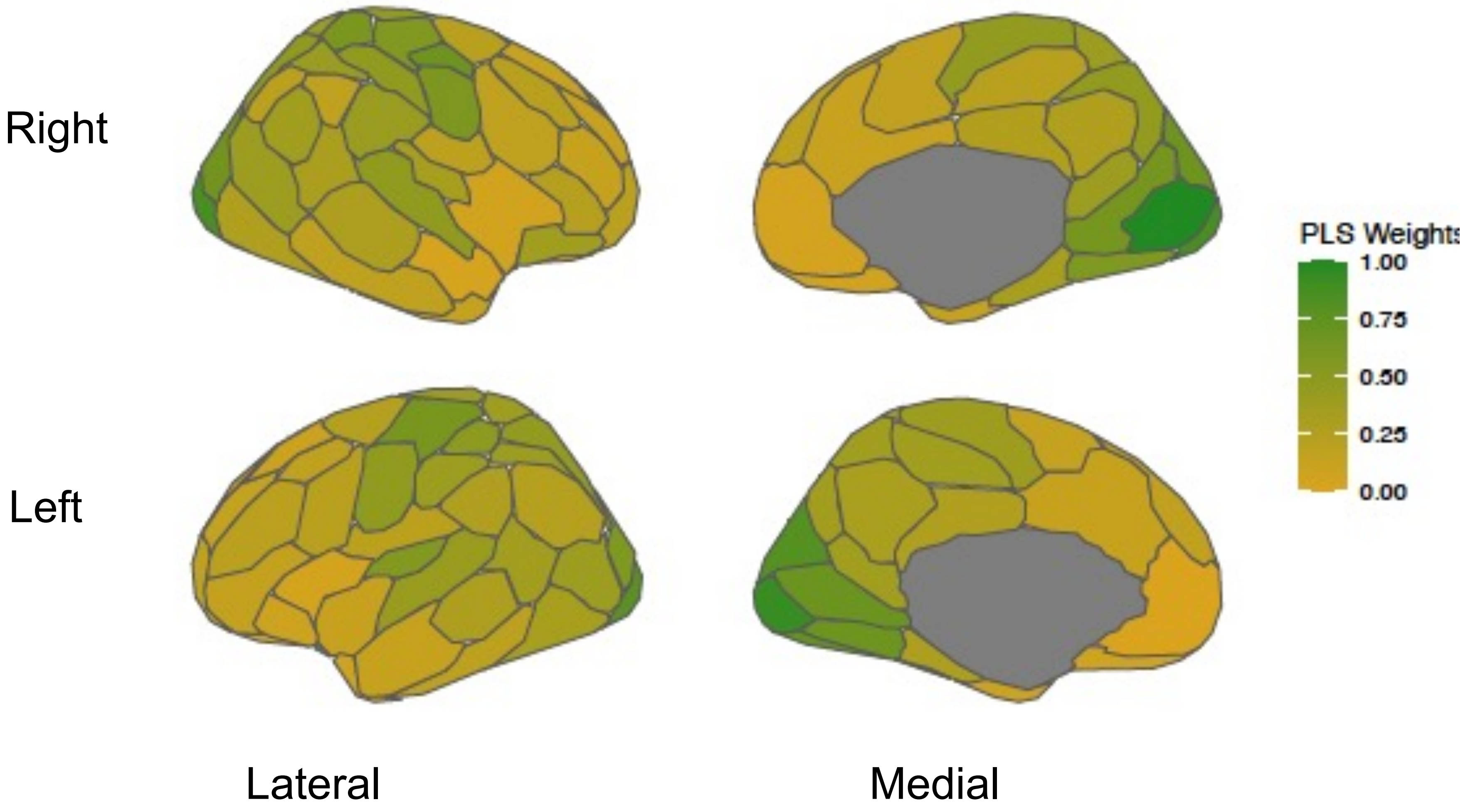

**S15**

Partial correlation analysis (500 ROIs) – All Rho values (surface visualisation)

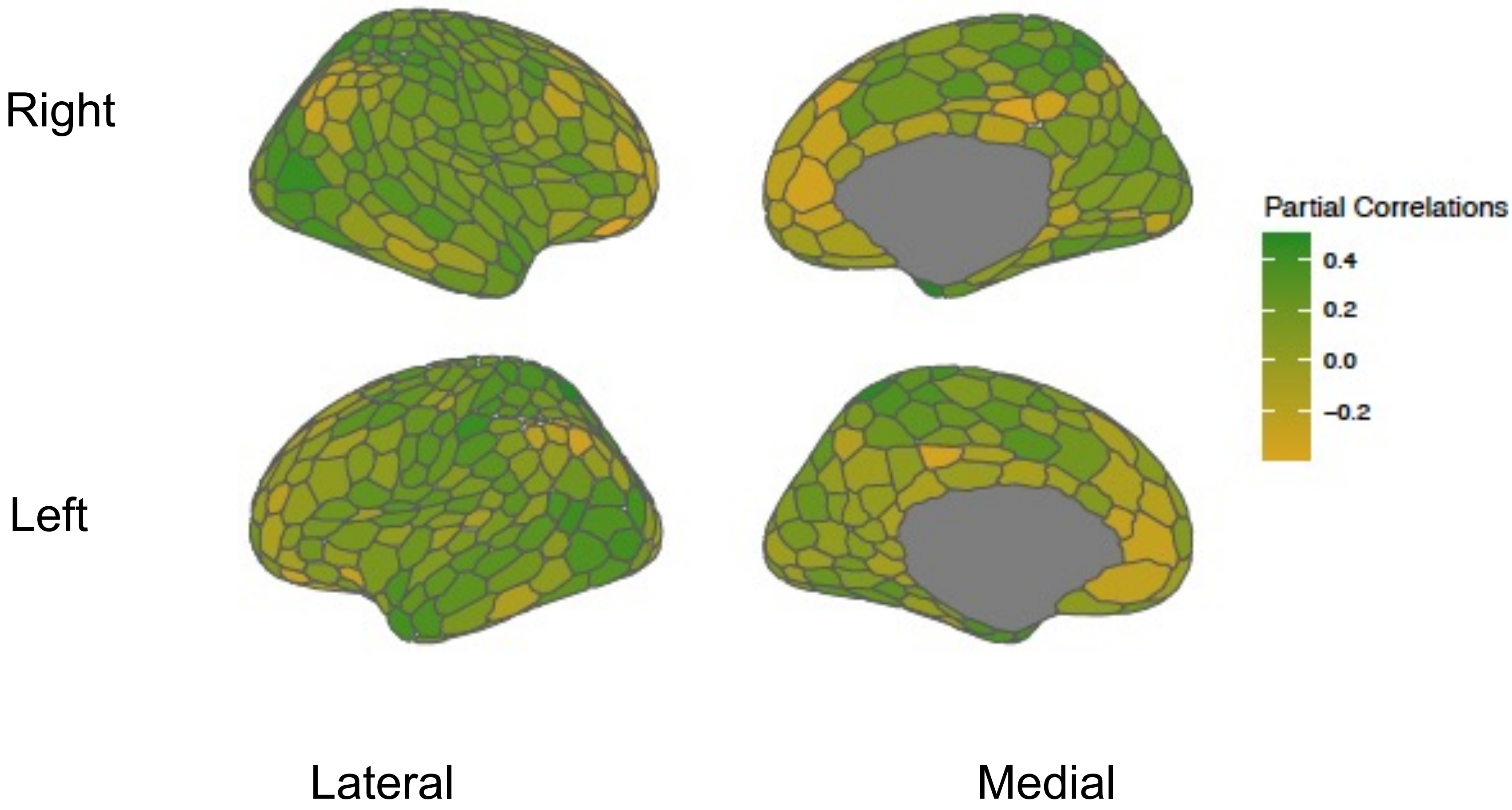

**S16**

Partial correlation analysis (500 ROIs) – PLS component 1 all weights (scaled) (surface visualisation)

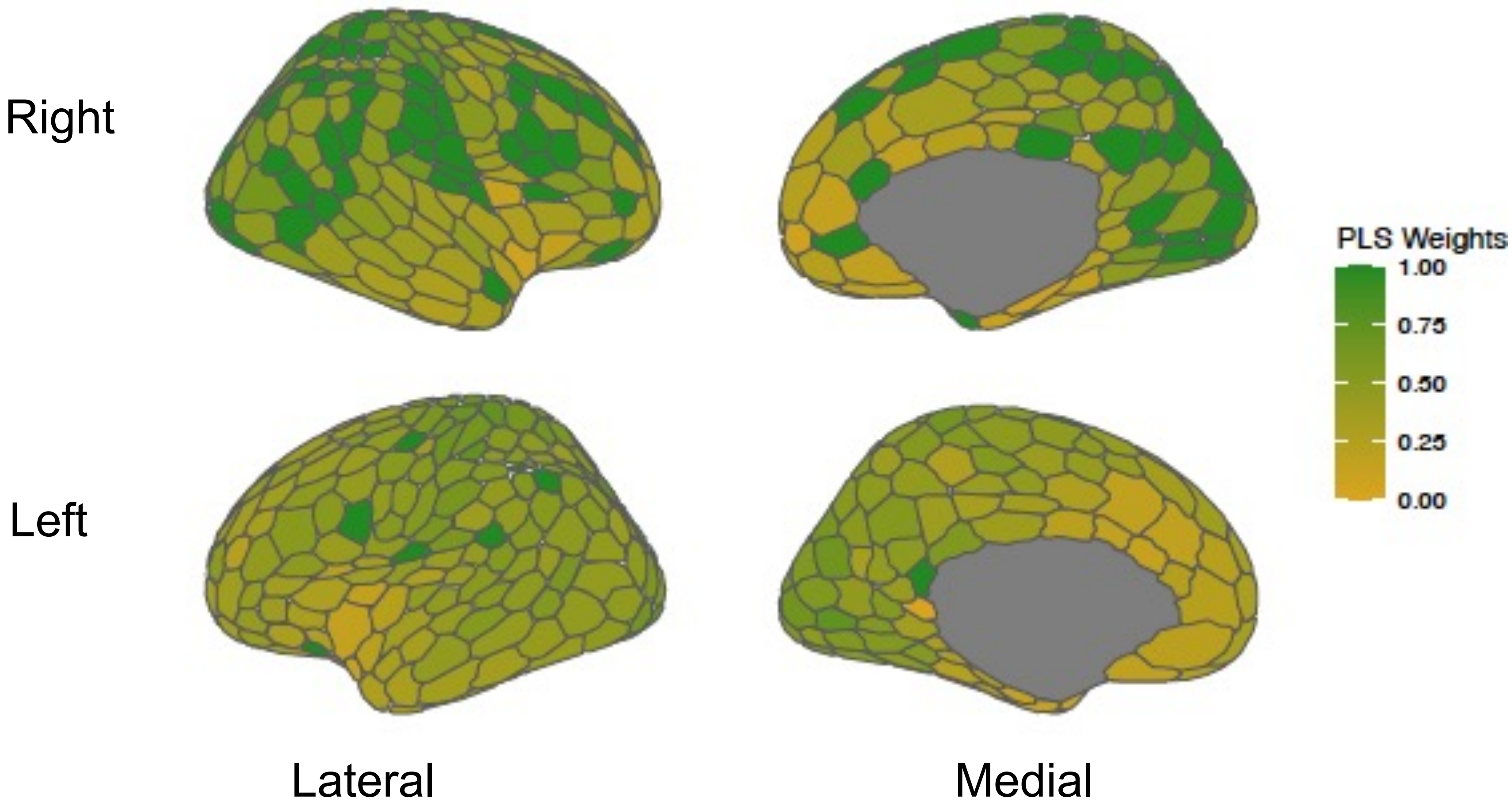

# S17

Partial correlation analysis (500 ROIs) – PLS component 2 all weights (scaled) (surface visualisation)

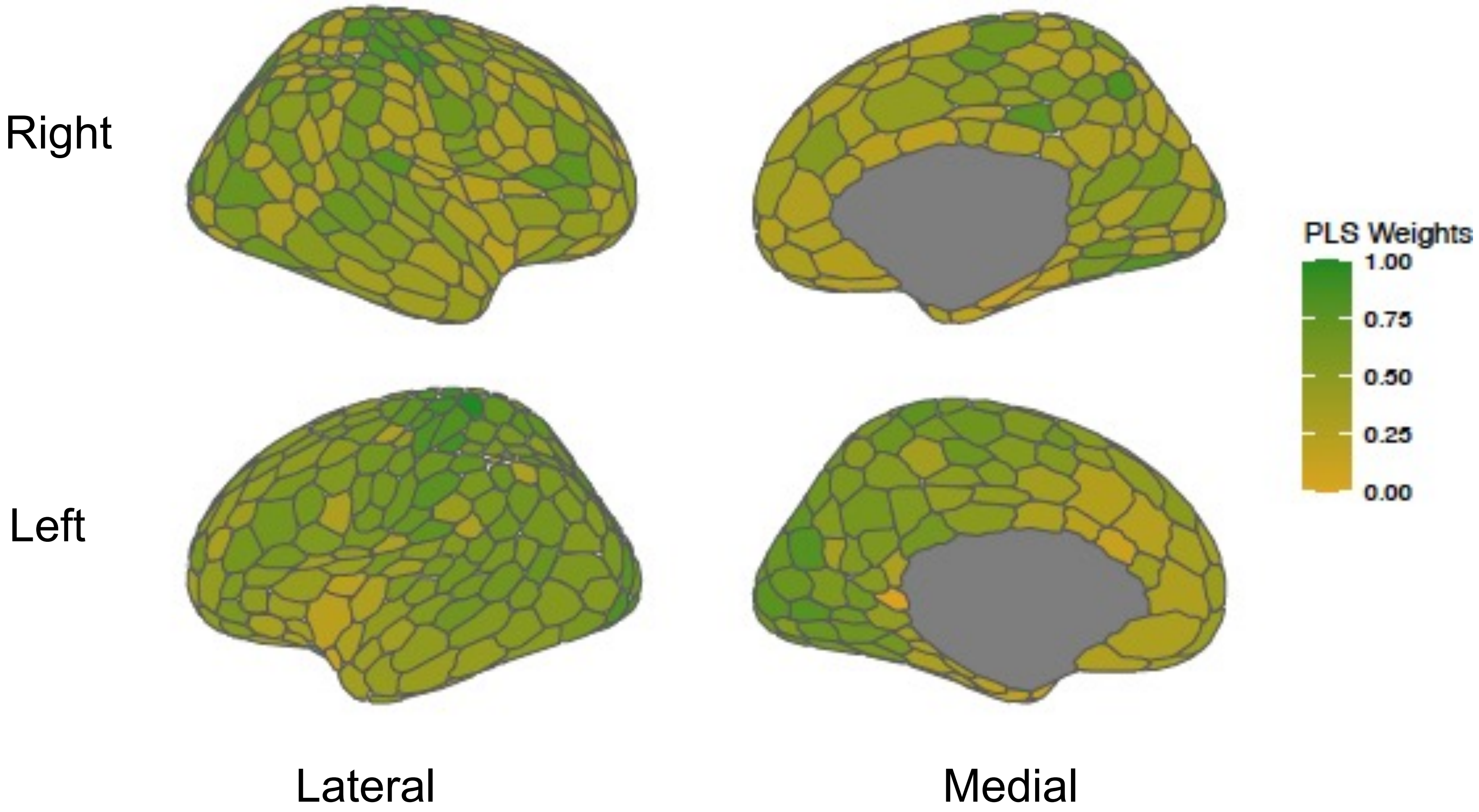

# S18

## Spatial analysis of PLS weights:
## Spearman rank correlation for NfL partial correlation
## 100 region of interest (ROI) analysis

R – rho spearman rank correlation coefficient, p – p value

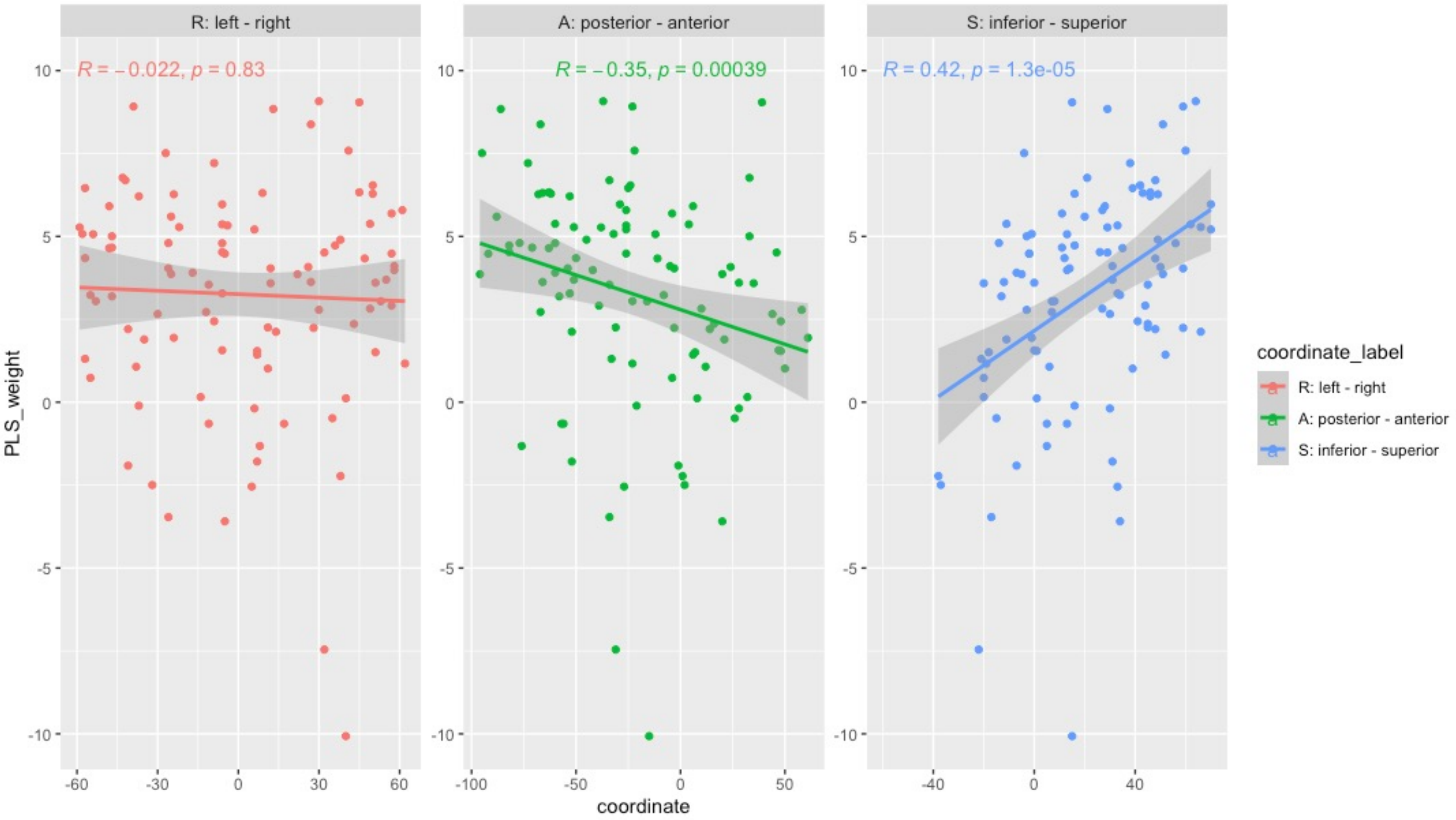

# S19

**Spatial analysis of PLS weights:**
**Spearman rank correlation for NfL partial correlation 500 (component 1) region of interest (ROI) analysis**

R – rho spearman rank correlation coefficient, p – p value

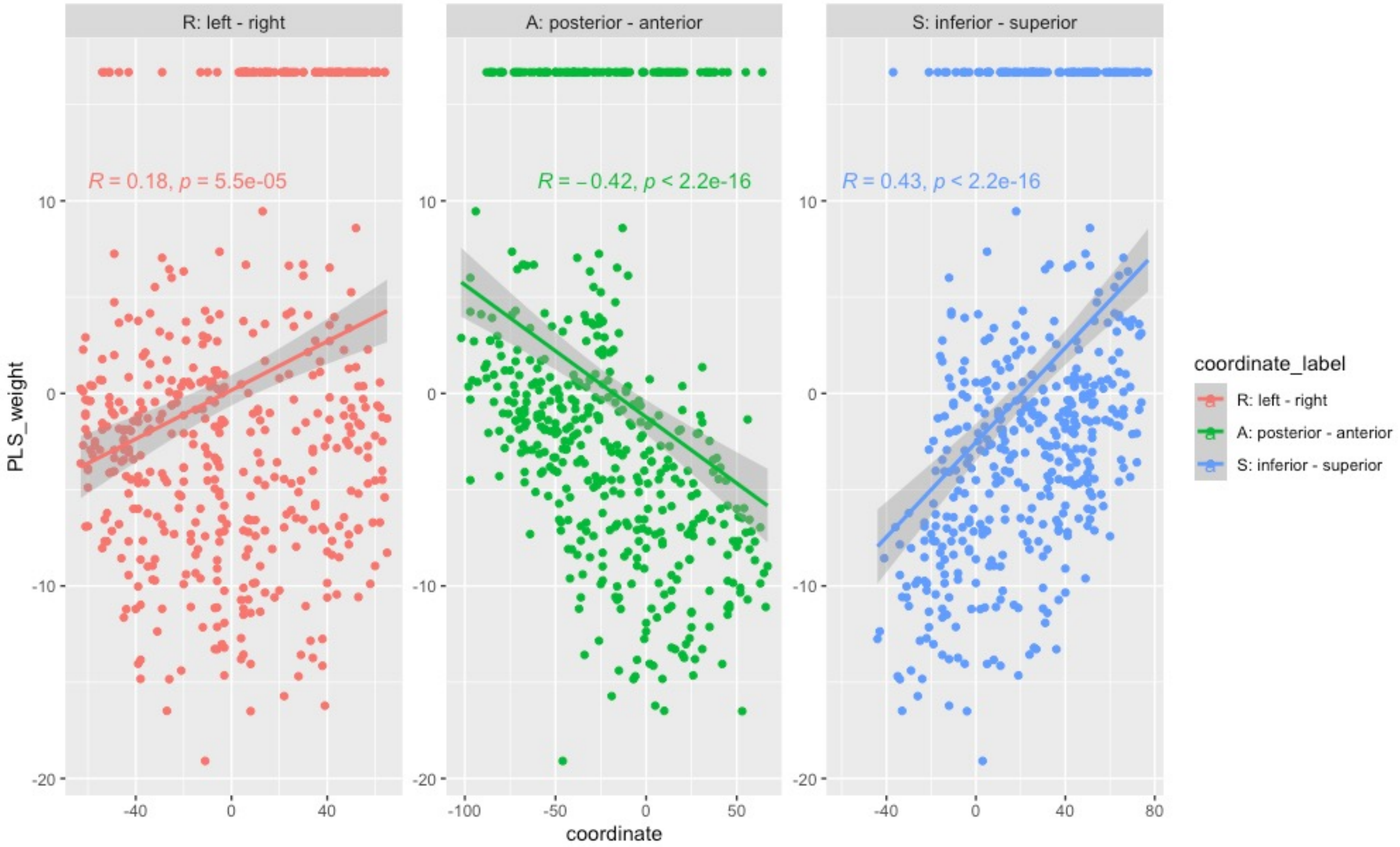

# S20

## Spatial analysis of PLS weights:
## Spearman rank correlation for NfL partial correlation
## 500 (component 2) region of interest (ROI) analysis

R – rho spearman rank correlation coefficient, p – p value

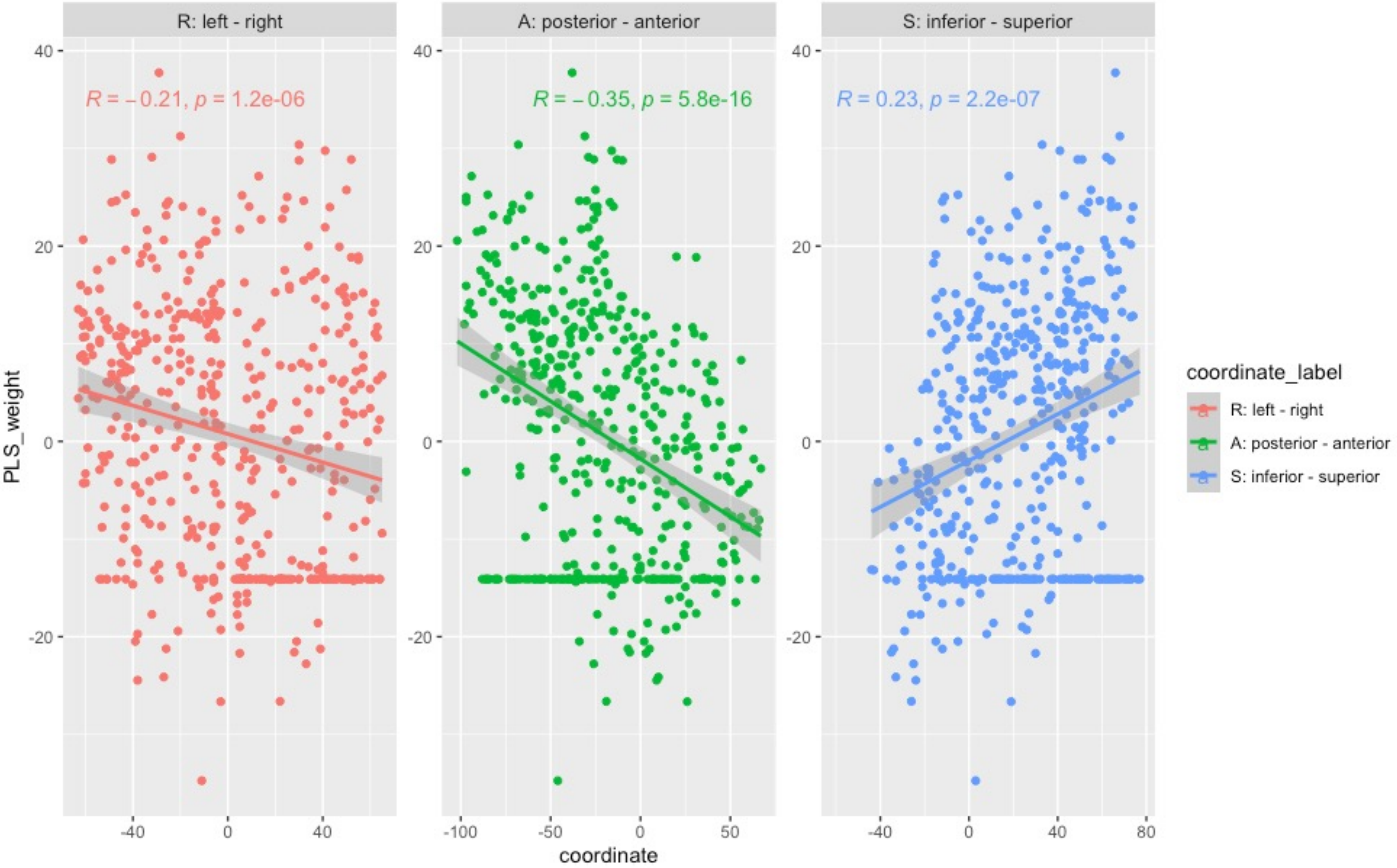

Supplement: awac227_Supplementary_Data [file awac227_supplementary_data.zip › brain-2021-01134-File010.pdf]
