# Supplementary material for: Neurofilament light-associated connectivity in young-adult Huntington’s disease is related to neuronal genes
Source: Brain. 2022 Jun 27;145(11):3953–67. doi: 10.1093/brain/awac227 (PMC9679168; doi:10.1093/brain/awac227)
Supplement: awac227_Supplementary_Data [file awac227_supplementary_data.zip › brain-2021-01134-File011.pdf]

Supplmentary table 1: NBS Summary, (*p<0.05)

**Group t-test**	**PreHD<Cont**	**Cont<PreHD**
Functional (114 ROIs)	0.672	0.6046
Structural (114 ROIs)	0.3447	0.2384
Functional (structural constrained) 114 ROIs	0.6072	0.5792
Functional (514 ROIs)	0.4296	0.5976
Structural (514 ROIs)	0.1504	0.4613
Functional (structural constrained) 514 ROIs	0.2603	0.7998
**ANOVA**		
Functional (114 ROIs)	0.9748	
Structural (114 ROIs)	0.2478	
Functional (514 ROIs)	0.9375	
Structural (514 ROIs)	0.2823	
**Sub-Group t-test**	**low NfL vs. high NfL**	**high NfL vs. low NfL**
Functional (114 ROIs)	0.9489	0.4312
Structural (114 ROIs)	0.1163	0.8771
Functional (514 ROIs)	0.8867	0.365
Structural (514 ROIs)	0.2815	0.8078
**Correlations**	**Positive**	**Negative**
NFL - fMRI correlation (whole group) 114 ROIs	0.0304*	1
NFL - fMRI correlation (preHD) 114 ROIs	0.019*	0.6633
NFL - fMRI ("simult" Conn option) correlation (whole group) 114 ROIs	0.0883	0.5283
NFL - fMRI ("simult" Conn option) correlation (preHD) 114 ROIs	0.1435	1
NFL - fMRI correlation (control) 114 ROIs	0.407	1
NFL - structural correlation (whole group) 114 ROIs	0.191	0.391
NFL - structural correlation (preHD) 114 ROIs	0.2523	0.1818
NFL - structural correlation (control) 114 ROIs	0.8416	1
NFL - fMRI correlation (whole group) 514 ROIs	0.0398*	0.6615
NFL - fMRI correlation (preHD) 514 ROIs	0.027*	0.6478
NFL - fMRI ("simult" Conn option) correlation (whole group) 514 ROIs	0.1491	0.8432
NFL - fMRI ("simult" Conn option) correlation (preHD) 514 ROIs	0.0963	0.7676
NFL - fMRI correlation (control) 514 ROIs	0.5091	1
NFL - structural correlation (whole group) 514 ROIs	0.3177	0.0284*
NFL - structural correlation (preHD) 514 ROIs	0.2919	0.023*
NFL - structural correlation (control) 514 ROIs	0.6763	1

Supplementary table 2: NBS Analysis - Significant connections (fMRI 114 ROIs, preHD only, NfL co

Blue - Corticostriatal

Red - Interhemispheric

Black - Intrahemispheric

Connection 1	Connection 2	Test Statistic
7Networks_RH_Cont_PFCl_3	R_Ventral_attention.	4.06
7Networks_LH_SalVentAttn_PFCl_1	L_Dorsa_lattention.	3.95
7Networks_RH_DorsAttn_Post_4	R_Somatomotor.	3.88
7Networks_RH_Cont_pCun_1	R_Ventral_attention.	3.51
7Networks_RH_DorsAttn_Post_4	L_Somatomotor.	3.44
7Networks_RH_DorsAttn_Post_1	L_Somatomotor.	3.42
7Networks_RH_Cont_PFCl_3	L_Dorsal_attention.	3.27
7Networks_RH_DorsAttn_Post_5	R_Frontoparietal.	3.24
7Networks_LH_Vis_7	R_Somatomotor.	3.18
7Networks_RH_DorsAttn_Post_4	R_Ventral_attention.	3.15
7Networks_LH_Default_Temp_1	7Networks_RH_DorsAttn_Post_5.	5.24
7Networks_LH_Cont_pCun_1	7Networks_RH_SomMot_6.	5.13
7Networks_LH_DorsAttn_Post_5	7Networks_RH_Limbic_TempPole_1.	4.9
7Networks_LH_Default_Par_1	7Networks_RH_Vis_2.	4.79
7Networks_LH_Cont_pCun_1	7Networks_RH_SomMot_2.	4.76
7Networks_LH_DorsAttn_FEF_1	7Networks_RH_Limbic_TempPole_1.	4.74
7Networks_LH_Default_PFC_1	7Networks_RH_DorsAttn_Post_5.	4.71
7Networks_LH_Limbic_TempPole_1	7Networks_RH_DorsAttn_Post_5.	4.65
7Networks_LH_Cont_pCun_1	7Networks_RH_SomMot_5.	4.64
7Networks_LH_SomMot_6	7Networks_RH_Limbic_TempPole_1.	4.58
7Networks_LH_DorsAttn_Post_6	7Networks_RH_Limbic_TempPole_1.	4.47
7Networks_LH_Default_PFC_4	7Networks_RH_SomMot_3.	4.4
7Networks_LH_SalVentAttn_FrOperIns_	7Networks_RH_DorsAttn_Post_4.	4.07
7Networks_LH_DorsAttn_Post_5	7Networks_RH_SomMot_6.	4.04
7Networks_LH_Default_Temp_2	7Networks_RH_DorsAttn_Post_5.	4.03
7Networks_LH_SomMot_5	7Networks_RH_DorsAttn_Post_1.	4.01
7Networks_LH_Default_PFC_6	7Networks_RH_DorsAttn_Post_5.	4.01
7Networks_LH_Vis_7	7Networks_RH_Limbic_TempPole_1.	3.99
7Networks_LH_Limbic_TempPole_1	7Networks_RH_Vis_3.	3.96
7Networks_LH_Limbic_TempPole_2	7Networks_RH_SalVentAttn_Med_2.	3.96
7Networks_LH_Default_Temp_1	7Networks_RH_Vis_6.	3.92
7Networks_LH_Vis_2	7Networks_RH_SomMot_8.	3.89
7Networks_LH_Vis_3	7Networks_RH_Limbic_TempPole_1.	3.89
7Networks_LH_Default_PFC_6	7Networks_RH_SomMot_8.	3.8
7Networks_LH_Default_PFC_6	7Networks_RH_SomMot_7.	3.79
7Networks_LH_Default_Temp_2	7Networks_RH_Vis_2.	3.75
7Networks_LH_Limbic_TempPole_1	7Networks_RH_DorsAttn_Post_1.	3.74
7Networks_LH_Limbic_TempPole_1	7Networks_RH_SomMot_8.	3.73
7Networks_LH_DorsAttn_Post_3	7Networks_RH_DorsAttn_Post_2.	3.72
7Networks_LH_DorsAttn_Post_6	7Networks_RH_Limbic_OFC_1.	3.71
7Networks_LH_Cont_Par_1	7Networks_RH_SomMot_8.	3.68
7Networks_LH_Default_Par_1	7Networks_RH_Vis_5.	3.67
7Networks_LH_Default_PFC_6	7Networks_RH_SomMot_2.	3.66

7Networks_LH_SomMot_6	7Networks_RH_Vis_2.	3.65
7Networks_LH_DorsAttn_Post_6	7Networks_RH_DorsAttn_Post_2.	3.64
7Networks_LH_Default_Par_1	7Networks_RH_DorsAttn_Post_5.	3.63
7Networks_LH_Vis_7	7Networks_RH_SomMot_6.	3.6
7Networks_LH_Vis_8	7Networks_RH_SomMot_6.	3.59
7Networks_LH_Vis_3	7Networks_RH_DorsAttn_Post_2.	3.59
7Networks_LH_DorsAttn_Post_5	7Networks_RH_SomMot_1.	3.57
7Networks_LH_Vis_5	7Networks_RH_DorsAttn_FEF_1.	3.57
7Networks_LH_DorsAttn_Post_3	7Networks_RH_SomMot_6.	3.55
7Networks_LH_DorsAttn_Post_3	7Networks_RH_SomMot_8.	3.55
7Networks_LH_Default_PFC_6	7Networks_RH_Vis_7.	3.54
7Networks_LH_Default_PFC_6	7Networks_RH_SomMot_6.	3.54
7Networks_LH_SomMot_6	7Networks_RH_DorsAttn_Post_1.	3.54
7Networks_LH_Limbic_TempPole_1	7Networks_RH_Vis_6.	3.52
7Networks_LH_DorsAttn_Post_6	7Networks_RH_Vis_3.	3.49
7Networks_LH_Default_Temp_1	7Networks_RH_Vis_3.	3.48
7Networks_LH_DorsAttn_Post_5	7Networks_RH_Limbic_OFC_1.	3.47
7Networks_LH_Default_PFC_6	7Networks_RH_DorsAttn_Post_2.	3.45
7Networks_LH_Vis_8	7Networks_RH_SomMot_8.	3.44
7Networks_LH_Vis_3	7Networks_RH_DorsAttn_FEF_1.	3.44
7Networks_LH_DorsAttn_Post_3	7Networks_RH_SomMot_7.	3.37
7Networks_LH_Default_PFC_6	7Networks_RH_SalVentAttn_Med_2.	3.37
7Networks_LH_DorsAttn_Post_2	7Networks_RH_Limbic_TempPole_1.	3.37
7Networks_LH_Vis_3	7Networks_RH_SomMot_8.	3.36
7Networks_LH_Default_Temp_1	7Networks_RH_Vis_2.	3.35
7Networks_LH_Limbic_TempPole_1	7Networks_RH_DorsAttn_FEF_1.	3.35
7Networks_LH_Default_Temp_1	7Networks_RH_Vis_8.	3.34
7Networks_LH_DorsAttn_Post_3	7Networks_RH_SalVentAttn_Med_2.	3.28
7Networks_LH_Default_PFC_6	7Networks_RH_Vis_2.	3.27
7Networks_LH_Limbic_TempPole_1	7Networks_RH_DorsAttn_Post_2.	3.27
7Networks_LH_Default_PFC_6	7Networks_RH_SomMot_3.	3.26
7Networks_LH_Default_Par_1	7Networks_RH_Vis_7.	3.24
7Networks_LH_Limbic_TempPole_1	7Networks_RH_Vis_7.	3.23
7Networks_LH_Default_pCunPCC_2	7Networks_RH_Vis_7.	3.22
7Networks_LH_SomMot_1	7Networks_RH_Default_PFCv_2.	3.2
7Networks_LH_DorsAttn_Post_3	7Networks_RH_Limbic_TempPole_1.	3.18
7Networks_LH_SomMot_1	7Networks_RH_Cont_PFCl_1.	3.18
7Networks_LH_DorsAttn_FEF_1	7Networks_RH_Vis_2.	3.17
7Networks_LH_Default_Par_1	7Networks_RH_Vis_3.	3.17
7Networks_LH_Limbic_TempPole_1	7Networks_RH_Vis_2.	3.16
7Networks_LH_Vis_8	7Networks_RH_DorsAttn_Post_2.	3.16
7Networks_LH_DorsAttn_Post_4	7Networks_RH_Vis_2.	3.13
7Networks_LH_Default_PFC_7	7Networks_RH_Vis_2.	3.13
7Networks_LH_Default_PFC_6	7Networks_RH_Vis_3.	3.12
7Networks_LH_SomMot_3	7Networks_RH_Vis_7.	3.12
7Networks_LH_Default_PFC_1	7Networks_RH_SomMot_4.	3.11
7Networks_LH_Default_Temp_1	7Networks_RH_DorsAttn_FEF_1.	3.1
7Networks_RH_SomMot_6	7Networks_RH_DorsAttn_Post_1.	6.78

7Networks_LH_DorsAttn_Post_6	7Networks_LH_Default_Temp_1.	4.86
7Networks_RH_Vis_2	7Networks_RH_SomMot_8.	4.84
7Networks_LH_Vis_7	7Networks_LH_Limbic_TempPole_1.	4.64
7Networks_RH_DorsAttn_Post_5	7Networks_RH_Limbic_TempPole_1.	4.53
7Networks_RH_SomMot_8	7Networks_RH_DorsAttn_Post_1.	4.51
7Networks_LH_DorsAttn_Post_6	7Networks_LH_Limbic_TempPole_1.	4.5
7Networks_LH_DorsAttn_Post_3	7Networks_LH_Limbic_TempPole_1.	4.49
7Networks_LH_SomMot_6	7Networks_LH_Default_PFC_6.	4.41
7Networks_LH_Vis_3	7Networks_LH_Limbic_TempPole_1.	4.39
7Networks_RH_SalVentAttn_Med_1	7Networks_RH_Limbic_TempPole_1.	4.26
7Networks_RH_DorsAttn_Post_2	7Networks_RH_DorsAttn_Post_5.	4.25
7Networks_LH_DorsAttn_FEF_1	7Networks_LH_Limbic_TempPole_1.	4.19
7Networks_RH_DorsAttn_Post_1	7Networks_RH_DorsAttn_Post_2.	4.16
7Networks_LH_DorsAttn_Post_6	7Networks_LH_Default_Temp_2.	4.15
7Networks_LH_Vis_9	7Networks_LH_Default_Temp_1.	4.11
7Networks_LH_Vis_5	7Networks_RH_SalVentAttn_TempOccP	4.09
7Networks_LH_SomMot_6	7Networks_LH_Limbic_TempPole_1.	4.05
7Networks_LH_SomMot_4	7Networks_LH_DorsAttn_Post_5.	3.98
7Networks_LH_DorsAttn_Post_6	7Networks_LH_Default_PFC_2.	3.96
7Networks_RH_SomMot_5	7Networks_RH_DorsAttn_Post_1.	3.96
7Networks_RH_Vis_5	7Networks_RH_SalVentAttn_TempOccP	3.92
7Networks_LH_DorsAttn_Post_3	7Networks_LH_SalVentAttn_FrOperIns_	3.91
7Networks_LH_SomMot_5	7Networks_LH_Cont_pCun_1.	3.8
7Networks_RH_Vis_6	7Networks_RH_Limbic_TempPole_1.	3.74
7Networks_RH_DorsAttn_Post_5	7Networks_RH_Default_PFCv_2.	3.67
7Networks_LH_SomMot_2	7Networks_LH_DorsAttn_Post_5.	3.66
7Networks_LH_Vis_3	7Networks_LH_Default_Temp_1.	3.65
7Networks_RH_SomMot_5	7Networks_RH_Cont_pCun_1.	3.63
7Networks_LH_SomMot_5	7Networks_LH_DorsAttn_Post_5.	3.62
7Networks_LH_Vis_2	7Networks_LH_DorsAttn_Post_4.	3.59
7Networks_LH_Vis_7	7Networks_LH_Default_Temp_1.	3.56
7Networks_RH_SomMot_7	7Networks_RH_DorsAttn_Post_1.	3.56
7Networks_LH_DorsAttn_FEF_1	7Networks_LH_Default_PFC_2.	3.54
7Networks_LH_SomMot_4	7Networks_LH_DorsAttn_Post_3.	3.52
7Networks_RH_Vis_3	7Networks_RH_DorsAttn_Post_1.	3.52
7Networks_LH_Vis_8	7Networks_LH_Default_PFC_7.	3.48
7Networks_RH_Vis_7	7Networks_RH_Default_Temp_3.	3.47
7Networks_RH_SomMot_8	7Networks_RH_Limbic_TempPole_1.	3.44
7Networks_RH_Vis_2	7Networks_RH_DorsAttn_FEF_1.	3.41
7Networks_RH_DorsAttn_Post_1	7Networks_RH_Limbic_TempPole_1.	3.41
7Networks_LH_DorsAttn_Post_1	7Networks_LH_Limbic_TempPole_1.	3.4
7Networks_LH_SomMot_3	7Networks_LH_Default_PFC_6.	3.39
7Networks_RH_SomMot_7	7Networks_RH_Limbic_TempPole_1.	3.39
7Networks_LH_Vis_2	7Networks_LH_SomMot_5.	3.38
7Networks_RH_SomMot_7	7Networks_RH_DorsAttn_Post_5.	3.38
7Networks_RH_Vis_8	7Networks_RH_Limbic_TempPole_1.	3.38
7Networks_RH_DorsAttn_Post_5	7Networks_RH_Default_Temp_2.	3.37
7Networks_RH_Vis_2	7Networks_RH_SomMot_5.	3.36

7Networks_RH_DorsAttn_FEF_1	7Networks_RH_Limbic_TempPole_1.	3.35
7Networks_RH_SalVentAttn_Med_2	7Networks_RH_Limbic_TempPole_1.	3.35
7Networks_LH_Vis_2	7Networks_LH_SomMot_4.	3.34
7Networks_LH_Vis_3	7Networks_LH_SomMot_6.	3.33
7Networks_LH_Vis_8	7Networks_LH_Default_PFC_6.	3.33
7Networks_RH_Vis_2	7Networks_RH_SalVentAttn_Med_2.	3.31
7Networks_RH_DorsAttn_Post_5	7Networks_RH_Default_Temp_3.	3.27
7Networks_LH_Vis_2	7Networks_LH_SomMot_6.	3.26
7Networks_LH_DorsAttn_FEF_1	7Networks_LH_Default_Temp_2.	3.25
7Networks_LH_DorsAttn_Post_6	7Networks_LH_Default_Par_1.	3.25
7Networks_LH_SomMot_1	7Networks_LH_Default_PFC_6.	3.23
7Networks_LH_SomMot_6	7Networks_LH_Cont_Par_1.	3.21
7Networks_RH_SomMot_4	7Networks_RH_DorsAttn_Post_5.	3.2
7Networks_RH_Vis_5	7Networks_RH_DorsAttn_FEF_1.	3.19
7Networks_LH_Vis_9	7Networks_LH_Limbic_TempPole_1.	3.18
7Networks_LH_Vis_8	7Networks_LH_SomMot_3.	3.17
7Networks_LH_Vis_8	7Networks_LH_SomMot_5.	3.16
7Networks_LH_Vis_3	7Networks_LH_DorsAttn_Post_2.	3.16
7Networks_LH_DorsAttn_Post_2	7Networks_LH_Limbic_TempPole_1.	3.16
7Networks_LH_SomMot_2	7Networks_LH_Cont_pCun_1.	3.16
7Networks_RH_DorsAttn_Post_2	7Networks_RH_Limbic_TempPole_1.	3.13
7Networks_LH_DorsAttn_Post_3	7Networks_LH_DorsAttn_Post_4.	3.12
7Networks_RH_Vis_8	7Networks_RH_SalVentAttn_TempOccP	3.11
7Networks_RH_SomMot_1	7Networks_RH_Limbic_TempPole_1.	3.11
7Networks_RH_Vis_7	7Networks_RH_SomMot_8.	3.1

orrelation)

Partial Correlations for 114 ROI analysis, Coef - correlation coefficient

Labels	Index	Coef	pValues
7Networks_LH_DorsAttn_Post_6	21	0.38083787	0.00027372
7Networks_RH_DorsAttn_Post_5	71	0.32173016	0.00237582
7Networks_RH_DorsAttn_Post_2	68	0.27845217	0.0090138
7Networks_RH_Vis_3	53	0.27390285	0.01025417
7Networks_RH_DorsAttn_Post_4	70	0.26235958	0.01409239
7Networks_LH_DorsAttn_Post_4	19	0.26081096	0.01469196
7Networks_LH_Vis_7	7	0.25529111	0.0170122
7Networks_LH_DorsAttn_Post_2	17	0.24973118	0.01966191
7Networks_RH_Limbic_TempPole_1	80	0.23187209	0.03069366
7Networks_LH_SomMot_5	14	0.22547384	0.03574801
7Networks_RH_SalVentAttn_TempOccPar_2	75	0.22402007	0.03698883
7Networks_RH_Default_Temp_2	92	0.22190424	0.03885893
7Networks_LH_DorsAttn_FEF_1	23	0.22160066	0.03913362
7Networks_RH_SomMot_6	64	0.21782261	0.04268969
7Networks_RH_DorsAttn_Post_1	67	0.21539392	0.04511438
7Networks_RH_Vis_7	57	0.21313017	0.04747603
7Networks_LH_SomMot_3	12	0.20962598	0.0513324
7Networks_RH_SalVentAttn_FrOperIns_1	76	0.20954421	0.05142538
7Networks_LH_DorsAttn_Post_3	18	0.20436662	0.05760051
7Networks_RH_Default_PFCdPFCm_1	96	-0.1953019	0.06986164
7Networks_LH_Default_Par_1	40	0.19502282	0.07027019
7Networks_RH_SalVentAttn_TempOccPar_1	74	0.19486129	0.07050751
7Networks_RH_DorsAttn_FEF_1	73	0.19372894	0.0721894
L_Default	107	-0.1929544	0.07335833
7Networks_RH_DorsAttn_Post_3	69	0.19131523	0.07588219
7Networks_RH_Default_PFCdPFCm_2	97	-0.1881329	0.08098069
7Networks_LH_SalVentAttn_FrOperIns_1	25	0.18651491	0.08367558
7Networks_RH_SomMot_1	59	0.18374757	0.0884498
7Networks_LH_DorsAttn_Post_1	16	0.1837223	0.08849437
7Networks_LH_SomMot_4	13	0.18360751	0.08869706
7Networks_LH_DorsAttn_Post_5	20	0.18327679	0.08928304
7Networks_LH_SalVentAttn_Med_2	29	0.18249537	0.09067979
7Networks_RH_Cont_Cing_1	87	-0.1813414	0.09277397
7Networks_LH_Default_Temp_1	38	0.17610709	0.10275563
L_Frontoparietal	106	-0.1753468	0.10427272
7Networks_LH_SomMot_6	15	0.1725407	0.11002393
7Networks_RH_SomMot_4	62	0.17160323	0.1119992
L_Ventral_attention	104	-0.1694876	0.11655783
7Networks_RH_Vis_2	52	0.16879419	0.1180826
7Networks_RH_SomMot_3	61	0.16798937	0.11987165
R_Default	114	-0.1676051	0.12073313
7Networks_LH_Default_Temp_2	39	0.16535017	0.12588491
R_Frontoparietal	113	-0.1629622	0.1315223
7Networks_LH_Vis_8	8	0.16052682	0.13746736

R_Visual	108	-0.1584482	0.14270078
7Networks_RH_Default_PFCv_2	95	0.15828864	0.14310855
7Networks_LH_SomMot_2	11	0.15582528	0.14951708
7Networks_RH_SomMot_8	66	0.15439519	0.1533349
7Networks_RH_Cont_Par_2	82	-0.154265	0.15368602
7Networks_LH_Cont_Cing_1	37	-0.1494057	0.1672257
7Networks_RH_Default_Temp_3	93	0.14798311	0.17135132
7Networks_RH_SomMot_2	60	0.14754294	0.17264286
7Networks_LH_SalVentAttn_ParOper_1	24	0.14709455	0.1739659
7Networks_LH_Vis_4	4	-0.143428	0.18506461
7Networks_RH_SalVentAttn_Med_1	77	0.14186375	0.18995293
7Networks_RH_SalVentAttn_Med_2	78	0.13988972	0.19625436
7Networks_LH_Default_PFC_3	44	-0.1396278	0.19710151
7Networks_RH_SomMot_7	65	0.1393316	0.19806292
7Networks_LH_Vis_3	3	0.13836901	0.20121029
7Networks_LH_SomMot_1	10	0.13779926	0.20308995
7Networks_LH_Limbic_OFC_1	31	-0.1355391	0.21067035
7Networks_RH_Cont_PFCl_1	83	-0.1334081	0.21799961
7Networks_LH_DorsAttn_PrCv_1	22	0.13167746	0.22408284
7Networks_LH_SalVentAttn_FrOperIns_2	26	0.13127673	0.22550828
7Networks_RH_DorsAttn_PrCv_1	72	0.12990864	0.23042257
7Networks_LH_Cont_pCun_1	36	0.12508474	0.24834515
7Networks_LH_Default_PFC_6	47	0.12489234	0.24907932
7Networks_RH_Vis_8	58	0.12394771	0.25270548
7Networks_LH_Limbic_TempPole_2	33	-0.1211996	0.26345957
7Networks_LH_Limbic_TempPole_1	32	0.12075945	0.26521021
7Networks_RH_Default_Par_1	90	-0.1204014	0.2666401
7Networks_LH_SalVentAttn_Med_3	30	0.11908279	0.2719515
7Networks_RH_SomMot_5	63	0.11747541	0.27852151
7Networks_RH_Vis_6	56	0.1169425	0.28072299
7Networks_RH_Default_PFCv_1	94	-0.1123009	0.30038752
7Networks_RH_Cont_Par_1	81	0.10537665	0.33135927
7Networks_LH_Vis_5	5	-0.1039128	0.33815775
7Networks_LH_Vis_2	2	0.1025131	0.34474014
7Networks_RH_Cont_PFCmp_1	88	-0.0944106	0.38440328
L_Visual	101	-0.0898585	0.40784149
R_Limbic	112	-0.0834116	0.44242878
R_Ventral_attention	111	-0.0798159	0.46241327
7Networks_RH_Default_pCunPCC_1	99	0.07418684	0.49466861
7Networks_LH_Cont_PFCl_1	35	-0.0740821	0.49527972
7Networks_LH_Vis_9	9	0.07113235	0.51265315
7Networks_LH_Default_Par_2	41	-0.0689942	0.5254386
L_Dorsa_lattention	103	-0.0684524	0.52870368
7Networks_LH_SalVentAttn_Med_1	28	-0.0619627	0.56858394
7Networks_LH_Default_PFC_5	46	-0.0583079	0.59164759
7Networks_LH_Default_PFC_4	45	-0.0555354	0.60941989

R_Dorsa_lattention	110	-0.0526807	0.62795829
7Networks_LH_Cont_Par_1	34	0.05006251	0.64516667
7Networks_RH_Vis_1	51	0.04739001	0.66292645
L_Limbic	105	0.04737625	0.66301845
7Networks_LH_Default_pCunPCC_2	50	-0.0445171	0.68222746
7Networks_LH_Default_PFC_7	48	-0.0433242	0.69030303
7Networks_LH_Default_pCunPCC_1	49	-0.0322436	0.76686735
7Networks_RH_Cont_PFCl_2	84	-0.032101	0.76786858
7Networks_RH_Cont_PFCl_4	86	0.0280021	0.79682448
7Networks_LH_Default_PFC_2	43	0.02727153	0.80201561
7Networks_RH_Default_Temp_1	91	-0.0256984	0.81322222
7Networks_RH_Default_PFCdPFCm_3	98	-0.0240574	0.82495305
R_Somatomotor	109	-0.0226716	0.83488956
7Networks_LH_Vis_1	1	-0.022484	0.83623666
7Networks_RH_Vis_5	55	0.02235612	0.83715521
7Networks_RH_Cont_pCun_1	89	0.01813473	0.86759302
7Networks_RH_Limbic_OFC_1	79	-0.0179321	0.86905939
7Networks_LH_SalVentAttn_PFCl_1	27	0.00978835	0.92830214
7Networks_RH_Vis_4	54	0.00833688	0.93891164
7Networks_LH_Default_PFC_1	42	-0.0080221	0.94121391
L_Somatomotor	102	0.00758511	0.94441106
7Networks_LH_Vis_6	6	0.0065034	0.95232854
7Networks_RH_Default_pCunPCC_2	100	0.00433367	0.96822282
7Networks_RH_Cont_PFCl_3	85	0.00383359	0.97188814

Model estimates (Beta) for NfL*Group 114 ROI analysis

Labels	Index	Beta	pValues
7Networks_RH_Default_pCunPCC_2	100	-0.0084019	0.0126317
7Networks_RH_Limbic_TempPole_1	80	0.00824561	0.02727556
7Networks_RH_Vis_7	57	0.01003444	0.03053308
7Networks_LH_Default_pCunPCC_2	50	-0.006533	0.05304198
7Networks_LH_Limbic_TempPole_1	32	0.00696828	0.07688301
7Networks_RH_Vis_2	52	0.00908141	0.07768805
L_Visual	101	-0.0044572	0.0894942
7Networks_LH_Default_PFC_2	43	-0.0046697	0.09855991
7Networks_LH_Vis_2	2	0.00798622	0.09909815
7Networks_LH_Default_pCunPCC_1	49	-0.0062478	0.10819069
7Networks_LH_Vis_5	5	0.00710856	0.11868966
R_Default	114	0.00578	0.12234872
7Networks_RH_Vis_4	54	0.0061086	0.13030309
7Networks_RH_Default_pCunPCC_1	99	-0.0060501	0.13434153
7Networks_RH_Cont_pCun_1	89	-0.0056538	0.14240889
7Networks_RH_Limbic_OFC_1	79	0.00517729	0.15203422
7Networks_LH_Default_PFC_1	42	-0.0044583	0.15583609
7Networks_RH_Vis_3	53	0.00645256	0.17890335
7Networks_LH_Default_PFC_3	44	-0.0055543	0.19493815
7Networks_LH_Vis_8	8	0.00633326	0.20115637
7Networks_LH_SomMot_5	14	0.00586591	0.21301912
7Networks_LH_SalVentAttn_FrOperIns_2	26	-0.0067394	0.22028585
7Networks_LH_SalVentAttn_PFCl_1	27	-0.0047109	0.2306918
7Networks_RH_SomMot_7	65	0.00555434	0.24625336
7Networks_LH_Vis_4	4	0.00391088	0.25279152
7Networks_RH_SomMot_6	64	0.00571047	0.26013372
7Networks_RH_Default_PFCv_2	95	-0.0041773	0.26189878
7Networks_RH_SalVentAttn_FrOperIns_1	76	-0.0049585	0.28415999
7Networks_LH_SomMot_4	13	0.00368353	0.2895266
7Networks_RH_Cont_PFCl_2	84	-0.0041967	0.29732882
7Networks_LH_DorsAttn_Post_6	21	0.00512781	0.30441421
7Networks_RH_Vis_8	58	0.00477561	0.32091199
7Networks_LH_Default_Temp_2	39	-0.0039472	0.3277619
7Networks_RH_DorsAttn_Post_2	68	0.00516452	0.33320793
7Networks_RH_SalVentAttn_TempOccPar_2	75	-0.0042323	0.34217649
7Networks_RH_Default_PFCdPFCm_1	96	-0.00409	0.35192264
7Networks_LH_DorsAttn_Post_1	16	0.00448691	0.3546859
7Networks_LH_SalVentAttn_FrOperIns_1	25	0.00400313	0.35481327
7Networks_RH_SomMot_8	66	0.00426476	0.37612222
L_Default	107	0.0030007	0.3866495
7Networks_RH_Vis_5	55	0.00418795	0.38857231
7Networks_RH_DorsAttn_Post_4	70	0.00415197	0.39168076
7Networks_LH_SomMot_6	15	0.00370093	0.39848063
7Networks_LH_Default_PFC_4	45	0.00279917	0.41125798

7Networks_RH_DorsAttn_PrCv_1	72	-0.0038059	0.41622094
7Networks_LH_Vis_3	3	0.00466098	0.41689605
7Networks_RH_SomMot_5	63	0.00366702	0.41821529
7Networks_RH_SomMot_3	61	-0.0032682	0.4213577
7Networks_RH_SomMot_4	62	0.00347737	0.43112261
7Networks_LH_Cont_PFCl_1	35	-0.0030827	0.44069335
L_Ventral_attention	104	0.00327527	0.44260269
7Networks_RH_Cont_Par_2	82	-0.0027136	0.44558283
7Networks_LH_DorsAttn_PrCv_1	22	-0.0033894	0.45509159
L_Dorsa_lattention	103	0.00214609	0.45520844
7Networks_RH_Default_PFCv_1	94	-0.0026707	0.4640168
R_Frontoparietal	113	0.00292496	0.47097595
7Networks_LH_Default_Par_2	41	-0.0027448	0.47233893
7Networks_LH_SalVentAttn_ParOper_1	24	-0.0027081	0.48487932
7Networks_RH_Cont_Cing_1	87	-0.0025741	0.48765165
7Networks_LH_DorsAttn_Post_3	18	0.00338303	0.50137885
7Networks_RH_Cont_PFCl_4	86	-0.0022684	0.50665775
7Networks_RH_Default_PFCdPFCm_3	98	-0.0023488	0.52195306
7Networks_LH_SomMot_3	12	-0.0025826	0.53209957
7Networks_LH_Default_Par_1	40	-0.0021201	0.57287057
L_Frontoparietal	106	0.00225108	0.59112683
7Networks_LH_SalVentAttn_Med_3	30	-0.0022445	0.59914401
7Networks_LH_Default_PFC_6	47	0.00215455	0.60007185
7Networks_LH_Cont_Cing_1	37	-0.001958	0.61600786
7Networks_RH_Default_Temp_2	92	-0.0018977	0.61837909
7Networks_RH_Default_Temp_3	93	-0.0018401	0.62530179
7Networks_LH_DorsAttn_Post_4	19	0.0024958	0.62752874
7Networks_RH_Cont_Par_1	81	-0.0017869	0.63816172
R_Visual	108	0.00150895	0.64114803
7Networks_LH_DorsAttn_Post_5	20	-0.0014508	0.65029367
7Networks_LH_Vis_9	9	0.00228049	0.65799406
R_Ventral_attention	111	0.00170947	0.67798966
7Networks_LH_SomMot_1	10	-0.0021636	0.68062911
7Networks_RH_SomMot_1	59	-0.0017344	0.68730166
7Networks_RH_Default_PFCdPFCm_2	97	-0.0015266	0.69358806
7Networks_LH_Limbic_OFC_1	31	0.00149611	0.69494868
7Networks_LH_Vis_6	6	0.00168803	0.69616973
7Networks_RH_Cont_PFCmp_1	88	-0.0015099	0.7264135
7Networks_LH_Cont_Par_1	34	0.00128089	0.74286103
7Networks_RH_Default_Par_1	90	-0.0009559	0.76777887
7Networks_RH_Default_Temp_1	91	-0.001	0.768
7Networks_LH_DorsAttn_Post_2	17	0.00145546	0.76823926
7Networks_LH_Limbic_TempPole_2	33	0.00116442	0.77659874
7Networks_RH_SalVentAttn_Med_1	77	-0.0012453	0.78684107
7Networks_RH_Cont_PFCl_3	85	-0.0010218	0.79910899
7Networks_LH_DorsAttn_FEF_1	23	0.0010996	0.83406397

7Networks_LH_Vis_1	1	-0.0007569	0.83913083
7Networks_RH_DorsAttn_FEF_1	73	0.00107934	0.84060222
L_Somatomotor	102	0.00074137	0.84182714
7Networks_RH_SalVentAttn_Med_2	78	-0.0009285	0.84554893
L_Limbic	105	0.00058876	0.84949951
7Networks_RH_Cont_PFCl_1	83	0.00071321	0.85823803
7Networks_RH_Vis_6	56	0.00070922	0.85982318
R_Dorsa_lattention	110	0.00047106	0.86024627
7Networks_RH_Vis_1	51	0.00064289	0.8682519
7Networks_RH_DorsAttn_Post_5	71	-0.0007078	0.88648654
7Networks_LH_Default_PFC_7	48	-0.0005293	0.90035001
7Networks_LH_SalVentAttn_Med_1	28	-0.0005177	0.9037724
7Networks_LH_Default_Temp_1	38	-0.0004255	0.90432927
7Networks_LH_SomMot_2	11	-0.0004705	0.91046174
7Networks_RH_SalVentAttn_TempOccPar_[illegible]	74	-0.0004018	0.91644164
7Networks_RH_SomMot_2	60	-0.0003752	0.92899906
7Networks_LH_Vis_7	7	0.00038814	0.93129901
R_Somatomotor	109	0.00031171	0.9322901
R_Limbic	112	0.00022652	0.94800026
7Networks_LH_Default_PFC_5	46	-0.0002524	0.95312586
7Networks_RH_DorsAttn_Post_1	67	0.00020134	0.95867267
7Networks_RH_DorsAttn_Post_3	69	-0.0001996	0.96544407
7Networks_LH_SalVentAttn_Med_2	29	-0.0001823	0.97032908
7Networks_LH_Cont_pCun_1	36	-0.0001026	0.97998493

Partial Correlations for 514 ROI analysis, Coef - correlation coefficient

labels	Index	Coef	pValues
7Networks_LH_DorsAttn_Post_16	102	0.49871991	0.00041927
7Networks_RH_Limbic_TempPole_2	406	0.49567243	0.00046052
7Networks_LH_DorsAttn_Post_19	105	0.4865378	0.00060699
7Networks_LH_DorsAttn_Post_18	104	0.47946886	0.0007477
7Networks_RH_Vis_23	273	0.43275025	0.00266671
7Networks_RH_DorsAttn_Post_21	355	0.4272898	0.00305926
7Networks_LH_SomMot_20	59	0.42641724	0.00312648
7Networks_RH_DorsAttn_Post_23	357	0.4254608	0.00320167
7Networks_LH_SalVentAttn_TempOcc_1	117	0.4119213	0.00444979
7Networks_RH_DorsAttn_Post_20	354	0.41085523	0.00456407
7Networks_RH_DorsAttn_Post_14	348	0.40344969	0.00543118
7Networks_RH_DorsAttn_Post_11	345	0.39874202	0.00605415
7Networks_LH_Vis_3	3	0.39589627	0.00646011
7Networks_RH_Vis_4	254	0.3942209	0.00671
7Networks_LH_Vis_19	19	0.39414761	0.00672112
7Networks_RH_Default_PFCv_2	472	-0.3901179	0.00735769
7Networks_LH_Limbic_TempPole_7	151	0.38940024	0.00747636
7Networks_RH_DorsAttn_Post_24	358	0.38918831	0.00751173
7Networks_RH_Vis_32	282	0.38748334	0.00780152
7Networks_LH_Limbic_TempPole_5	149	0.38560016	0.0081328
7Networks_RH_Default_pCunPCC_6	495	-0.383802	0.00846038
7Networks_LH_Vis_35	35	0.382795	0.00864877
7Networks_LH_DorsAttn_Post_17	103	0.38058909	0.00907412
7Networks_LH_Vis_29	29	0.37920121	0.00935088
7Networks_LH_SomMot_44	83	0.37898201	0.00939525
7Networks_LH_SomMot_37	76	0.37114176	0.01110595
7Networks_RH_DorsAttn_Post_10	344	0.36820557	0.01181203
7Networks_RH_Vis_27	277	0.36815237	0.01182518
7Networks_RH_DorsAttn_Post_18	352	0.36777842	0.01191789
7Networks_RH_Limbic_TempPole_5	409	0.36774429	0.01192638
7Networks_RH_DorsAttn_Post_2	336	0.36751763	0.01198291
7Networks_LH_Vis_36	36	0.36743039	0.01200474
7Networks_LH_SomMot_33	72	0.3665769	0.01222001
7Networks_LH_DorsAttn_Post_5	91	0.36343567	0.01304098
7Networks_RH_SalVentAttn_Med_9	393	0.36248051	0.01329977
7Networks_LH_Default_pCunPCC_9	245	-0.362033	0.0134225
7Networks_LH_Limbic_TempPole_8	152	0.36081786	0.01376069
7Networks_RH_SomMot_47	332	0.36064361	0.01380978
7Networks_RH_Vis_13	263	0.36041396	0.01387469
7Networks_LH_SomMot_23	62	0.36021808	0.01393027
7Networks_RH_Limbic_TempPole_4	408	0.35981952	0.01404394
7Networks_RH_SomMot_46	331	0.35956317	0.01411747
7Networks_LH_Cont_PFCd_1	164	0.35729422	0.01478269
7Networks_LH_Default_Temp_1	186	0.35662368	0.01498431

7Networks_LH_SomMot_30	69	0.35346557	0.01596564
7Networks_LH_Default_Par_1	198	0.35104624	0.01675383
7Networks_LH_SalVentAttn_FrOperIns_1	118	0.3487661	0.0175266
7Networks_RH_SomMot_25	310	0.34426262	0.01914176
7Networks_LH_DorsAttn_Post_3	89	0.34393633	0.0192635
7Networks_LH_SalVentAttn_Med_9	137	0.34317578	0.01954982
7Networks_RH_Cont_PFCmp_2	451	-0.3418594	0.02005387
7Networks_LH_Default_Par_2	199	0.34142136	0.02022401
7Networks_LH_SomMot_40	79	0.3407726	0.02047822
7Networks_RH_Limbic_TempPole_1	405	0.34067187	0.02051793
7Networks_RH_SalVentAttn_Med_10	394	0.33975964	0.02088052
7Networks_LH_Limbic_TempPole_3	147	0.33716834	0.02194003
7Networks_RH_DorsAttn_Post_7	341	0.33675877	0.02211156
7Networks_LH_DorsAttn_Post_4	90	0.33568298	0.02256745
7Networks_LH_SomMot_43	82	0.33309086	0.02369843
7Networks_RH_Cont_PFCl_18	441	0.33306443	0.0237102
7Networks_RH_Default_Temp_3	463	0.33213704	0.02412637
7Networks_LH_SomMot_28	67	0.3312871	0.02451313
7Networks_RH_SomMot_20	305	0.33090325	0.02468949
7Networks_RH_Default_PFCdPFCm_5	479	-0.3292945	0.02544021
7Networks_RH_DorsAttn_Post_1	335	0.32570457	0.02718441
7Networks_RH_Default_Temp_8	468	0.32522101	0.02742678
7Networks_LH_SalVentAttn_ParOper_4	115	0.32342693	0.02834182
7Networks_LH_DorsAttn_Post_12	98	0.32098844	0.0296261
7Networks_RH_SomMot_41	326	0.31988677	0.03022197
7Networks_RH_Vis_6	256	0.31703897	0.03180846
7Networks_LH_Vis_8	8	0.31645411	0.03214267
7Networks_RH_SomMot_45	330	0.31426352	0.03342036
7Networks_LH_DorsAttn_Post_9	95	0.31385796	0.03366145
7Networks_RH_Limbic_TempPole_10	414	0.31233485	0.03457974
7Networks_RH_SomMot_37	322	0.31173711	0.03494571
7Networks_LH_Default_Par_9	206	-0.3098253	0.03613777
7Networks_RH_DorsAttn_Post_5	339	0.30858131	0.0369312
7Networks_RH_Default_Temp_10	470	0.30799902	0.0373075
7Networks_RH_DorsAttn_Post_17	351	0.30799744	0.03730852
7Networks_LH_DorsAttn_FEF_4	109	0.30777922	0.03745036
7Networks_RH_Default_pCunPCC_7	496	-0.3067526	0.03812357
7Networks_RH_Vis_2	252	0.30672892	0.03813921
7Networks_LH_Default_PFC_26	232	0.30368975	0.0401913
7Networks_LH_Limbic_OFC_6	144	0.30312014	0.04058579
7Networks_LH_DorsAttn_Post_10	96	0.30305279	0.04063264
7Networks_LH_SomMot_47	86	0.30177361	0.04153094
7Networks_LH_Default_Temp_12	197	0.30082491	0.04220759
7Networks_RH_SomMot_44	329	0.30081147	0.04221724
7Networks_RH_DorsAttn_Post_6	340	0.30001897	0.04278947
7Networks_LH_SalVentAttn_FrOperIns_9	126	0.29963971	0.04306555

7Networks_LH_SalVentAttn_Med_2	130	0.29668884	0.0452636
7Networks_LH_Default_Temp_8	193	0.29654176	0.0453755
7Networks_RH_Cont_PFCl_6	429	0.29513936	0.04645383
7Networks_RH_SomMot_21	306	0.29498631	0.04657276
7Networks_RH_Default_Par_1	453	0.29335459	0.04785618
7Networks_LH_SalVentAttn_Med_8	136	0.29320793	0.04797292
7Networks_RH_DorsAttn_PrCv_3	365	0.2920953	0.04886616
7Networks_LH_SomMot_19	58	0.29030404	0.05033248
7Networks_LH_Cont_Par_1	155	0.28971725	0.05082049
7Networks_RH_Vis_5	255	0.28923288	0.0512262
7Networks_RH_Limbic_OFC_4	401	0.28686952	0.05324345
7Networks_LH_SomMot_39	78	0.28627925	0.05375715
7Networks_LH_DorsAttn_Post_14	100	0.2861798	0.05384409
7Networks_RH_DorsAttn_Post_9	343	0.28523727	0.0546737
7Networks_LH_SomMot_11	50	0.28490979	0.05496434
7Networks_LH_SalVentAttn_ParOper_2	113	0.28395951	0.05581477
7Networks_LH_DorsAttn_Post_6	92	0.28381361	0.05594628
7Networks_LH_DorsAttn_Post_8	94	0.2837172	0.05603331
7Networks_RH_SomMot_43	328	0.28176447	0.05781965
7Networks_RH_DorsAttn_Post_13	347	0.28129232	0.05825836
7Networks_RH_Default_Par_8	460	-0.2810835	0.05845328
7Networks_RH_SomMot_22	307	0.28055697	0.05894693
7Networks_RH_Default_Par_5	457	-0.2794099	0.06003408
7Networks_LH_SalVentAttn_ParOper_1	112	0.27880778	0.06061109
7Networks_LH_SomMot_9	48	0.27880424	0.0606145
7Networks_RH_SomMot_17	302	0.27807299	0.06132127
7Networks_RH_SomMot_38	323	0.278047	0.06134651
7Networks_LH_Vis_7	7	0.2767776	0.06258946
7Networks_LH_Default_PFC_25	231	0.27670077	0.06266533
7Networks_RH_SalVentAttn_Med_8	392	0.27638252	0.06298039
7Networks_RH_Limbic_TempPole_6	410	0.27615591	0.06320549
7Networks_LH_Default_Temp_9	194	0.27544877	0.06391206
7Networks_RH_SomMot_14	299	0.27510741	0.0642554
7Networks_LH_SomMot_10	49	0.2749416	0.0644227
7Networks_RH_SomMot_18	303	0.2745562	0.06481289
7Networks_LH_Vis_6	6	0.27433381	0.06503891
7Networks_LH_Default_Temp_5	190	0.27379307	0.06559107
7Networks_LH_SomMot_21	60	0.27349327	0.06589882
7Networks_RH_SomMot_30	315	0.27320563	0.06619516
7Networks_LH_SomMot_38	77	0.27259156	0.06683133
7Networks_LH_Vis_39	39	0.27198099	0.06746871
7Networks_RH_SomMot_35	320	0.27168547	0.06777892
L_Visual	501	-0.2714603	0.068016
7Networks_RH_Vis_28	278	0.27096968	0.068535
7Networks_LH_DorsAttn_Post_1	87	0.27051695	0.06901667
7Networks_RH_Vis_33	283	0.27019022	0.06936594

7Networks_RH_Default_pCunPCC_10	499	0.27016898	0.0693887
7Networks_LH_Default_PFC_11	217	-0.2698752	0.06970401
7Networks_RH_Vis_30	280	0.2685263	0.07116647
7Networks_RH_Default_Par_4	456	0.26836036	0.07134804
7Networks_RH_Vis_35	285	0.26793151	0.07181899
7Networks_LH_DorsAttn_Post_15	101	0.26752779	0.07226459
7Networks_RH_Cont_PFCmp_1	450	-0.266248	0.07369156
7Networks_LH_Default_PFC_2	208	-0.2657706	0.07422959
7Networks_LH_Default_PFC_15	221	-0.2651604	0.07492163
7Networks_LH_Cont_PFCv_1	176	-0.2645995	0.07556235
7Networks_LH_DorsAttn_Post_2	88	0.26311549	0.07727828
7Networks_LH_SomMot_27	66	0.26293033	0.07749451
7Networks_RH_Limbic_TempPole_3	407	0.26272796	0.07773138
7Networks_RH_Cont_PFCl_2	425	0.26267979	0.07778785
7Networks_LH_Default_Temp_6	191	0.26061991	0.08023281
7Networks_RH_DorsAttn_FEF_3	361	0.25893376	0.0822787
7Networks_LH_Cont_pCun_1	177	0.25860529	0.08268196
7Networks_RH_Vis_9	259	0.25851675	0.08279092
7Networks_LH_SalVentAttn_FrOperIns_10	127	0.25819289	0.08319045
7Networks_LH_SalVentAttn_Med_6	134	0.25813871	0.08325743
7Networks_LH_SalVentAttn_Med_10	138	0.25787605	0.08358277
7Networks_RH_Default_PFCv_4	474	0.25697749	0.08470326
7Networks_LH_SalVentAttn_FrOperIns_7	124	0.25653178	0.08526339
7Networks_LH_SomMot_16	55	0.25466228	0.08764436
7Networks_RH_Default_PFCdPFCm_2	476	-0.2543087	0.0881004
7Networks_LH_DorsAttn_FEF_1	106	0.2536122	0.08900427
7Networks_RH_Vis_29	279	0.25316761	0.08958496
7Networks_LH_Default_Temp_4	189	0.25009084	0.09368463
7Networks_LH_SomMot_18	57	0.2498781	0.09397338
7Networks_RH_DorsAttn_Post_3	337	0.2483924	0.09600915
7Networks_LH_SomMot_17	56	0.2470291	0.09790703
7Networks_LH_Limbic_TempPole_2	146	0.24604573	0.09929386
7Networks_RH_SalVentAttn_Med_2	386	0.24591084	0.09948526
7Networks_LH_Vis_4	4	0.24531388	0.10033575
7Networks_RH_SalVentAttn_Med_6	390	0.2441502	0.10200973
7Networks_RH_DorsAttn_Post_19	353	0.24317764	0.10342517
7Networks_RH_Default_Par_7	459	-0.2428302	0.10393441
7Networks_RH_Default_Par_2	454	0.23929384	0.10922824
7Networks_LH_Default_PFC_6	212	-0.2382587	0.11081612
7Networks_LH_DorsAttn_FEF_3	108	0.23660283	0.11339261
7Networks_RH_DorsAttn_Post_22	356	0.23509193	0.11578305
7Networks_RH_SomMot_33	318	0.23428005	0.11708327
7Networks_RH_Cont_PFCl_8	431	-0.2329873	0.11917629
7Networks_RH_SomMot_16	301	0.23265046	0.11972636
7Networks_RH_Cont_PFCl_19	442	0.23238872	0.12015507
7Networks_RH_SomMot_49	334	0.23077259	0.12282784

7Networks_LH_SomMot_13	52	0.2297118	0.1246064
7Networks_LH_SomMot_22	61	0.22884637	0.12607173
7Networks_LH_SomMot_35	74	0.22750469	0.12836897
7Networks_RH_SomMot_10	295	0.22740997	0.12853233
7Networks_RH_Default_Temp_2	462	0.2264559	0.1301865
7Networks_RH_Vis_31	281	0.22567849	0.13154611
7Networks_LH_SomMot_31	70	0.22393872	0.13462727
7Networks_RH_SalVentAttn_FrOperIns_3	377	0.22243904	0.13732617
7Networks_LH_SomMot_42	81	0.22214204	0.13786542
7Networks_RH_Vis_7	257	0.22192581	0.138259
7Networks_RH_Default_Temp_1	461	0.2211931	0.1395989
7Networks_RH_SalVentAttn_TempOccPar_	372	0.22042856	0.14100727
7Networks_LH_SomMot_45	84	0.22011042	0.14159643
7Networks_RH_Cont_PFCl_5	428	-0.219909	0.14197038
7Networks_RH_SomMot_2	287	0.21945313	0.1428194
7Networks_LH_SalVentAttn_Med_7	135	0.21914755	0.14339064
7Networks_LH_Default_Temp_11	196	0.21835647	0.1448773
7Networks_RH_Default_PFCdPFCm_6	480	-0.2182305	0.14511501
7Networks_RH_SomMot_13	298	0.21664892	0.14812504
7Networks_LH_Vis_34	34	0.21640863	0.14858635
7Networks_RH_Default_PFCdPFCm_11	485	-0.2152656	0.15079524
7Networks_RH_SalVentAttn_TempOccPar_	367	0.21478984	0.15172172
7Networks_RH_SomMot_32	317	0.21457967	0.15213232
7Networks_LH_Vis_1	1	0.21416487	0.15294512
7Networks_RH_Vis_34	284	0.21370581	0.15384834
7Networks_RH_SomMot_40	325	0.21295207	0.15533986
7Networks_LH_Vis_12	12	0.21272177	0.15579769
7Networks_LH_SomMot_41	80	0.21140978	0.15842475
7Networks_RH_SalVentAttn_Med_13	397	0.21092483	0.15940395
7Networks_RH_Cont_pCun_3	446	-0.2108727	0.15950952
7Networks_RH_Cont_PFCl_14	437	-0.2094779	0.16235179
7Networks_RH_DorsAttn_Post_15	349	0.20909184	0.16314495
7Networks_RH_SomMot_9	294	0.20860639	0.16414634
7Networks_RH_SomMot_24	309	0.2078076	0.16580381
7Networks_RH_Cont_PFCl_13	436	-0.2072714	0.16692312
7Networks_LH_Cont_Par_2	156	-0.206177	0.16922495
7Networks_RH_Cont_PFCl_10	433	0.20478983	0.17217531
7Networks_RH_SomMot_8	293	0.20394039	0.17400024
7Networks_RH_SomMot_31	316	0.20381743	0.17426555
7Networks_RH_Default_PFCdPFCm_8	482	-0.2021274	0.1779418
7Networks_RH_Vis_16	266	-0.2009951	0.18043587
7Networks_LH_Default_PFC_13	219	-0.2009559	0.18052261
7Networks_LH_Limbic_OFC_3	141	0.20016318	0.18228411
7Networks_LH_Vis_13	13	-0.1998728	0.18293238
7Networks_LH_Default_Par_4	201	0.19961193	0.18351628
7Networks_LH_Cont_PFCd_2	165	0.19852128	0.18597166

7Networks_RH_SomMot_6	291	0.19848879	0.18604517
7Networks_LH_SalVentAttn_FrOperIns_4	121	0.19845665	0.18611789
7Networks_RH_SalVentAttn_TempOccPar_	366	0.19843933	0.1861571
7Networks_LH_Limbic_TempPole_1	145	0.19784773	0.18749968
7Networks_LH_Default_pCunPCC_12	248	0.19761462	0.18803061
7Networks_LH_Cont_PFCl_4	170	-0.1975388	0.18820343
7Networks_RH_Default_PFCv_1	471	0.19723258	0.18890302
7Networks_RH_Vis_12	262	-0.1957317	0.19235826
7Networks_RH_SomMot_4	289	0.19546697	0.19297238
7Networks_LH_SomMot_25	64	0.1953162	0.19332272
7Networks_RH_SalVentAttn_FrOperIns_6	380	0.19462777	0.19492819
7Networks_RH_SalVentAttn_Med_4	388	0.19438039	0.1955074
7Networks_RH_SalVentAttn_FrOperIns_7	381	0.19356849	0.19741691
7Networks_LH_Default_PFC_14	220	0.19339042	0.19783748
7Networks_RH_DorsAttn_Post_8	342	0.19302091	0.19871219
7Networks_RH_SalVentAttn_FrOperIns_1	375	0.19274754	0.19936107
7Networks_RH_DorsAttn_Post_12	346	0.19193218	0.20130532
7Networks_RH_SomMot_36	321	0.19034742	0.20512233
7Networks_RH_SomMot_27	312	0.18946331	0.20727372
7Networks_LH_Default_PFC_8	214	0.18910483	0.20815053
7Networks_RH_DorsAttn_FEF_4	362	0.18859951	0.20939089
7Networks_LH_Default_Temp_7	192	0.18598364	0.21589447
7Networks_RH_Cont_pCun_1	444	0.18478557	0.21891952
7Networks_LH_Limbic_TempPole_4	148	0.1837902	0.22145502
7Networks_RH_SalVentAttn_Med_11	395	0.1834718	0.22227035
7Networks_LH_Default_PFC_16	222	-0.1827936	0.22401401
7Networks_RH_SalVentAttn_TempOccPar_	371	0.1814051	0.227613
7Networks_LH_DorsAttn_Post_11	97	0.18055909	0.22982531
7Networks_LH_DorsAttn_Post_13	99	0.18030722	0.23048677
7Networks_LH_Vis_38	38	0.17930874	0.23312191
7Networks_LH_SomMot_34	73	0.17894507	0.23408681
R_Visual	508	-0.1789008	0.23420449
7Networks_LH_Default_PFC_7	213	0.17872848	0.23466274
7Networks_LH_SalVentAttn_Med_1	129	0.1780115	0.23657623
7Networks_RH_Vis_19	269	0.17793762	0.23677401
7Networks_RH_Vis_21	271	0.1777466	0.23728587
7Networks_LH_Default_PHC_2	250	0.1775815	0.23772889
7Networks_RH_Cont_Par_5	419	-0.1767718	0.23990986
7Networks_LH_SomMot_7	46	0.17670792	0.24008244
7Networks_RH_Limbic_TempPole_7	411	0.17645878	0.24075656
L_Ventral_attention	504	-0.1756461	0.24296444
7Networks_RH_SomMot_1	286	0.17552233	0.24330188
7Networks_RH_SomMot_42	327	0.17466432	0.24564994
7Networks_LH_Vis_31	31	0.17347174	0.24893902
7Networks_RH_Cont_Cing_1	447	-0.1716144	0.25412037
7Networks_RH_Vis_11	261	0.17019372	0.25813231

7Networks_LH_Cont_Par_3	157	-0.1699194	0.25891192
7Networks_LH_SomMot_36	75	0.16958748	0.25985707
7Networks_LH_Vis_14	14	0.16769861	0.26528033
7Networks_RH_Default_Temp_5	465	-0.1675056	0.26583857
7Networks_RH_Default_pCunPCC_2	491	0.16735769	0.26626713
7Networks_RH_Default_PFCdPFCm_3	477	-0.1668776	0.26766091
7Networks_RH_Vis_8	258	-0.1666586	0.26829832
7Networks_RH_Vis_1	251	0.16618648	0.26967577
7Networks_RH_Cont_Par_6	420	-0.1661575	0.26976057
7Networks_RH_SalVentAttn_FrOperIns_2	376	0.16544328	0.27185365
7Networks_LH_Vis_18	18	0.16498771	0.2731944
7Networks_RH_Vis_26	276	0.16415529	0.27565549
7Networks_RH_SomMot_3	288	0.16215528	0.28162811
7Networks_RH_SomMot_12	297	0.16167899	0.28306287
7Networks_RH_Limbic_OFC_1	398	0.16150467	0.28358918
L_Frontoparietal	506	-0.1601298	0.28776256
7Networks_RH_Vis_18	268	0.1595754	0.28945667
7Networks_LH_Vis_28	28	0.15903251	0.29112193
7Networks_RH_SomMot_15	300	0.15792705	0.29453191
7Networks_LH_Default_PFC_10	216	0.15737643	0.29624001
7Networks_RH_SomMot_19	304	0.15692239	0.29765333
7Networks_RH_SomMot_26	311	0.1536531	0.3079579
7Networks_RH_Vis_17	267	-0.1529775	0.31011527
7Networks_RH_Cont_PFCl_1	424	-0.1527517	0.31083861
7Networks_LH_Default_pCunPCC_11	247	-0.1525051	0.31162972
7Networks_LH_SalVentAttn_FrOperIns_6	123	0.15018561	0.31913315
7Networks_RH_Cont_PFCl_16	439	-0.1495267	0.32128538
7Networks_RH_SomMot_39	324	0.14866359	0.32411832
7Networks_RH_DorsAttn_FEF_1	359	0.1486193	0.32426411
7Networks_RH_SalVentAttn_Med_5	389	-0.1469901	0.32965584
7Networks_LH_SomMot_4	43	0.14467493	0.33741342
7Networks_LH_Cont_Par_6	160	-0.1446235	0.337587
7Networks_LH_Default_PFC_1	207	0.14447198	0.3380988
7Networks_LH_Cont_pCun_2	178	0.1443758	0.33842391
7Networks_RH_SomMot_29	314	0.14348092	0.34145818
7Networks_RH_SalVentAttn_FrOperIns_4	378	0.14294436	0.34328553
7Networks_LH_Default_Temp_3	188	-0.1427788	0.34385049
7Networks_RH_SalVentAttn_Med_1	385	-0.1427211	0.34404757
7Networks_RH_Cont_Cing_2	448	-0.1424266	0.34505457
7Networks_LH_Limbic_OFC_1	139	-0.1416834	0.34760372
7Networks_LH_Limbic_OFC_2	140	-0.1413799	0.34864804
7Networks_LH_SomMot_14	53	0.14084378	0.35049725
7Networks_RH_Default_Temp_9	469	0.14068998	0.35102887
7Networks_LH_Cont_pCun_3	179	0.1406368	0.35121283
7Networks_LH_Cont_OFC_1	166	0.14023029	0.35262084
7Networks_LH_SalVentAttn_Med_3	131	0.13976691	0.35423002

7Networks_RH_SomMot_34	319	0.13967132	0.35456255
7Networks_RH_Cont_PFCl_4	427	0.13959808	0.35481745
7Networks_RH_Default_Temp_6	466	0.13923633	0.35607808
7Networks_LH_Cont_PFCmp_1	183	-0.1388405	0.3574605
7Networks_RH_DorsAttn_PrCv_2	364	0.13723939	0.36308606
7Networks_LH_Cont_Par_8	162	0.13662577	0.36525612
7Networks_LH_Vis_16	16	0.13598329	0.36753657
7Networks_RH_Cont_Temp_1	421	0.13556968	0.36900918
7Networks_RH_Vis_14	264	0.13423398	0.37378893
7Networks_LH_DorsAttn_Post_7	93	0.13292154	0.37852127
L_Default	507	-0.1315901	0.38335849
7Networks_LH_Default_Temp_2	187	0.13105177	0.38532441
7Networks_LH_Vis_30	30	0.12974216	0.39013206
7Networks_RH_Cont_PFCl_15	438	0.12922729	0.39203175
7Networks_RH_Default_PFCdPFCm_15	489	-0.1273137	0.39913951
7Networks_LH_Default_PFC_30	236	0.12713078	0.39982301
7Networks_RH_SalVentAttn_TempOccPar_	368	0.12611092	0.40364538
7Networks_RH_Default_pCunPCC_3	492	0.12585216	0.40461852
7Networks_LH_SalVentAttn_FrOperIns_2	119	0.12567789	0.40527468
7Networks_RH_Default_pCunPCC_4	493	0.12535316	0.40649902
7Networks_RH_Default_PFCdPFCm_13	487	-0.1252425	0.40691683
7Networks_RH_SomMot_11	296	0.12456082	0.40949532
7Networks_LH_SomMot_29	68	0.12454823	0.40954303
7Networks_LH_Vis_9	9	0.12433079	0.41036753
7Networks_RH_Default_Par_6	458	-0.1233422	0.4141284
7Networks_LH_SomMot_2	41	0.12154718	0.4210068
7Networks_RH_Vis_15	265	0.12133611	0.42181982
7Networks_RH_DorsAttn_Post_4	338	0.12120957	0.4223077
7Networks_LH_Default_pCunPCC_5	241	-0.1194368	0.42917611
7Networks_RH_Limbic_OFC_7	404	0.11871291	0.43199844
7Networks_LH_Default_pCunPCC_10	246	-0.1173526	0.43733023
7Networks_RH_Vis_24	274	0.11684288	0.43933763
7Networks_LH_Limbic_TempPole_9	153	-0.1159964	0.44268225
7Networks_RH_SalVentAttn_PrC_1	374	0.11534945	0.44524812
7Networks_LH_SomMot_32	71	0.1152068	0.44581495
7Networks_RH_SalVentAttn_TempOccPar_	369	0.11508401	0.4463032
7Networks_RH_Default_PFCdPFCm_4	478	-0.1146967	0.44784529
7Networks_RH_Cont_PFCl_20	443	0.11456264	0.44837954
7Networks_LH_Default_PHC_1	249	0.11447645	0.44872329
7Networks_LH_Default_PFC_22	228	-0.1143061	0.44940319
7Networks_RH_Cont_PFCl_3	426	-0.1140942	0.45024975
7Networks_RH_Vis_25	275	0.11396722	0.45075729
7Networks_LH_Default_pCunPCC_2	238	-0.1138084	0.45139264
7Networks_LH_Vis_11	11	0.11229186	0.45748441
7Networks_LH_Cont_PFCl_1	167	-0.1122828	0.457521
7Networks_LH_Default_PFC_24	230	0.1121362	0.45811216

7Networks_RH_Cont_Par_3	417	-0.1116839	0.45993903
7Networks_LH_SomMot_1	40	0.11084936	0.46331984
7Networks_LH_Vis_32	32	0.10895326	0.47105078
7Networks_LH_Default_PFC_19	225	-0.1087281	0.47197324
7Networks_LH_Default_PFC_23	229	-0.1085551	0.47268273
7Networks_LH_SalVentAttn_ParOper_5	116	0.10670058	0.48032415
7Networks_RH_SalVentAttn_TempOccPar_	370	0.10626062	0.48214643
7Networks_RH_SomMot_5	290	0.1046834	0.48870887
7Networks_RH_Limbic_OFC_6	403	-0.1043139	0.49025278
7Networks_LH_Cont_Cing_3	182	-0.1041393	0.4909834
7Networks_LH_Vis_26	26	0.10294603	0.49599109
7Networks_LH_Cont_PFCmp_2	184	-0.1025913	0.49748469
7Networks_RH_SalVentAttn_FrOperIns_8	382	0.10160202	0.50166266
7Networks_RH_Default_PFCdPFCm_9	483	-0.1012636	0.50309584
7Networks_LH_SomMot_6	45	-0.1011065	0.50376182
7Networks_RH_SalVentAttn_Med_12	396	0.10046082	0.50650427
7Networks_LH_Vis_33	33	0.10002892	0.50834278
7Networks_LH_Limbic_TempPole_6	150	0.09961774	0.51009624
7Networks_LH_Default_Par_3	200	0.09807144	0.51671761
7Networks_LH_Cont_PFCl_7	173	0.09528573	0.52875369
7Networks_LH_Default_PFC_3	209	0.09475986	0.53104117
7Networks_RH_Default_pCunPCC_5	494	0.09376775	0.53536988
7Networks_RH_SomMot_23	308	0.09323957	0.53768137
7Networks_LH_Vis_10	10	-0.0932308	0.53771984
7Networks_RH_Default_pCunPCC_11	500	-0.0926677	0.54018928
7Networks_RH_SalVentAttn_TempOccPar_	373	0.09155603	0.54508122
7Networks_LH_Default_PFC_4	210	-0.0902398	0.55090027
7Networks_LH_Vis_22	22	-0.0899054	0.55238349
7Networks_LH_Vis_17	17	-0.089551	0.55395734
7Networks_RH_DorsAttn_FEF_2	360	0.08886704	0.55700076
7Networks_LH_Default_PFC_17	223	0.08808652	0.56048341
7Networks_RH_SomMot_7	292	0.08651518	0.56752535
7Networks_RH_Cont_PFCl_17	440	0.086074	0.56950983
7Networks_LH_Default_PFC_12	218	0.08602797	0.56971704
7Networks_RH_Default_Par_3	455	-0.0855725	0.5717693
7Networks_LH_SomMot_15	54	0.08539792	0.57255711
7Networks_RH_Cont_PFCl_9	432	-0.085394	0.57257495
7Networks_LH_Vis_24	24	0.08398604	0.57894479
R_Frontoparietal	513	-0.0834086	0.58156638
7Networks_LH_SomMot_12	51	0.08283747	0.58416489
7Networks_RH_Default_PFCdPFCm_1	475	-0.0820635	0.58769453
7Networks_RH_Default_PFCdPFCm_7	481	-0.081891	0.5884823
7Networks_RH_Cont_PFCl_11	434	0.0807548	0.59368393
7Networks_RH_Cont_pCun_2	445	0.07901586	0.60168399
7Networks_RH_SalVentAttn_Med_3	387	-0.0777606	0.60748786
7Networks_LH_Cont_Par_7	161	0.07604547	0.61545675

7Networks_LH_Limbic_TempPole_10	154	0.07576692	0.61675511
7Networks_LH_SomMot_5	44	0.07492875	0.62066895
7Networks_RH_DorsAttn_Post_16	350	0.07484232	0.62107314
7Networks_LH_SalVentAttn_ParOper_3	114	0.07246987	0.63221042
7Networks_RH_Vis_3	253	0.07191197	0.6348413
7Networks_RH_Default_pCunPCC_1	490	-0.0714666	0.63694453
7Networks_LH_SomMot_46	85	0.06993232	0.6442125
7Networks_RH_SalVentAttn_FrOperIns_5	379	0.06991828	0.64427916
L_Limbic	505	0.06987798	0.64447054
7Networks_LH_SomMot_24	63	-0.0690087	0.64860354
7Networks_LH_DorsAttn_PrCv_2	111	0.06877883	0.64969825
7Networks_RH_Default_PFCv_3	473	0.06875932	0.64979121
7Networks_LH_Cont_Cing_1	180	-0.0685468	0.65080421
7Networks_LH_DorsAttn_FEF_2	107	0.06776015	0.65455817
7Networks_LH_Default_PFC_21	227	0.06590542	0.66344302
R_Default	514	-0.0640317	0.67246527
7Networks_LH_Cont_Par_5	159	0.06367	0.67421246
7Networks_RH_Vis_22	272	-0.0635641	0.6747241
7Networks_LH_Cont_PFCl_8	174	-0.0631208	0.67686811
7Networks_RH_Cont_Cing_3	449	0.06203162	0.68214685
7Networks_RH_Default_pCunPCC_9	498	0.06165026	0.68399867
7Networks_LH_Cont_PFCmp_3	185	0.0596204	0.69388573
7Networks_RH_SalVentAttn_PFCl_1	384	-0.0595652	0.69415537
7Networks_LH_Default_PFC_28	234	-0.0589282	0.69726898
7Networks_LH_Default_Par_8	205	-0.0586786	0.69849013
7Networks_RH_Default_PFCdPFCm_12	486	-0.0573941	0.70478778
7Networks_LH_Default_PFC_27	233	0.05596173	0.71183319
7Networks_LH_Default_PFC_9	215	0.05593141	0.71198261
R_Ventral_attention	511	-0.0554908	0.71415479
7Networks_LH_Vis_21	21	-0.0552494	0.7153461
7Networks_LH_Default_Temp_10	195	0.05474562	0.71783363
7Networks_LH_Default_PFC_29	235	0.05399946	0.72152365
7Networks_RH_Limbic_OFC_2	399	0.05391111	0.72196101
7Networks_LH_Default_pCunPCC_7	243	-0.053279	0.72509251
7Networks_LH_Default_PFC_5	211	-0.0528603	0.72716906
7Networks_RH_Vis_10	260	0.05068635	0.73798277
7Networks_LH_DorsAttn_PrCv_1	110	0.04765217	0.75315877
7Networks_LH_Cont_PFCl_9	175	0.0458041	0.76244799
7Networks_LH_Limbic_OFC_5	143	0.04571722	0.76288553
7Networks_RH_Cont_PFCl_12	435	0.04527421	0.76511762
7Networks_LH_SalVentAttn_FrOperIns_8	125	-0.0447427	0.76779832
R_Dorsa_lattention	510	-0.0442503	0.77028386
7Networks_LH_Cont_Temp_1	163	0.04389386	0.77208453
7Networks_RH_Default_Temp_4	464	-0.0429464	0.77687697
7Networks_RH_Default_PFCdPFCm_10	484	0.04268837	0.77818348
7Networks_RH_Limbic_TempPole_9	413	0.04225728	0.78036768

L_Somatomotor	502	0.04203903	0.78147419
7Networks_RH_SalVentAttn_FrOperIns_9	383	-0.0417181	0.78310179
7Networks_RH_Default_Temp_7	467	-0.0378192	0.80294922
7Networks_RH_Cont_PFCv_1	423	-0.0370429	0.806916
7Networks_RH_Limbic_TempPole_8	412	-0.0370341	0.8069607
7Networks_LH_SomMot_8	47	0.0367054	0.80864185
7Networks_RH_DorsAttn_PrCv_1	363	0.03574669	0.81354994
7Networks_LH_SalVentAttn_PFCl_1	128	0.03528026	0.81594034
7Networks_LH_Cont_PFCl_5	171	0.03496463	0.81755887
7Networks_LH_Default_pCunPCC_8	244	0.03413051	0.82183979
7Networks_LH_Vis_20	20	-0.0340886	0.82205517
R_Limbic	512	-0.0315106	0.83531852
7Networks_LH_Vis_25	25	-0.0312693	0.83656228
7Networks_LH_SalVentAttn_Med_4	132	0.03042402	0.84092271
7Networks_LH_Default_Par_5	202	-0.0291933	0.84727966
7Networks_RH_Cont_Par_4	418	0.0283547	0.85161679
7Networks_RH_SomMot_48	333	0.02824433	0.85218792
7Networks_RH_Cont_Par_1	415	-0.027972	0.85359725
7Networks_LH_Default_PFC_18	224	0.0274874	0.85610686
7Networks_LH_Vis_15	15	-0.0274054	0.85653151
7Networks_LH_Default_pCunPCC_1	237	0.02727906	0.85718612
7Networks_RH_Limbic_OFC_5	402	-0.0262501	0.86252012
7Networks_LH_Cont_Cing_2	181	-0.0250633	0.86867995
7Networks_LH_Vis_23	23	-0.0237386	0.87556488
R_Somatomotor	509	0.02267039	0.88112305
7Networks_RH_Cont_Temp_2	422	0.02198286	0.88470356
7Networks_LH_SomMot_3	42	-0.0219012	0.88512916
7Networks_RH_Cont_PFCmp_3	452	0.02104959	0.88956743
L_Dorsa_lattention	503	-0.0209772	0.88994463
7Networks_RH_SalVentAttn_Med_7	391	-0.020819	0.89076993
7Networks_RH_SomMot_28	313	0.01946372	0.89784156
7Networks_RH_Cont_Par_2	416	0.01875472	0.90154421
7Networks_LH_Cont_PFCl_3	169	0.01777369	0.90667091
7Networks_LH_SalVentAttn_FrOperIns_3	120	0.01730767	0.9091076
7Networks_LH_SalVentAttn_Med_5	133	0.01671151	0.91222592
7Networks_LH_Vis_37	37	-0.0166667	0.91246042
7Networks_RH_Default_PFCdPFCm_14	488	0.01560092	0.91803852
7Networks_LH_Vis_5	5	-0.0150351	0.92100163
7Networks_RH_Vis_20	270	-0.0144089	0.92428205
7Networks_LH_Cont_PFCl_6	172	0.01376283	0.92766788
7Networks_LH_SalVentAttn_FrOperIns_5	122	0.01227075	0.93549215
7Networks_LH_SomMot_26	65	0.01200402	0.93689148
7Networks_LH_Default_Par_6	203	0.01084146	0.94299281
7Networks_LH_Default_pCunPCC_6	242	-0.008151	0.95712456
7Networks_LH_Default_PFC_20	226	-0.0078769	0.95856525
7Networks_LH_Limbic_OFC_4	142	-0.0071837	0.96220908

7Networks_LH_Cont_PFCl_2	168	-0.0068298	0.96406924
7Networks_LH_Default_pCunPCC_3	239	0.00638882	0.96638792
7Networks_LH_Vis_2	2	-0.0060511	0.96816384
7Networks_LH_Default_Par_7	204	-0.0057917	0.96952768
7Networks_RH_Cont_PFCl_7	430	-0.0053175	0.97202178
7Networks_LH_Vis_27	27	0.0044258	0.97671208
7Networks_RH_Limbic_OFC_3	400	-0.0040596	0.97863848
7Networks_RH_Default_pCunPCC_8	497	0.00264331	0.98609007
7Networks_LH_Default_pCunPCC_4	240	0.00227394	0.98803363
7Networks_LH_Cont_Par_4	158	-0.0019837	0.98956114

Supplemental table 3: Gene Ontology Analyses
Partial corelation analysis: Upweighted

term_name	source	Pvalue
presynapse	GO:CC	4.84E-09
somatodendritic compartment	GO:CC	6.85E-09
synaptic membrane	GO:CC	1.75E-08
potassium ion transmembrane transporter activity	GO:MF	2.11E-08
presynaptic membrane	GO:CC	3.93E-08
voltage-gated potassium channel complex	GO:CC	7.57E-08
axon	GO:CC	1.52E-07
voltage-gated potassium channel activity	GO:MF	2.06E-07
potassium channel complex	GO:CC	2.17E-07
voltage-gated ion channel activity	GO:MF	2.78E-07
voltage-gated channel activity	GO:MF	2.95E-07
transmembrane transporter complex	GO:CC	3.45E-07
gated channel activity	GO:MF	4.06E-07
cation channel complex	GO:CC	5.74E-07
potassium channel activity	GO:MF	6.74E-07
transporter complex	GO:CC	1.00E-06
ion channel complex	GO:CC	2.01E-06
potassium ion transport	GO:BP	2.28E-06
anterograde trans-synaptic signaling	GO:BP	3.96E-06
chemical synaptic transmission	GO:BP	3.96E-06
cell body	GO:CC	5.33E-06
trans-synaptic signaling	GO:BP	6.19E-06
metal ion transmembrane transporter activity	GO:MF	8.06E-06
postsynapse	GO:CC	1.42E-05
voltage-gated cation channel activity	GO:MF	1.56E-05
neuronal cell body	GO:CC	1.79E-05
potassium ion transmembrane transport	GO:BP	1.81E-05
cation channel activity	GO:MF	1.92E-05
ion channel activity	GO:MF	2.01E-05
inorganic cation transmembrane transporter activity	GO:MF	2.38E-05
synaptic signaling	GO:BP	2.49E-05
intrinsic component of synaptic membrane	GO:CC	2.75E-05
channel activity	GO:MF	4.50E-05
passive transmembrane transporter activity	GO:MF	4.50E-05
cation transmembrane transporter activity	GO:MF	7.46E-05
inorganic molecular entity transmembrane transporter activity	GO:MF	8.18E-05
perikaryon	GO:CC	8.77E-05
inorganic ion transmembrane transport	GO:BP	8.84E-05
regulation of ion transmembrane transport	GO:BP	0.00012348
integral component of synaptic membrane	GO:CC	0.00013702
dendrite	GO:CC	0.00019233
dendritic tree	GO:CC	0.00021141
postsynaptic membrane	GO:CC	0.00024493
regulation of transmembrane transport	GO:BP	0.00034243

ion transmembrane transporter activity	GO:MF	0.00047455
glutamatergic synapse	GO:CC	0.00048683
cation transmembrane transport	GO:BP	0.00050057
main axon	GO:CC	0.00066239
inorganic cation transmembrane transport	GO:BP	0.0007702
signal release	GO:BP	0.00118457

Partial corelation analysis: Downweighted

term_name	source	Pvalue
cell morphogenesis involved in differentiation	GO:BP	0.00027335
I band	GO:CC	0.00289009
phosphatidylinositol-4,5-bisphosphate binding	GO:MF	0.00368941
camera-type eye development	GO:BP	0.00554273
cell morphogenesis involved in neuron differentiation	GO:BP	0.00664151
muscle system process	GO:BP	0.00744241
Z disc	GO:CC	0.00946266
sarcoplasmic reticulum	GO:CC	0.0129431
positive regulation of early endosome to late endosome transport	GO:BP	0.01294843
focal adhesion	GO:CC	0.01324269
anchoring junction	GO:CC	0.01332473
GTPase complex	GO:CC	0.01485732
heterotrimeric G-protein complex	GO:CC	0.01485732
cell-substrate junction	GO:CC	0.01719553
NOTCH3 Intracellular Domain Regulates Transcription	REAC	0.01850608
sarcomere	GO:CC	0.01872779
sensory organ development	GO:BP	0.02260757
blood circulation	GO:BP	0.0230073
sarcoplasm	GO:CC	0.0282932
sarcolemma	GO:CC	0.02845776
outflow tract morphogenesis	GO:BP	0.03009194
phosphatidylinositol bisphosphate binding	GO:MF	0.03037171
heart process	GO:BP	0.03186337
cellular component morphogenesis	GO:BP	0.03351805
positive regulation of secretion	GO:BP	0.03358401
adherens junction	GO:CC	0.03471267
acetylcholine receptor signaling pathway	GO:BP	0.03604844
voltage-gated calcium channel complex	GO:CC	0.03720565
cellular response to acetylcholine	GO:BP	0.04109745
response to acetylcholine	GO:BP	0.04109745
aortic valve morphogenesis	GO:BP	0.04419623
myofibril	GO:CC	0.0470758
neurotransmitter receptor activity	GO:MF	0.0488204
Notch signaling pathway	GO:BP	0.05038699
alpha-actinin binding	GO:MF	0.05060507
plasma membrane bounded cell projection morphogenesis	GO:BP	0.05143039
ventricular septum morphogenesis	GO:BP	0.05196986
axonogenesis	GO:BP	0.05560889
cell projection morphogenesis	GO:BP	0.05664756
calcium channel complex	GO:CC	0.05837548
G-protein beta/gamma-subunit complex	GO:CC	0.05867515
dopamine receptor signaling pathway	GO:BP	0.06152581
contractile fiber	GO:CC	0.06312857
extracellular matrix	GO:CC	0.06340585

phosphatidylinositol phosphate binding	GO:MF	0.0639312
postsynaptic signal transduction	GO:BP	0.06459098
external encapsulating structure	GO:CC	0.06570448
circulatory system process	GO:BP	0.06855865
cellular response to dopamine	GO:BP	0.07010152
cochlea development	GO:BP	0.07207894

Group*NfL analysis: Upweighted

term_name	source	Pvalue
microtubule organizing center	GO:CC	0.00083305
plasma membrane bounded cell projecti	GO:BP	0.00120178
cell projection assembly	GO:BP	0.00187664
centrosome	GO:CC	0.00298564
cilium organization	GO:BP	0.00402457
cilium assembly	GO:BP	0.00717899
cyclic nucleotide metabolic process	GO:BP	0.00838778
centriole	GO:CC	0.01354388
microtubule-based process	GO:BP	0.02257078
synaptic vesicle lumen	GO:CC	0.03492152
synaptic vesicle	GO:CC	0.03500482
chromaffin granule	GO:CC	0.03604738
cortical actin cytoskeleton organization	GO:BP	0.0398374
exocytic vesicle	GO:CC	0.04985911
cyclic-nucleotide-mediated signaling	GO:BP	0.05647457
neuronal dense core vesicle lumen	GO:CC	0.06984119
dense core granule lumen	GO:CC	0.06984119
chromaffin granule lumen	GO:CC	0.06984119
CMG complex	GO:CC	0.07237423
histone acetyltransferase binding	GO:MF	0.07939022
activin receptor activity, type I	GO:MF	0.08303799
cAMP metabolic process	GO:BP	0.09177422
Neurotoxicity of clostridium toxins	REAC	0.09246044
presynapse	GO:CC	0.1076083
alcohol binding	GO:MF	0.10853259
calmodulin binding	GO:MF	0.1117565
formation of radial glial scaffolds	GO:BP	0.11570004
neuron to neuron synapse	GO:CC	0.12639553
activin receptor complex	GO:CC	0.13682233
ATPase-coupled intramembrane lipid tra	GO:MF	0.14763638
spectrin-associated cytoskeleton	GO:CC	0.15714683
DNA replication preinitiation complex	GO:CC	0.16173877
organelle assembly	GO:BP	0.16269881
nuclear body	GO:CC	0.16585403
dense core granule	GO:CC	0.17276827
membrane depolarization during cardia	GO:BP	0.18126378
SMAD binding	GO:MF	0.19558249
cytoplasmic side of plasma membrane	GO:CC	0.19648204
glycosyl compound biosynthetic process	GO:BP	0.2003773
C2H2 zinc finger domain binding	GO:MF	0.20605218
neurosecretory vesicle	GO:CC	0.22698986
isoprenoid catabolic process	GO:BP	0.23476334
general adaptation syndrome, behaviora	GO:BP	0.23990653
cell body fiber	GO:CC	0.24441195

negative regulation of myosin-light-chai	GO:BP	0.26134764
serine/threonine protein kinase complex	GO:CC	0.26848401
activin receptor signaling pathway	GO:BP	0.27430731
protein kinase complex	GO:CC	0.27544871
opioid peptide activity	GO:MF	0.27808705
vinculin binding	GO:MF	0.28460214

Group*NfL analysis: Downweighted

term_name	source	Pvalue
presynapse	GO:CC	2.13E-14
axon	GO:CC	3.07E-09
anterograde trans-synaptic signaling	GO:BP	3.31E-09
chemical synaptic transmission	GO:BP	3.31E-09
trans-synaptic signaling	GO:BP	5.36E-09
synaptic signaling	GO:BP	8.19E-09
modulation of chemical synaptic transr	GO:BP	9.69E-09
regulation of trans-synaptic signaling	GO:BP	1.05E-08
exocytic vesicle	GO:CC	1.77E-08
synaptic vesicle membrane	GO:CC	4.11E-08
exocytic vesicle membrane	GO:CC	4.11E-08
transport vesicle membrane	GO:CC	3.21E-07
intrinsic component of presynaptic mer	GO:CC	3.70E-07
neuron projection terminus	GO:CC	9.41E-07
synaptic vesicle	GO:CC	1.26E-06
transport vesicle	GO:CC	1.32E-06
intrinsic component of synaptic membr	GO:CC	2.23E-06
intrinsic component of synaptic vesicle	GO:CC	3.15E-06
anchored component of membrane	GO:CC	3.60E-06
anchored component of plasma memb	GO:CC	1.80E-05
axon terminus	GO:CC	1.83E-05
presynaptic membrane	GO:CC	3.10E-05
terminal bouton	GO:CC	3.37E-05
glutamatergic synapse	GO:CC	6.72E-05
distal axon	GO:CC	6.78E-05
synaptic membrane	GO:CC	0.000107
regulation of system process	GO:BP	0.00023694
Microtubule-dependent trafficking of co	REAC	0.00024514
somatodendritic compartment	GO:CC	0.00029106
Transport of connexons to the plasma r	REAC	0.00033867
perikaryon	GO:CC	0.00035009
integral component of presynaptic mer	GO:CC	0.00038967
anchored component of presynaptic me	GO:CC	0.00041069
Assembly and cell surface presentation	REAC	0.00042785
presynaptic active zone membrane	GO:CC	0.00056112
regulation of secretion	GO:BP	0.00061676
Post-chaperonin tubulin folding pathwa	REAC	0.00080721
intrinsic component of presynaptic acti	GO:CC	0.00084104
Formation of tubulin folding intermedi	REAC	0.00134456
integral component of synaptic membr	GO:CC	0.00148302
pyrophosphatase activity	GO:MF	0.001546
regulation of secretion by cell	GO:BP	0.00192943
Activation of AMPK downstream of NM	REAC	0.00214256
cell body	GO:CC	0.00220227

hydrolase activity, acting on acid anhyd	GO:MF	0.00230459
hydrolase activity, acting on acid anhyd	GO:MF	0.0023882
COPI-independent Golgi-to-ER retrogra	REAC	0.00239445
integral component of synaptic vesicle	GO:CC	0.00239957
channel regulator activity	GO:MF	0.00269674
regulation of neurotransmitter secretio	GO:BP	0.00288754

Partial corelation analysis 514 (comp2): Upweighted

term_name	source	Pvalue
metal ion transmembrane transporter activity	GO:MF	1.64E-10
inorganic cation transmembrane transporter activity	GO:MF	1.60E-09
transporter complex	GO:CC	2.45E-09
transmembrane transporter complex	GO:CC	3.15E-09
cation transmembrane transporter activity	GO:MF	5.81E-09
ion transmembrane transporter activity	GO:MF	6.56E-09
inorganic molecular entity transmembrane transporter activity	GO:MF	6.97E-09
potassium ion transmembrane transporter activity	GO:MF	1.03E-08
voltage-gated potassium channel complex	GO:CC	6.65E-08
cation channel complex	GO:CC	1.18E-07
ion channel complex	GO:CC	1.19E-07
voltage-gated potassium channel activity	GO:MF	1.96E-07
inorganic ion transmembrane transport	GO:BP	2.04E-07
cation channel activity	GO:MF	2.97E-07
potassium channel complex	GO:CC	3.09E-07
gated channel activity	GO:MF	4.10E-07
ion channel activity	GO:MF	1.02E-06
voltage-gated ion channel activity	GO:MF	1.19E-06
voltage-gated channel activity	GO:MF	1.30E-06
cation transmembrane transport	GO:BP	1.50E-06
inorganic cation transmembrane transport	GO:BP	4.54E-06
regulation of ion transmembrane transporter activity	GO:BP	4.78E-06
regulation of transmembrane transporter activity	GO:BP	5.72E-06
presynapse	GO:CC	6.12E-06
regulation of transmembrane transport	GO:BP	9.92E-06
potassium channel activity	GO:MF	1.07E-05
channel activity	GO:MF	1.18E-05
passive transmembrane transporter activity	GO:MF	1.18E-05
voltage-gated cation channel activity	GO:MF	1.19E-05
amide transport	GO:BP	1.24E-05
potassium ion transport	GO:BP	1.28E-05
synaptic signaling	GO:BP	1.55E-05
insulin secretion	GO:BP	1.91E-05
potassium ion transmembrane transport	GO:BP	2.11E-05
delayed rectifier potassium channel activity	GO:MF	3.22E-05
peptide hormone secretion	GO:BP	3.78E-05
trans-synaptic signaling	GO:BP	4.16E-05
regulation of insulin secretion	GO:BP	4.61E-05
glutamatergic synapse	GO:CC	5.64E-05
regulation of transporter activity	GO:BP	6.14E-05
peptide secretion	GO:BP	7.46E-05
regulation of ion transmembrane transport	GO:BP	9.05E-05
somatodendritic compartment	GO:CC	9.49E-05
peptide transport	GO:BP	0.00010681

synaptic membrane	GO:CC	0.00011144
Voltage gated Potassium channels	REAC	0.00013133
main axon	GO:CC	0.00015903
regulation of ion transport	GO:BP	0.00016409
P-type sodium transporter activity	GO:MF	0.00017367
kinesin binding	GO:MF	0.00131067

Partial corelation analysis 514 (comp1): Downweighted

term_name	source	Pvalue
stress response to metal ion	GO:BP	1.48E-10
Metallothioneins bind metals	REAC	2.94E-10
stress response to copper ion	GO:BP	4.35E-10
detoxification of copper ion	GO:BP	4.35E-10
cellular response to metal ion	GO:BP	1.24E-09
cellular response to zinc ion	GO:BP	3.59E-09
detoxification of inorganic compou	GO:BP	5.05E-09
cellular response to inorganic subs	GO:BP	5.76E-09
Response to metal ions	REAC	9.92E-09
transporter complex	GO:CC	2.57E-08
transmembrane transporter comp	GO:CC	2.88E-08
astrocyte projection	GO:CC	4.15E-08
synaptic membrane	GO:CC	6.06E-08
cellular response to cadmium ion	GO:BP	9.26E-08
cellular zinc ion homeostasis	GO:BP	2.45E-07
zinc ion homeostasis	GO:BP	3.64E-07
postsynapse	GO:CC	5.08E-07
integral component of synaptic me	GO:CC	6.88E-07
ion channel complex	GO:CC	7.06E-07
postsynaptic membrane	GO:CC	8.15E-07
cellular response to copper ion	GO:BP	8.40E-07
intrinsic component of synaptic m	GO:CC	1.1979E-06
gated channel activity	GO:MF	1.7533E-06
response to copper ion	GO:BP	2.4515E-06
response to toxic substance	GO:BP	3.0632E-06
metal ion transmembrane transpo	GO:MF	5.4629E-06
response to zinc ion	GO:BP	5.7383E-06
intrinsic component of postsynapti	GO:CC	6.6905E-06
transmitter-gated ion channel acti	GO:MF	8.218E-06
astrocyte end-foot	GO:CC	8.7447E-06
presynapse	GO:CC	9.1775E-06
integral component of postsynapti	GO:CC	1.0518E-05
intrinsic component of postsynapti	GO:CC	1.1883E-05
synaptic signaling	GO:BP	1.3642E-05
neurotransmitter receptor activity	GO:MF	1.4013E-05
inorganic molecular entity transm	GO:MF	1.4856E-05
ion transmembrane transporter ac	GO:MF	1.5395E-05
glial cell projection	GO:CC	1.9829E-05
response to metal ion	GO:BP	2.0265E-05
response to inorganic substance	GO:BP	2.4552E-05
trans-synaptic signaling	GO:BP	2.5992E-05
postsynaptic specialization	GO:CC	2.7167E-05
Mineral absorption	KEGG	2.8622E-05
integral component of postsynapti	GO:CC	3.3894E-05

response to cadmium ion	GO:BP	4.0474E-05
ligand-gated anion channel activit	GO:MF	4.5135E-05
transmitter-gated ion channel acti	GO:MF	5.1068E-05
transmitter-gated channel activity	GO:MF	5.1068E-05
cation channel complex	GO:CC	7.7289E-05
postsynaptic specialization memb	GO:CC	8.5798E-05

DOWNWEIGHTED
Top 10

	CellType	annotLevel	p	fold_change	sd_from_mean
Astrocyte	Astrocyte	1	0.00000	1.9080063	7.21517288
Non-neuronal:Pericyte	Non-neuronal:Pericyte	1	0.29683	1.0800290	0.49468718
Non-neuronal:VLMC	Non-neuronal:VLMC	1	0.33726	1.0736055	0.37335082
Microglia	Microglia	1	0.46827	1.0039485	0.02779101
OPC	OPC	1	0.52067	0.9858527	-0.12058863
Endothelial cell	Endothelial cell	1	0.80142	0.8673286	-0.85842662
GABAergic	GABAergic	1	0.84056	0.8915276	-0.99207555
Oligodendrocyte	Oligodendrocyte	1	0.99660	0.6928582	-2.32499395
Glutamatergic	Glutamatergic	1	0.99942	0.6733577	-2.82281849

Top 20

	CellType	annotLevel	p	fold_change	sd_from_mean
Astrocyte	Astrocyte	1	0.00000	1.7876626	9.3744629
OPC	OPC	1	0.03897	1.1386111	1.8448813
Non-neuronal:Pericyte	Non-neuronal:Pericyte	1	0.04083	1.1922916	1.7981916
Non-neuronal:VLMC	Non-neuronal:VLMC	1	0.11248	1.1574667	1.2358595
Endothelial cell	Endothelial cell	1	0.26185	1.0622104	0.6159431
Microglia	Microglia	1	0.63219	0.9650994	-0.3726639
GABAergic	GABAergic	1	0.99994	0.7553098	-3.3990788
Oligodendrocyte	Oligodendrocyte	1	0.99999	0.6839277	-3.6536741
Glutamatergic	Glutamatergic	1	1.00000	0.5640608	-5.7117988

Top 30

	CellType	annotLevel	p	fold_change	sd_from_mean
Astrocyte	Astrocyte	1	0.00000	1.7505631	10.8462748
OPC	OPC	1	0.00048	1.2259682	3.6255160
Non-neuronal:Pericyte	Non-neuronal:Pericyte	1	0.00285	1.2586386	2.9242836
Non-neuronal:VLMC	Non-neuronal:VLMC	1	0.00780	1.2661336	2.5447574
Endothelial cell	Endothelial cell	1	0.25222	1.0539451	0.6477374
Microglia	Microglia	1	0.79821	0.9345802	-0.8431500
GABAergic	GABAergic	1	1.00000	0.7252092	-4.5943438
Glutamatergic	Glutamatergic	1	1.00000	0.5400691	-7.2362923
Oligodendrocyte	Oligodendrocyte	1	1.00000	0.6754626	-4.5157716

UPWEIGHTED

Top 10

	CellType	annotLevel	p	fold_change	sd_from_mean
Glutamatergic	Glutamatergic	1	0.00000	2.8965723	16.3369659
GABAergic	GABAergic	1	0.00002	1.5357106	4.9597176
Non-neuronal:Pericyte	Non-neuronal:Pericyte	1	0.39915	1.0343656	0.2081843
OPC	OPC	1	0.85648	0.8778167	-1.0462340
Microglia	Microglia	1	0.98576	0.7163352	-1.9887524
Non-neuronal:VLMC	Non-neuronal:VLMC	1	0.99380	0.5787213	-2.2141690
Endothelial cell	Endothelial cell	1	0.99947	0.5744964	-2.7896588
Astrocyte	Astrocyte	1	0.99999	0.5661990	-3.5435030
Oligodendrocyte	Oligodendrocyte	1	1.00000	0.4965921	-3.8092236

Top 20

	CellType	annotLevel	p	fold_change	sd_from_mean
GABAergic	GABAergic	1	0.00000	1.5628554	7.2861417
Glutamatergic	Glutamatergic	1	0.00000	2.6069948	19.4543472
Non-neuronal:Pericyte	Non-neuronal:Pericyte	1	0.58670	0.9700696	-0.2530976
OPC	OPC	1	0.90035	0.8962679	-1.2557837
Microglia	Microglia	1	0.99820	0.7379222	-2.6098185
Non-neuronal:VLMC	Non-neuronal:VLMC	1	0.99987	0.5680338	-3.1902216
Endothelial cell	Endothelial cell	1	0.99997	0.6406164	-3.2668333
Astrocyte	Astrocyte	1	0.99999	0.6693298	-3.7748540
Oligodendrocyte	Oligodendrocyte	1	1.00000	0.5945374	-4.3385157

Top 30

	CellType	annotLevel	p	fold_change	sd_from_mean
GABAergic	GABAergic	1	0.00000	1.5667087	11.1405354
Glutamatergic	Glutamatergic	1	0.00000	2.4186899	26.0760206
OPC	OPC	1	0.82978	0.9485987	-0.9546552
Non-neuronal:Pericyte	Non-neuronal:Pericyte	1	0.96056	0.8666257	-1.7209779
Astrocyte	Astrocyte	1	1.00000	0.6498258	-6.0331864
Endothelial cell	Endothelial cell	1	1.00000	0.6989214	-4.1734454
Microglia	Microglia	1	1.00000	0.6756120	-4.8942728
Non-neuronal:VLMC	Non-neuronal:VLMC	1	1.00000	0.6397530	-3.9999783
Oligodendrocyte	Oligodendrocyte	1	1.00000	0.6459751	-5.7442214

DOWNWEIGHTED

Top 10

	CellType	annotLevel	p	fold_change	sd_from_mean
ASC	ASC	1	0.00000	2.1467224	8.6160309
NSC	NSC	1	0.00041	1.5438766	3.8787306
MG	MG	1	0.19379	1.1805605	0.8271783
END	END	1	0.30596	1.0730030	0.4690038
OPC	OPC	1	0.56731	0.9604670	-0.2637758
exDG	exDG	1	0.99954	0.7011287	-3.0771729
exCA	exCA	1	0.99968	0.7236111	-3.1453997
ODC	ODC	1	0.99997	0.5286452	-2.7900722
GABA	GABA	1	0.99999	0.5986439	-4.0608297
exPFC	exPFC	1	1.00000	0.4460789	-6.3925347

Top 20

	CellType	annotLevel	p	fold_change	sd_from_mean
ASC	ASC	1	0.00000	2.1216158	13.019927
NSC	NSC	1	0.00000	1.6275235	6.818819
END	END	1	0.01936	1.2210205	2.169183
OPC	OPC	1	0.08958	1.1311786	1.391230
MG	MG	1	0.14017	1.1553144	1.090851
exDG	exDG	1	0.99991	0.7850779	-3.399237
exCA	exCA	1	1.00000	0.7338652	-4.656273
exPFC	exPFC	1	1.00000	0.4860334	-9.106682
GABA	GABA	1	1.00000	0.6639972	-5.175448
ODC	ODC	1	1.00000	0.6029644	-3.616080

Top 30

	CellType	annotLevel	p	fold_change	sd_from_mean
ASC	ASC	1	0.00000	1.9853333	13.8533048
NSC	NSC	1	0.00000	1.6515618	8.6199562
END	END	1	0.00112	1.2760383	3.2762132
OPC	OPC	1	0.00150	1.2649235	3.3896482
MG	MG	1	0.20928	1.0922923	0.7862396
exCA	exCA	1	1.00000	0.7696426	-4.8986370
exDG	exDG	1	1.00000	0.7456277	-4.8532094
exPFC	exPFC	1	1.00000	0.5090226	-10.5343093
GABA	GABA	1	1.00000	0.6626227	-6.3320011
ODC	ODC	1	1.00000	0.6517338	-3.8833481

UPWEIGHTED

Top 10

	CellType	annotLevel	
exPFC	exPFC	1	[illegible]
GABA	GABA	1	[illegible]
exCA	exCA	1	[illegible]
exDG	exDG	1	[illegible]
OPC	OPC	1	[illegible]
MG	MG	1	[illegible]
END	END	1	[illegible]
NSC	NSC	1	[illegible]
ASC	ASC	1	[illegible]
ODC	ODC	1	[illegible]

Top 20

	CellType	annotLevel	
exPFC	exPFC	1	[illegible]
GABA	GABA	1	[illegible]
exCA	exCA	1	[illegible]
exDG	exDG	1	[illegible]
OPC	OPC	1	[illegible]
MG	MG	1	[illegible]
END	END	1	[illegible]
NSC	NSC	1	[illegible]
ASC	ASC	1	[illegible]
ODC	ODC	1	[illegible]

Top 30

	CellType	annotLevel	
exCA	exCA	1	[illegible]
exPFC	exPFC	1	[illegible]
GABA	GABA	1	[illegible]
exDG	exDG	1	[illegible]
OPC	OPC	1	[illegible]
ASC	ASC	1	[illegible]
END	END	1	[illegible]
MG	MG	1	[illegible]
NSC	NSC	1	[illegible]
ODC	ODC	1	[illegible]

p	fold_change	sd_from_mean
0.00000	2.4348545	16.6485585
0.00000	1.5126969	5.3622511
0.02088	1.1765958	2.0846464
0.42426	1.0154205	0.1634997
0.93015	0.8045365	-1.3392742
0.99099	0.6058762	-1.8305720
0.99148	0.6692891	-2.1302382
0.99976	0.5996654	-2.9559775
1.00000	0.5955889	-3.1781351
1.00000	0.3944621	-3.6076633

p	fold_change	sd_from_mean
0.00000	2.2944546	21.255695
0.00000	1.5237175	7.772176
0.00021	1.2239890	3.700548
0.08964	1.0911934	1.361902
0.97868	0.8128728	-1.819054
0.99494	0.6788113	-2.126149
0.99952	0.6723502	-2.949116
0.99999	0.6403570	-3.709428
1.00000	0.6268100	-4.105162
1.00000	0.5019625	-4.203144

p	fold_change	sd_from_mean
0.00000	1.2861125	7.111902
0.00000	2.0815982	26.979486
0.00000	1.5037216	11.185578
0.00227	1.1290119	2.896102
0.99792	0.8272499	-2.575828
1.00000	0.6554980	-5.714896
1.00000	0.6024826	-5.456631
1.00000	0.6089863	-3.949403
1.00000	0.6341874	-5.670090
1.00000	0.5013421	-6.384944

DOWNWEIGHTED
Top 10

	CellType	annotLevel	p	fold_change	sd_from_mean
Glutamatergic	Glutamatergic	1	0.00000	2.8327490	15.3434863
GABAergic	GABAergic	1	0.00010	1.4754347	4.2783200
OPC	OPC	1	0.20687	1.0899718	0.7888829
Non-neuronal:VLMC	Non-neuronal:VLMC	1	0.98562	0.6028899	-1.9850374
Endothelial cell	Endothelial cell	1	0.98776	0.6848202	-2.0268150
Non-neuronal:Pericyte	Non-neuronal:Pericyte	1	0.99747	0.5892623	-2.4548210
Astrocyte	Astrocyte	1	0.99978	0.6348877	-2.9696604
Oligodendrocyte	Oligodendrocyte	1	0.99990	0.6004525	-2.9841701
Microglia	Microglia	1	0.99993	0.5594399	-3.0422093

Top 20

	CellType	annotLevel	p	fold_change	sd_from_mean
Glutamatergic	Glutamatergic	1	0.00000	2.4650611	17.3050555
GABAergic	GABAergic	1	0.00001	1.3810154	4.8313313
OPC	OPC	1	0.52588	0.9914191	-0.1043843
Non-neuronal:Pericyte	Non-neuronal:Pericyte	1	0.90601	0.8457699	-1.2940746
Endothelial cell	Endothelial cell	1	0.98485	0.7748456	-2.0277082
Non-neuronal:VLMC	Non-neuronal:VLMC	1	0.98580	0.7124474	-2.0496675
Microglia	Microglia	1	0.99974	0.6863739	-3.0728553
Astrocyte	Astrocyte	1	1.00000	0.6153164	-4.3801830
Oligodendrocyte	Oligodendrocyte	1	1.00000	0.5704611	-4.5762984

Top 30

	CellType	annotLevel	p	fold_change	sd_from_mean
GABAergic	GABAergic	1	0.00000	1.4520012	7.067176
Glutamatergic	Glutamatergic	1	0.00000	2.3781409	20.032593
OPC	OPC	1	0.86078	0.9277072	-1.077525
Endothelial cell	Endothelial cell	1	0.97029	0.8365796	-1.811780
Non-neuronal:Pericyte	Non-neuronal:Pericyte	1	0.97613	0.8161347	-1.898006
Non-neuronal:VLMC	Non-neuronal:VLMC	1	0.99862	0.6878437	-2.746087
Microglia	Microglia	1	0.99995	0.7114559	-3.487487
Astrocyte	Astrocyte	1	1.00000	0.6259048	-5.218574
Oligodendrocyte	Oligodendrocyte	1	1.00000	0.5852989	-5.420006

UPWEIGHTED

Top 10

	CellType	annotLevel	p	fold_change	sd_from_mean
Non-neuronal:Pericyte	Non-neuronal:Pericyte	1	0.03905	1.2885703	1.8584263
Microglia	Microglia	1	0.06393	1.2154728	1.5984608
GABAergic	GABAergic	1	0.06428	1.1669625	1.5819017
Non-neuronal:VLMC	Non-neuronal:VLMC	1	0.06945	1.2674849	1.5287391
Endothelial cell	Endothelial cell	1	0.24742	1.0972362	0.6532038
Oligodendrocyte	Oligodendrocyte	1	0.77574	0.9005163	-0.7809531
Glutamatergic	Glutamatergic	1	0.87605	0.8707324	-1.1348463
OPC	OPC	1	0.97856	0.7974082	-1.8417790
Astrocyte	Astrocyte	1	0.99049	0.7471473	-2.0875882

Top 20

	CellType	annotLevel	p	fold_change	sd_from_mean
Non-neuronal:VLMC	Non-neuronal:VLMC	1	0.06168	1.2017341	1.5791780
Microglia	Microglia	1	0.06727	1.1544516	1.5454835
Endothelial cell	Endothelial cell	1	0.09240	1.1469956	1.3590359
Non-neuronal:Pericyte	Non-neuronal:Pericyte	1	0.10909	1.1419000	1.2483287
GABAergic	GABAergic	1	0.17655	1.0708236	0.9242551
Oligodendrocyte	Oligodendrocyte	1	0.37953	1.0243077	0.2624645
Astrocyte	Astrocyte	1	0.77207	0.9328777	-0.7614940
OPC	OPC	1	0.92198	0.8904074	-1.3741212
Glutamatergic	Glutamatergic	1	0.98145	0.8406591	-1.9664894

Top 30

	CellType	annotLevel	p	fold_change	sd_from_mean
Endothelial cell	Endothelial cell	1	0.02644	1.1748014	2.00879163
Microglia	Microglia	1	0.04667	1.1392084	1.74155805
Non-neuronal:VLMC	Non-neuronal:VLMC	1	0.04823	1.1761973	1.70688324
GABAergic	GABAergic	1	0.07446	1.0908201	1.47389253
Oligodendrocyte	Oligodendrocyte	1	0.15121	1.0773709	1.03109609
Non-neuronal:Pericyte	Non-neuronal:Pericyte	1	0.46082	1.0066377	0.07272635
Astrocyte	Astrocyte	1	0.85820	0.9235997	-1.06837505
Glutamatergic	Glutamatergic	1	0.95231	0.8942189	-1.61434718
OPC	OPC	1	0.98118	0.8727598	-1.97430333

DOWNWEIGHTED

Top 10

	CellType	annotLevel	p	fold_change	sd_from_mean
exPFC	exPFC	1	0.00000	2.0779226	12.3441758
exDG	exDG	1	0.00022	1.3666441	3.7928874
exCA	exCA	1	0.00066	1.3107977	3.4764304
GABA	GABA	1	0.00122	1.3490730	3.4681711
OPC	OPC	1	0.61980	0.9423225	-0.3907471
NSC	NSC	1	0.99952	0.6123936	-2.7889430
MG	MG	1	0.99980	0.4846588	-2.3241966
ASC	ASC	1	1.00000	0.5222577	-3.6486546
END	END	1	1.00000	0.4305421	-3.6784713
ODC	ODC	1	1.00000	0.4023971	-3.4606696

Top 20

	CellType	annotLevel	p	fold_change	sd_from_mean
exCA	exCA	1	0.00000	1.3594772	5.782224
exDG	exDG	1	0.00000	1.4152133	6.083587
exPFC	exPFC	1	0.00000	1.9296582	14.995064
GABA	GABA	1	0.00086	1.2405557	3.439142
OPC	OPC	1	0.99034	0.7887323	-2.051694
MG	MG	1	0.99951	0.6071117	-2.518176
NSC	NSC	1	0.99999	0.6088343	-3.964776
ASC	ASC	1	1.00000	0.6164604	-4.184850
END	END	1	1.00000	0.5703684	-3.875863
ODC	ODC	1	1.00000	0.4182302	-4.857167

Top 30

	CellType	annotLevel	p	fold_change	sd_from_mean
exCA	exCA	1	0.00000	1.4910906	9.676854
exDG	exDG	1	0.00000	1.4493801	8.016227
exPFC	exPFC	1	0.00000	1.7825785	15.431363
GABA	GABA	1	0.00007	1.2454933	4.285946
OPC	OPC	1	0.99991	0.7373575	-3.078132
MG	MG	1	0.99995	0.6265609	-2.963004
ASC	ASC	1	1.00000	0.5776908	-5.653179
END	END	1	1.00000	0.5707191	-4.750707
NSC	NSC	1	1.00000	0.6509231	-4.339257
ODC	ODC	1	1.00000	0.4126747	-6.014396

UPWEIGHTED

Top 10

	CellType	annotLevel	
GABA	GABA	1	[illegible]
END	END	1	[illegible]
MG	MG	1	[illegible]
ODC	ODC	1	[illegible]
NSC	NSC	1	[illegible]
exPFC	exPFC	1	[illegible]
exDG	exDG	1	[illegible]
ASC	ASC	1	[illegible]
exCA	exCA	1	[illegible]
OPC	OPC	1	[illegible]

Top 20

	CellType	annotLevel	
GABA	GABA	1	[illegible]
END	END	1	[illegible]
ASC	ASC	1	[illegible]
ODC	ODC	1	[illegible]
MG	MG	1	[illegible]
exDG	exDG	1	[illegible]
OPC	OPC	1	[illegible]
exPFC	exPFC	1	[illegible]
NSC	NSC	1	[illegible]
exCA	exCA	1	[illegible]

Top 30

	CellType	annotLevel	
GABA	GABA	1	[illegible]
ODC	ODC	1	[illegible]
END	END	1	[illegible]
MG	MG	1	[illegible]
ASC	ASC	1	[illegible]
exDG	exDG	1	[illegible]
OPC	OPC	1	[illegible]
NSC	NSC	1	[illegible]
exPFC	exPFC	1	[illegible]
exCA	exCA	1	[illegible]

p	fold_change	sd_from_mean
0.00055	1.3460461	3.7673937
0.16965	1.1440916	0.9491042
0.27699	1.1014413	0.5224541
0.35309	1.0464418	0.2949299
0.43425	1.0160966	0.1217495
0.55124	0.9880577	-0.1477211
0.58470	0.9789899	-0.2354829
0.68571	0.9341149	-0.5353249
0.71331	0.9530157	-0.5745261
0.82824	0.8689903	-0.9386904

p	fold_change	sd_from_mean
0.00072	1.2410096	3.596424790
0.05804	1.1788425	1.623876143
0.15276	1.0901832	1.025278517
0.18578	1.1004006	0.875082210
0.34167	1.0497538	0.340673463
0.36248	1.0218467	0.339102435
0.47047	1.0008496	0.008553936
0.84042	0.9417848	-0.994319292
0.84316	0.9037603	-1.003840773
0.93748	0.9104505	-1.513658723

p	fold_change	sd_from_mean
0.00035	1.2006214	3.7235889
0.11537	1.1139745	1.2203891
0.11726	1.1066497	1.2032972
0.15267	1.1205147	1.0268015
0.37181	1.0203887	0.2838541
0.55139	0.9925133	-0.1427981
0.72080	0.9500683	-0.6149799
0.79843	0.9345122	-0.8480626
0.95069	0.9227636	-1.6219300
0.99154	0.8879396	-2.3380632

DOWNWEIGHTED

Top 10

	CellType	annotLevel	p	fold_change	sd_from_mean
Astrocyte	Astrocyte	1	0.00000	1.9099668	9.9036727
GABAergic	GABAergic	1	0.00225	1.2467922	3.0617743
OPC	OPC	1	0.01984	1.1884461	2.1958451
Non-neuronal:Pericyte	Non-neuronal:Pericyte	1	0.26416	1.0740768	0.6117709
Glutamatergic	Glutamatergic	1	0.27470	1.0492885	0.5785578
Non-neuronal:VLMC	Non-neuronal:VLMC	1	0.81685	0.8679592	-0.9103710
Microglia	Microglia	1	0.98494	0.7849050	-2.0270704
Endothelial cell	Endothelial cell	1	0.99811	0.6993795	-2.6086313
Oligodendrocyte	Oligodendrocyte	1	1.00000	0.5084314	-5.1297793

Top 20

	CellType	annotLevel	p	fold_change	sd_from_mean
Astrocyte	Astrocyte	1	0.00000	1.6678656	10.3979220
GABAergic	GABAergic	1	0.00037	1.2061982	3.6843733
OPC	OPC	1	0.00336	1.1727641	2.8981173
Glutamatergic	Glutamatergic	1	0.06382	1.0924693	1.5539662
Non-neuronal:Pericyte	Non-neuronal:Pericyte	1	0.11070	1.1040232	1.2351758
Non-neuronal:VLMC	Non-neuronal:VLMC	1	0.35926	1.0335868	0.3356472
Microglia	Microglia	1	0.99724	0.8087930	-2.5936479
Endothelial cell	Endothelial cell	1	0.99978	0.7380583	-3.2619542
Oligodendrocyte	Oligodendrocyte	1	1.00000	0.6239295	-5.5984651

Top 30

	CellType	annotLevel	p	fold_change	sd_from_mean
Astrocyte	Astrocyte	1	0.00000	1.6236733	11.9953744
OPC	OPC	1	0.00012	1.1865377	3.8735390
Non-neuronal:Pericyte	Non-neuronal:Pericyte	1	0.02508	1.1363697	2.0064255
GABAergic	GABAergic	1	0.02539	1.0916485	2.0097964
Non-neuronal:VLMC	Non-neuronal:VLMC	1	0.30981	1.0386519	0.4767441
Glutamatergic	Glutamatergic	1	0.39253	1.0125092	0.2574896
Microglia	Microglia	1	0.99232	0.8607943	-2.3252665
Endothelial cell	Endothelial cell	1	0.99756	0.8252676	-2.6941259
Oligodendrocyte	Oligodendrocyte	1	1.00000	0.6224616	-6.9423685

UPWEIGHTED COMP 2

Top 10

	CellType	annotLevel	p	fold_change	sd_from_mean
GABAergic	GABAergic	1	0.00000	1.5298501	6.6060444
Glutamatergic	Glutamatergic	1	0.00000	1.7743251	8.8827558
OPC	OPC	1	0.43657	1.0098987	0.1118536
Non-neuronal:Pericyte	Non-neuronal:Pericyte	1	0.85698	0.8672835	-1.0628124
Non-neuronal:VLMC	Non-neuronal:VLMC	1	0.90556	0.8121003	-1.2861677
Endothelial cell	Endothelial cell	1	0.94758	0.8195575	-1.5611951
Oligodendrocyte	Oligodendrocyte	1	0.98643	0.7999207	-2.0322556
Microglia	Microglia	1	0.99274	0.7588011	-2.2528792
Astrocyte	Astrocyte	1	0.99868	0.7503415	-2.7068906

Top 20

	CellType	annotLevel	p	fold_change	sd_from_mean
GABAergic	GABAergic	1	0.00000	1.5575952	9.6965444
Glutamatergic	Glutamatergic	1	0.00000	1.8014568	13.0189863
Non-neuronal:Pericyte	Non-neuronal:Pericyte	1	0.76100	0.9365161	-0.7209452
OPC	OPC	1	0.85395	0.9350950	-1.0514201
Endothelial cell	Endothelial cell	1	0.97858	0.8426170	-1.9371926
Non-neuronal:VLMC	Non-neuronal:VLMC	1	0.99617	0.7437469	-2.4986356
Oligodendrocyte	Oligodendrocyte	1	0.99800	0.8157171	-2.6690364
Microglia	Microglia	1	0.99812	0.8007469	-2.6701879
Astrocyte	Astrocyte	1	0.99987	0.7765498	-3.4115024

Top 30

	CellType	annotLevel	p	fold_change	sd_from_mean
GABAergic	GABAergic	1	0.00000	1.5287761	11.1960237
Glutamatergic	Glutamatergic	1	0.00000	1.7232157	14.2917375
Non-neuronal:Pericyte	Non-neuronal:Pericyte	1	0.55423	0.9885587	-0.1584914
OPC	OPC	1	0.78444	0.9595062	-0.7987163
Non-neuronal:VLMC	Non-neuronal:VLMC	1	0.97063	0.8473712	-1.8278596
Endothelial cell	Endothelial cell	1	0.99746	0.8233560	-2.6569925
Microglia	Microglia	1	0.99871	0.8267294	-2.8322074
Oligodendrocyte	Oligodendrocyte	1	0.99923	0.8332501	-2.9423782
Astrocyte	Astrocyte	1	0.99967	0.8280602	-3.2085716

DOWNWEIGHTED

Top 10

	CellType	annotLevel	p	fold_change	sd_from_mean
ASC	ASC	1	0.00000	1.7078752	7.4073510
NSC	NSC	1	0.00000	1.5570671	5.5122299
exDG	exDG	1	0.06136	1.1108652	1.5676784
OPC	OPC	1	0.06984	1.1637849	1.5513354
END	END	1	0.26593	1.0686263	0.6053314
GABA	GABA	1	0.64788	0.9697864	-0.4103370
MG	MG	1	0.73736	0.8894998	-0.6780234
exCA	exCA	1	0.93007	0.9071109	-1.4464286
exPFC	exPFC	1	0.99873	0.8178724	-2.8639220
ODC	ODC	1	1.00000	0.5298466	-3.8633011

Top 20

	CellType	annotLevel	p	fold_change	sd_from_mean
ASC	ASC	1	0.00000	1.6376036	9.6026124
NSC	NSC	1	0.00000	1.4766447	6.7545338
OPC	OPC	1	0.01443	1.1746416	2.3616564
exDG	exDG	1	0.03603	1.0911695	1.8371285
exCA	exCA	1	0.08152	1.0632651	1.4070763
END	END	1	0.19893	1.0660536	0.8356654
GABA	GABA	1	0.28180	1.0283059	0.5522869
MG	MG	1	0.84521	0.8873266	-1.0106065
exPFC	exPFC	1	0.99993	0.8383965	-3.6252942
ODC	ODC	1	1.00000	0.5673612	-5.0377607

Top 30

	CellType	annotLevel	p	fold_change	sd_from_mean
ASC	ASC	1	0.00000	1.6399033	11.8811846
NSC	NSC	1	0.00000	1.4805654	8.3202058
OPC	OPC	1	0.00003	1.2909545	4.8348283
exDG	exDG	1	0.00623	1.1032328	2.5556418
exCA	exCA	1	0.13076	1.0411459	1.1205608
END	END	1	0.17060	1.0608365	0.9494211
MG	MG	1	0.37461	1.0258121	0.2821243
GABA	GABA	1	0.88488	0.9506523	-1.1859089
exPFC	exPFC	1	1.00000	0.8319244	-4.5792596
ODC	ODC	1	1.00000	0.5890299	-5.8928622

UPWEIGHTED COMP 2

Top 10

	CellType	annotLevel	p	fold_ch[illegible]
exPFC	exPFC	1	0.00000	1.7[illegible]
GABA	GABA	1	0.00000	1.7[illegible]
exCA	exCA	1	0.01698	1.1[illegible]
exDG	exDG	1	0.54350	0.9[illegible]
OPC	OPC	1	0.76556	0.9[illegible]
END	END	1	0.95123	0.8[illegible]
MG	MG	1	0.96152	0.7[illegible]
ODC	ODC	1	0.99744	0.7[illegible]
NSC	NSC	1	0.99918	0.7[illegible]
ASC	ASC	1	0.99932	0.7[illegible]

Top 20

	CellType	annotLevel	p	fold_[illegible]
exPFC	exPFC	1	0.00000	1.7[illegible]
GABA	GABA	1	0.00000	1.6[illegible]
exCA	exCA	1	0.00655	1.1[illegible]
exDG	exDG	1	0.24801	1.0[illegible]
END	END	1	0.97914	0.8[illegible]
MG	MG	1	0.98380	0.7[illegible]
OPC	OPC	1	0.99386	0.8[illegible]
ASC	ASC	1	0.99977	0.7[illegible]
NSC	NSC	1	0.99985	0.7[illegible]
ODC	ODC	1	0.99988	0.7[illegible]

Top 30

	CellType	annotLevel	p	fold_[illegible]
exPFC	exPFC	1	0.00000	1.6[illegible]
GABA	GABA	1	0.00000	1.5[illegible]
exCA	exCA	1	0.00023	1.1[illegible]
exDG	exDG	1	0.15880	1.0[illegible]
OPC	OPC	1	0.94771	0.9[illegible]
END	END	1	0.98969	0.8[illegible]
MG	MG	1	0.99174	0.8[illegible]
NSC	NSC	1	0.99891	0.8[illegible]
ASC	ASC	1	0.99984	0.8[illegible]
ODC	ODC	1	1.00000	0.7[illegible]

ange	sd_from_mean
09965	11.9630807
95572	10.3518065
06945	2.1822204
07254	-0.1283326
27743	-0.7542807
46798	-1.5855764
58408	-1.5809090
08129	-2.2955673
69130	-2.7978758
79271	-2.7081510

hange	sd_from_mean
25771	16.8364342
16801	12.3091751
49940	2.5484563
39619	0.6732148
88482	-1.9495893
54797	-1.9258968
35865	-2.2371093
67084	-3.1247744
65491	-3.3251460
10797	-3.1676521

hange	sd_from_mean
91198	17.623476
53555	13.782002
92680	3.722270
17311	1.002633
00269	-1.557072
02427	-2.216221
16566	-2.179110
51835	-2.916165
57175	-3.287050
65282	-3.648905

	ROI 114	ROI 514
**NBS Analyses**	NFL - fMRI correlation (whole group), p = 0.0304<br>NFL - fMRI correlation (preHD), p = 0.019	NFL - fMRI correlation (whole group), p = 0.0398<br>NFL - fMRI correlation (preHD), p = 0.027
**Gene Ontology**	**Upweighted (top 5) GO terms:**<br>presynapse (p = 4.84x10^{-9})<br>somatodendritic compartment (p = 6.85x10^{-9})<br>synaptic membrane (p = 1.75x10^{-9})<br>potassium ion transmembrane transporter activity (p = 2.11x10^{-8})<br>presynaptic membrane (p = 3.93x10^{-8})<br><br>*No upweighted genes where identified in the 1st PLS component for ROI 514 analysis, thus the 2nd PLS component was also invesitagted	**Upweighted* (component 2 (C2)) (overlap) GO terms:**<br>potassium ion transmembrane transporter activity (p = 1.03x10^{-8})<br>presynapse (p = 6.12x10^{-6})<br>somatodendritic compartment (p = 9.49x10^{-5})<br><br>**Downweighted* (component 1 (C1)) (overlap) GO terms:**<br>potassium ion transmembrane transporter activity (p = 1.03x10^{-8})<br>presynapse (p = 6.12x10^{-6})<br>somatodendritic compartment (p = 9.49x10^{-5})
**EWCE**	**Upweighted:**<br>GABAergic and glutamatergic (p<0.05)<br>**Downweighted:**<br>Astrocyte (p<0.05)	**Upweighted (C2):**<br>GABAergic and glutamatergic (p<0.05)<br>**Downweighted (C1):**<br>Astrocyte and GABAergic (p<0.05)
**HD gene enrichment**	Neuronal HD genes (p < 1x10^{-10})	Comp 2: Neuronal HD genes (p < 1x10^{-10})
**Spatial topography of PLS weights**	R (left to right): r = 0.02, p = 0.83<br>A (posterior to anterior): rho = -0.35, p = 0.0004<br>S (inferior to superior): rho = 0.42, p = 1.3x10^{-5}	R (left to right): C1; r = 0.18, p = 5.5x10^{-5}, C2; r = -0.21, p = 1.2x10^{-6}<br>A (posterior to Anterior): C1; r = -0.42, p = 2.2x10^{-16}, C2; r = -0.35, p = 5.8x10^{-16}<br>S (Inferior to superior): C1; r = 0.43, p = 2.2x10^{-16}, C2; r = 0.23, p = 2.2x10^{-7}
